# Transcriptome and Peptidome Characterisation of the Main Neuropeptides and Peptidic Hormones of a Euphausiid: The Ice Krill, *Euphausia crystallorophias*

**DOI:** 10.1371/journal.pone.0071609

**Published:** 2013-08-21

**Authors:** Jean-Yves Toullec, Erwan Corre, Benoît Bernay, Michael A. S. Thorne, Kévin Cascella, Céline Ollivaux, Joël Henry, Melody S. Clark

**Affiliations:** 1 UPMC University of Paris 06, UMR 7144 CNRS, Adaptation et Diversité en Milieu Marin, Station Biologique de Roscoff, Roscoff, France; 2 UPMC University of Paris 06, FR 2424 CNRS, ABiMS, Analysis and Bioinformatics for Marine Science, Station Biologique de Roscoff, Roscoff, France; 3 UPMC University of Paris 06, UMR 7150 CNRS, Mer et Santé, Station Biologique de Roscoff, Roscoff, France; 4 Centre National de la Recherche Scientifique, UMR 7144, Station Biologique de Roscoff, Roscoff, France; 5 Centre National de la Recherche Scientifique, UMR 7150, Station Biologique de Roscoff, Roscoff, France; 6 Université Européenne de Bretagne, UEB, France; 7 University of Caen Basse Normandie, FRE 3484 CNRS, Biologie des Mollusques Marins et des Ecosystèmes Associés, Caen, France; 8 University of Caen Basse Normandie, Plateforme PROTEOGEN, Caen, France, SF ICORE 4206; 9 British Antarctic Survey, Natural Environment Research Council, High Cross, Cambridge, United Kingdom; Swiss Institute of Bioinformatics, Switzerland

## Abstract

**Background:**

The Ice krill, *Euphausia crystallorophias* is one of the species at the base of the Southern Ocean food chain. Given their significant contribution to the biomass of the Southern Ocean, it is vitally important to gain a better understanding of their physiology and, in particular, anticipate their responses to climate change effects in the warming seas around Antarctica.

**Methodology/Principal Findings:**

Illumina sequencing was used to produce a transcriptome of the ice krill. Analysis of the assembled contigs via two different methods, produced 36 new pre-pro-peptides, coding for 61 neuropeptides or peptide hormones belonging to the following families: Allatostatins (A, B et C), Bursicon (α and β), Crustacean Hyperglycemic Hormones (CHH and MIH/VIHs), Crustacean Cardioactive Peptide (CCAP), Corazonin, Diuretic Hormones (DH), the Eclosion Hormone (EH), Neuroparsin, Neuropeptide F (NPF), small Neuropeptide F (sNPF), Pigment Dispersing Hormone (PDH), Red Pigment Concentrating Hormone (RPCH) and finally Tachykinin. LC/MS/MS proteomics was also carried out on eyestalk extracts, which are the major site of neuropeptide synthesis in decapod crustaceans. Results confirmed the presence of six neuropeptides and six precursor-related peptides previously identified in the transcriptome analyses.

**Conclusions:**

This study represents the first comprehensive analysis of neuropeptide hormones in a Eucarida non-decapod Malacostraca, several of which are described for the first time in a non-decapod crustacean. Additionally, there is a potential expansion of PDH and Neuropeptide F family members, which may reflect certain life history traits such as circadian rhythms associated with diurnal migrations and also the confirmation via mass spectrometry of several novel pre-pro-peptides, of unknown function. Knowledge of these essential hormones provides a vital framework for understanding the physiological response of this key Southern Ocean species to climate change and provides a valuable resource for studies into the molecular phylogeny of these organisms and the evolution of neuropeptide hormones.

## Introduction

Krill (Eucarida, Malacostraca) are a keystone species of the Southern Ocean food chain. The best-known member of this family of crustaceans is the Antarctic krill *stricto sensu Euphausia superba.* It is also the most abundant, forming 50% of the Antarctic zooplankton biomass of the Southern Ocean. However this family of Euphausiidae comprises several members [1], all having circumpolar distributions, with their range largely governed by temperature tolerances. Within the Southern Ocean, there is a decrease in water surface temperature with higher latitudes and the different species are present in a latitudinal succession. Hence they present ideal models for phylogenetically controlled studies to understand the underlying mechanisms into species resilience/sensitivity to global warming. *E. superba* occurs mainly south of 60°S, but north of 74°S, which is co-incident with the shelf-break waters. There is virtually no geographical overlap with the distribution of the Ice krill, *Euphausia crystallorophias.* This species is epi-pelagic and restricted to the inshore waters of the continental plateau at depths of less than 300 metres [2], where it is the dominant euphausiid. Whilst *E. superba* has been found in water temperatures up to 3.9°C, *E. crystallorophias* is more stenothermal and only found in waters below 0°C.

These two euphausiids clearly represent sentinel species in the study of the impact of global warming in this region. Molecular studies were initiated in order to gain a better understanding of their physiology and anticipate their responses in the face of diverse environmental stresses. A comprehensive knowledge of these genomes at the DNA level is unlikely in the near future due to their large sizes (approximately 33pg for *E. crystallorophias* and 48pg for *E. superba*) [3]. However, transcriptome sequencing, particularly using HiSeq technologies constitutes a very attractive option because sequence quantity outputs are continually increasing and costs decreasing. *E. superba* was the first of these two species to be sequenced [4]. In that study, 454 sequencing allowed the preliminary characterisation of putative stress genes, to identify potential molecular markers for environmental change. A similar global approach was also taken in this study, with *E. crystallorophias*, using the Next Generation Sequencing Illumina Hi-Seq platform. With this data we have been able to undertake a detailed study into the principal neuropeptides and neurohormones of both of these species.

This paper describes the characterisation of these sequences in both *E. crystallorophias* and *E. superba.* The previously published 454 transcriptome of *E. superba* was developed from RNA extracted from whole animals, whilst the Illumina Hi-Seq dataset of *E. crystallorophias* was generated from whole animal RNA supplemented with RNA from eyestalks. This latter strategy increased the success of identifying relevant transcripts, as these peptides are produced in the major neuroendocrine organ of the crustaceans, the X organ-sinus gland complex, which is located in the eyestalks. The peptides were chosen because of their physiological importance in crustaceans and insects, many were thought (but not proven) to be present in the neuroendocrine system of the crustaceans. The pre-pro-peptides were annotated using Blast2GO and were confirmed by alignment with orthologous sequences already identified in other species of crustaceans and hexapods. The sequence data was further supplemented with proteomic mass spectrometry analysis on *E. crystallorophias* eyestalk extracts. The peptide sequencing results were used to validate the assemblies and also confirm the predicted gene structures of the neuropeptides and their associated cryptic peptides. The majority (90%) of these peptides have never been characterised in the Malacostraca outside of the Decapoda or within the Crustacea with the main exceptions of the daphnias, *Daphnia magna* [5] and *D. pulex* [6], [7] with genome data available for the latter (www.fleabase.org) (Table 1).

**Table 1:** List of mature peptides and precursor related peptides (PRP) of *E. crystallorophias* and *E. superba*.

Peptide name	Peptide sequence	Previous identification in Arthropods	Pfam/Interpro accession N°	
Referenced peptides	Generic peptides			
**Corticotropin RF family**		Daphnia	PF00473/IPR000187	
Euc-CRFLDH56	GWRGLGARYARSRPQGLSLSIDASMKVLREALYLEIARKKQRQHQLRAAHNHQLLQ–			
**Neuropeptide F/Y family**		Daphnia, decapod, insects	PF00159/IPR001955	
**Euc-NPF1**	**KPDPTQLAAMADAIKYLQELDKYYSQVARPRFa**	Daphnia,Decapod (*L. vannamei)*		
Euc-NPF1-L	KPDPTQLAAMADAIKYLQELDKYYSQVARPSTRSAPSTGAGKIDVLENTLKFLQLQELGKLYNVRARPRFa	Decapod (*Litopenaeus vannamei)*		
Euc-NPF2	- - - - - - - - - - - - - - - - -YLELLNRYYAIAGRPRFa	None		
**Euc-NPF1-PRP**	**SEYAMAPRDALMEASEKLMETFEHQR**	Decapod (*Litopenaeus vannamei)*		
**Tachykinin Related Pept.**		Daphnia, decapods and insects,	-/IPR013206	
**Euc-TKRP**	**APSGFLGMRa**		-	
**Euc-TKRP-PRP**	**pQVDPLSDALDQNQLAQTLYDYRD**	None	-	
	**Arthropod specific peptides**			
**Allatostatins A family**		Cirriped, copepod, daphnia, decapods and insects	PF05953/IPR010276	
Euc-AST A1	IPGYSFGLa			
Euc-AST A2	QRQNKAYSFGLa			
**Euc-AST A3**	**ARNYAFGIa**			
Euc-AST A4	AKSYAFGLa			
Euc-AST A5	ANNMYSFGLa			
Euc-AST A6	GDGYNFGLa			
Euc-AST A7	DNSYGFGLa			
Euc-AST A8	GGNNMYGFGLa			
Euc-AST A9	GGKSYGFGLa			
Euc-AST A10	GKGAYSFGLa			
Euc-AST A11	GGDKMYGFGLa			
Euc-AST A12	ASDYGFGLa			
Euc-AST A13	VRDPYSFGLa			
Euc-AST A14	EPYAFGLa			
Euc-AST A15	SYDFGLa			
Euc-AST A16	SGPYSFGIa			
**Euc-AST A-PRP1**	**QDTSSQIDTQQLLQALRELRELY**SSRGYTFGN	Cirriped, copepod, decapods, insects		
Euc-CCAP	PFCNAFTGCa	Ixod, daphnia, decapods and insects	PF11105/IPR024276	
CHH family		Daphnia, isopod, decapods and insects	PF01147/IPR001166	
Euc-CHH	SIFDPSCKGFYNKEVFKKLNHICDDCYNLYRDASVAVKCKENCFGNPVFEQCIYELLIDDQVDELSKIVRTLa			
Euc-MIH/VIH1	NVAHLSSCGSLAGQRHIHRQVEQICLDCDNLYRQSRAGYNCRQSCYANPHFELCVHDLLLSHRVMEFRLLISMLQASL	Isopod, decapods		
Euc-MIH/VIH2	CGSIFGQRHIALKVEQVCRDCENLSRNYQTAFNCRKDCYTSETYTKCL –	Decapods (peneids)		
**Corazonin family**		Ixod, daphnia, decapods and insects	-/IPR020190	
**Euc-Arg^7^-CRZ1**	**pQTFQYSRGWTNa**			
*Eus-Arg^7^-CRZ1*	*pQTFQYSRGWTNa*			
Euc- Ser^4^- Arg^7^ -CRZ2	pQTFSYSRGWTNa	None		
*Eus- Ser^4^- Arg^7^ -CRZ2*	*pQTFEYSRGWTNa*	*idem*		
**Euc-Arg^7^-CRZ1-PRP3**	**MMIQQSFEERIRNLEQELGDVY**	Insects (*A. gambiae*, *T. castaneum*)		
Euc-Eclosion Hormone	SYTGMCIRNCGQCKDMYGAYFNSQSCAESCIMSQGNSVPDCNNPSTFKNFL	Cirriped, daphnia, decapods and insects	-/IPR006825	
**Neuroparsin family**		Copepod, daphnia and insects	PF07327/IPR010850	
Euc-NP1	APNCDVGNDVNPETCKYGTVRNWCRHSVCAKGPSEVCGGRWMQHGTCGTGTRCNCNRCLGCSSFTLECYTGGQVC			
*Eus-NP1*	*APNCDTDEGTDVNPETCKYGTVRNWCRHMVCAKGPGDVCGGRWMQHGSCGTGTRCNCNRCLGCSSTTLECYTGGQFC*	*idem*		
Euc-NP2	GEPVNEAACKFGVAMDWCRQVCAKGPGETCGGRWMQHGQCGDGLRCSCSRCSGCSPVTLDCFYGQFC	Copepod, daphnia		
*Eus-NP2*	*APNCDVAVGEPVNEKTCKFGVAMDWCRRQVCAKGPGESCGGHWMQHGQCSEGLRCSCNRCSGCSPVTLDCFYGQFC*	*idem*		
**Pigment Dispersing Hormone family**		Cirriped,daphnia, amphipod, isopod, decapods and insects	PF06324/IPR009396	
Euc-PDH-L α	NSGTINSMLGLPRTYNLRRMMMHAa	None		
*Eus-PDH-L α*	*NSGTINSMLGLPRTYNLRRMMMNAa*	*None*		
**Euc-PDH-Lβ1**	**NSELINSMLGLPQTLRAQKLMANMa**	None		
**Euc-PDH-L β2**	**NAETINTMLGLPQTLRAQKLMAKL**	None		
Euc-PDH α	NSELINSLLGLPKVMNDAa	-		
*Eus-PDH β*	*NSELINSLLGLPKVMNDAa*	*-*		
**Euc-PDH-L β1-PRP**	**pQEDQERQAVGNLALDILRVVGRAPSAMQ**	None		
**Euc-PDH-L β2-PRP**	**pQEDQERQVVGELALGILRIVGQESSGPQ**	None		
Euc-RPCH/AKH	pQLNFSPGWa	Daphnia, decapods and insects	PF06377/IPR010475	
Unreferenced peptides				
Allatostatin B family		Shrimp		
Euc-AST B1	DLRSVSPRSTNWSSLRGAWa		-	
Euc-AST B2	GGPNNWSNLRGAWa		-	
Euc-AST B3	GGPGDWGSFRGSWa		-	
Euc-AST B4	GGPGDWSNFRGSWa		-	
Euc-AST B5	GGADTDWNSFRGSWa		-	
**Allatostatin C family**		Cirriped, dapnia, decapods and insects		
Euc-AST C	SYWKQCAFNAVSCFa		-	
*Eus-AST C*	*SYWKQCAFNA- - - -*		*-*	
**Bursicon family**				
Euc-Bursicon *α*	VDECSLTPVIHILSYPGCKSKPIPSFACQGRCTSYVQVSGSKIWQTERSCMCCQESGEREAAVTLNCPKARSGEPKLKKVLTRAPIDCMCRPCTEVEAGAVMAQEIANFIGSNNMGDVPFLK	Cirriped, daphnia, decapods and insects	-	
*Eus-Bursicon α*	*VDECSLTPVIHILSYPGCKSKPIPSFACQGRCTSYVQVSGSKIWQTERSCMCCQESGEREAAVTLNCPKARSGEPKLKKVPARA--*	*idem*	*-*	
Euc-Bursicon β	KHYGTECETLPSTIHIVKEEFDDAGQVTVNCEEDIAVNKCEGACLSKVQPSVNTPSGFLKDCRCCRETHLRTREVTLNHCYDGDGNRLSGEKGKVQVKLREPADCQCYKCGDSNR	Cirriped, daphnia, decapods and insects	-	
**Calcitonin-Like Diuretic H.**				
Euc-CLDH31	GLDLGLGRGFSGSQAAKHLMGMAAANFAGGPa	Ixod, Cirriped, copepod, daphnia, lobster and insects	-	
Euc-CLDH33	VQMLDLGLGRGFSGAQAGKHLIGLLAASAAGGPa	None	-	
Euc-CHH PRP	RNIEPLNNDAMASLLSVANFKHVPAVS	Decapods	-	
Euc-SIFamide	GYRKPPFNGSIFa	Ixod, daphnia, decapods and insects	-	
**Small Neuropeptide F**				
Euc-sNPF1-1	SPSMRLRFa	Ixod, daphnia, decapods and insects	-	
Euc-sNPF1-2	DYWQVSQRSMPAVRLRFa	idem	-	
Euc-sNPF1-3	NFQQIPMESSLINDKDTRSPQLRLRFa	idem	-	
Euc-sNPF1-4	APDHGEILPFHELGTSSLGSEIYQKSIRSPQLRLRFa	Idem	-	
Euc-sNPF1-5	EDDADQEWTREMSNAALLDELLAPKELRSPQLRLRFa	Idem	-	
Euc-sNPF2-5	- - - - - - - - - - - - - - - - - - - - -DELMAPKALRSPQLRLRFa	Idem	-	
Euc-sNPF1-6	EPDNQYEQLLDQIEQKDTRSPKLRLRFa	Idem	-	
Euc-sNPF2-6	EPDNQYEQLPNQIEQKDTRSPKLRLRFa	Idem	-	
Euc-sNPF1-7	DQQVEDFDNDSGLSDAVNQKSIRSPQLRLRFa	Idem	-	
Euc-sNPF2-7	DQQIEDFDNDTGLADAVDQKSIWSPQLRLRFa	idem	-	

The peptides of *E. superba* are in italic and underlined. The peptides and the PRPs identified in the peptidome of *E. crystallorophias* eyestalks are highlighted in bold. a  =  amide; amphipod  =  *Talitrus saltator*; cirriped  =  *Amphibalanus amphitrite*; daphnia  =  *Daphnia pulex*; decapods  =  identified in more than two species of decapods; insects  =  identified in more than two species of hexapods; isopod  =  *Armadillidium vulgare*; Ixod  =  *Ixodus scapularis*; lobster  =  *Homarus americanus*;

Preliminary identifications were carried out in *E. crystallorophias*, the results of which were then used as a database to interrogate the *E. superba* transcriptome.

## Materials and Methods

### Animal collection

The ice krill, *Euphausia crystallorophias* were collected close to the Dumont d'Urville station in Terre Adélie (66°S411-102°E430), in the austral summer of 2010, by the deployment of an IKMT zooplankton net from the L'Astrolabe vessel. Krill swarms were located using an echo sounder and the sampling achieved by towing a net through the swarm for 10 to 15 min at a speed of 2 knots. The animals were flash frozen in liquid nitrogen and stored at −80°C. Six whole animals were used for the RNA extractions. These samples were supplemented withmaterial from eyestalks, one of the main organs for the production of neuropeptides. The eyestalks were partially dissected to remove the pigmented regions and then snap frozen in liquid nitrogen. Approximately 20 eyestalks were used to enrich the transcriptome, with a similar number used in the proteomic extractions. This project (IPEV-1039) was approved by IPEV (Institut Paul Emile Victor, the French polar institute) review committee and was declared to and approved by the “Terres Australes et Antarctiques Françaises” in 2009 according the Annex I of the Madrid Protocol and the French Decret No 2005-403. No endangered or protected species were used.

### Illumina sequencing

The sequencing process included mRNA isolation from whole animals and 20 eyestalks using the SV Total RNA Isolation System (Promega, Madison, WI, USA). Sequencing was conducted by the McGill University and Génome Québec Innovation Centre (Montréal, Québec, Canada) following the manufacturer's instructions (Illumina, San Diego, CA). These data have been submitted to the SRA-EBI with Accession number (ERR264582.

### RNA-Seq data sets

The RNA libraries yielded 15.3 million paired end reads with a maximum read length of 96 bp. Raw reads were filtered and trimmed using the FASTX-toolkit (Version 0.0.13 from Assaf Gordon Hannon lab) and rRNA contamination removed using ribopicker [8] and cutadapt (Version 1.1) [9], with a final quality check performed using fastQC (Version 0.10.0 http://www.bioinformatics.bbsrc.ac.uk/projects/fastqc/).

The contigs were assembled using two strategies leading to two transcriptome assemblies. The goal was to obtain the most comprehensive view of the whole transcriptome of *E. crystallorophias*. By using two assembly strategies and combining the results, our aim was to reduce the errors inherent in each of the technologies.

The first assembly was performed using Newbler (version 2.6) [10] and a paired end assembly strategy. Redundancy was determined by self-Blasting and the use of CD-HIT (95% similarity) [11].

The second assembly was performed using Trinity (release 2011-10-29) [12], the genome-independent transcriptome assembler. The assembler was run with following parameters (kmer size of 25 and minimum contig length of 300). Redundancy of the second assembly was reduced by using CD-HIT (95% similarity). The transcripts from both assemblies were processed through the Blast2GO pipeline [13] to produce putative annotations and functional classifications based on Blastx results against the GenBank NR database release 190 and InterProScan analysis against the InterPo database release 37.0.

Relative abundances of all the transcripts resulting from both assemblies were estimated by remapping the reads on each assembly with Bowtie [14] and performing RSEM abundance estimation [15].

### Peptide selection

A local database of annotated peptides, with their corresponding sequences was developed. The peptides were chosen based on the most highly characterised neuropeptide and neurohormone sequences in the Arthropods [16]–[19], with particular reference to the *D. pulex* genome [7]. A maximum expected size for the mature peptide was set at 130aa. In the first instance, relevant Blast2Go annotations from the Trinity assembly were identified. Each identified contig was then Blast searched independently to confirm the annotation and further verified in the Newbler assembly. The contigs were then translated and the putative coding sequences delineated. These sequences were then subjected to a Blastp search and subsequently aligned with orthologous sequences from the Arthropods (mainly the insects and the crustaceans). A sequence identified in *E. crystallorophias* and not identified in the annotation of the *E. superba* transcriptome was then systematically searched by local Blast alignment against the *E. superba* transcriptome. All of the Blast search data and alignments were performed in CLC Main Workbench. The signal peptides were identified using SignalP.

### Mass spectrometry

Two types of peptide extraction were applied. The first used cold 0.1% TFA, the second, a mixture of acetic acid, water and methanol (1:9:90). The extracts were pooled and then concentrated on a microTIP C18 (OMIX, VARIAN). The peptide eluate was either directly injected into a nano-LC and analysed by MS or was subjected to a trypsin digestion, with a separation stage in the nano-LC according to the following protocol: Endogenic peptides were reduced in 10 mM DTT at 60°C for 45 min and alkylated in 100 mM iodoacetamide for 30 min in the dark. Digestion was performed by adding 25 µl of 6 ng/µl trysin (Promega, USA) in 25 mM NH_4_HCO_3_. Samples were incubated at 37°C for 15 h. After digestion, peptides were collected and dried in a SpeedVac evaporator. Samples were resuspended in TFA 0.1% before nano-LC fractionation. The chromatography step was performed on a nano-LC system (Prominence, Shimadzu). Peptides were concentrated on a Zorbax 5×0.3mm C18 precolumn (Agilent) and separated onto a ACE 50×0.5mm C18 column (AIT, France). Mobile phases consisted of 0.1% acetic acid, 99.9% water (v/v) (A) and 0.1% acetic acid, 20% water in 79.9% ACN (v/v/v) (B). The nanoflow rate was set at 800 nl/min, and the gradient profile was as follows: constant 5% B for 5 min, from 5 to 100% B in 75 min, constant 100% B for 20 min, and return to 10% B. The 800 nl/min volume of the peptide solution was mixed with 1.6 µL/min volumes of solutions of 5mg/ml CHCA matrix prepared in a diluted solution of 50% CAN with 0.1% TFA. Fifteen second fractions were spotted by an AccuSpot spotter (Shimadzu) on a stainless steel Opti-TOF™ 384 target.

MS experiments were carried out on an AB Sciex 5800 proteomics analyzer equipped with TOF/TOF ion optics and an OptiBeam™ on-axis laser irradiation with 1000 Hz repetition rate. The system was calibrated immediately before analysis with a mixture of des-Arg-Bradykinin, Angiotensin I, Glu1-Fibrinopeptide B, ACTH (18–39), ACTH (7–38) and mass precision was greater than 50 ppm. All acquisitions were taken in automatic mode. A laser intensity of 3000 was typically employed for ionizing. MS spectra were acquired in the positive reflector mode by combining 1000 single spectra (5×200) in the mass range from 600 to 4000 Da. MS/MS spectra were acquired in the positive MS/MS reflector mode by combining a maximum of 2500 single spectra (10×250) with a laser intensity of 3900. For the tandem MS experiments, the acceleration voltage applied was 1 kV and air was used as the collision gas with gas pressure set to medium. The fragmentation pattern was used to determine the sequence of the peptide. Database searching was performed using the Mascot 2.2.04 program (Matrix Science) on the transcriptome database of *E. crystallorophias* (including the 42,632 contigs from the Trinity assemblies). The variable modifications allowed were as follows: C-terminal amidation, N-terminal pyroglutamate, N-terminal acetylation, methionine oxidation and dioxidation. “No enzyme” was selected. Mass accuracy was set to 100 ppm and 0.6 Da for MS and MS/MS mode respectively.

## Results and Discussion

### RNAseq assemblies

Quality processing of the raw reads led to the generation of 14.4 million good quality paired end reads. Two transcriptome assemblies were performed using the Newbler and Trinity assemblers. For the first assembly (Newbler), reads were assembled into 88,067 contigs with lengths varying from a minimum of 100 nt to 7,588 nt with a mean length of 289 nt, then reduced by CD-HIT clustering to a set of 21,425 contigs with lengths varying from a minimum of 300 nt to 7,588 nt with a mean length of 669 nt.

For the second assembly (Trinity), reads were assembled into 42,632 contigs of lengths varying from a minimum of 300 nt to 8,341 nt with a mean length of 760 nt, then rendundancy was reduced by clustering with CD-HIT to a set of 36,345 contigs with lengths varying from a minimum of 300 nt to 8,341 nt with a mean length of 698 nt.

Clustering of the 57,770 transcripts generated by the two approaches (36,345 Trinity transcripts and the 21,425 Newbler transcripts) at 95% of similarity indicated that 16,426 transcripts of the Newbler assembly (76.7%) clustered with 40.2% of the transcripts generated by Trinity (14,619/36,345) for a total of 14,178 mixed clusters. In 85% of the “mixed” clusters (2,128/14,178) the longest sequence was generated by the Trinity assembly. It was noteworthy that whilst there was greater diversity in the Trinity assembly, 4,999 contigs of the Newbler assembly were not detected (according to the CDHIT clustering) within the Trinity assembly. So to be as exhaustive as possible in cataloging the full transcriptome of *Euphausia crystallorophias*, annotations and further studies were performed on all the transcripts generated by both assemblers.

Expression levels of the transcripts in the assemblies were estimated by remapping the quality reads on both transcriptomes separately with Bowtie [14] and RSEM [15]. The metrics of TPM (Transcripts per million) and FPKM (Fragments Per Kilobase of exon per Million fragments mapped) were used. For each assembly, these data were: TPM mean: 23.46 and FPKM mean: 19.23; TPM median: 3.14 – FPKM median: 2.57 (Trinity) and TPM mean: 11.35 and FPKM mean: 47.75 – TPM median: 0.67; FPKM median: 2.82 (Newbler).

As expression measurements were not performed in replicate samples and because the samples were further enriched with eyestalk extracts, the measured expression values were relative and did not reflect the expression of the transcriptome at a specific time. The expression values were generally correlated between the two assemblies for similar transcripts (Table 2). The observed differences were related to the counting methods and could have been due to the large difference in the total number of transcripts (TPM) between the assemblies and/or the size of the transcripts (FPKM), with lower values obtained by the Trinity assembly since the transcripts were longer than in the Newbler assembly.

**Table 2:** Alphabetical list of peptide precursors, contig expression values and associated Blast matches.

Peptide designation	Size (aa)	Comp ID *Contig ID*	Size (pb)	TPM	FPKM	BLAST matches
Allatostatin	361	15895_c0_seq1	1444	5.01	4.11	Type A pre-pro-allatostatin (*Machrobrachium rosenbergii*)
A precursor		*06348*	*760*	*0.93*	*3.9*	4.4 e-51
Allatostatin	98	40876_c0_seq1	424	1.8	1.48	Type B pre-pro-allatostatin (*Pandalopsis japonica*)
B precursor		*-*	*-*	*-*	-	2.0 e-10
Allatostatin	106	7109_c0_seq1	589	11.08	9.08	Type C pre-pro-allatostatin (*Daphnia pulex*)
C precursor		*10325*	*501*	*2.12*	*8.92*	1.96 e-22
α Bursicon	148	25818_c0_seq1	636	3.51	2.88	Bursicon hormone alpha subunit (*Callinectes sapidus*)
precursor		*24449*	*273*	*0.58*	*2.42*	9.35 e-72
β Bursicon	137	17391_c0_seq1	701	4.1	3.36	Bursicon hormone beta subunit (*Homarus gammarus*)
precursor		*10676*	*489*	*0.8*	*3.38*	1.37 e-64
CCAP	140	20513_c0_seq1	607	4.71	3.86	Crustacean cardioactive peptide (*Procambarus clarkii*)
precursor		*20601*	*308*	*0.96*	*4.05*	7.16 e-32
CHH	131	4803_c0_seq2	493	21.75	17.83	CPRP/cHH precursor (*Charybdis japonica*)
precursor		*17526*	*347*	*3.57*	*15*	7.43 e-28
CLDH31	154	5480_c0_seq1	619	9.34	7.65	Pre-pro-calcitonin-like diuretic hormone (*Homarus*
precursor		*24873*	*269*	*1.33*	*5.6*	*americanus*) 6.16 e-23
CLDH33	149	5480_c0_seq2	598	11.06	9.06	Pre-pro-calcitonin-like diuretic hormone (*Homarus*
precursor		*17839*	*342*	*3.46*	*14.53*	*americanus*) 2.59 e-44
CLDH56	109	44788_c0_seq1	330	1.31	1.07	corticotropin releasing factor-like protein (*R. prolixus*)
precursor		-	-	-	-	7.61 e-7
Corazonin	92	814_c1_seq4	429	**176.02**	**144.28**	Pro-corazonin (*Acromyrmex echinatior*)
precursor 1		*24335*	*274*	***35.51***	***149.34***	4.66 e-7
Corazonin	82	814_c1_seq2	667	7.65	6.27	Corazonin preprohormone (*Daphnia pulex*)
precursor 2		*05623*	*841*	*1.94*	*8.18*	1.05 e-6
Eclosion	92	673_c2_seq1	1179	**121.27**	**99.4**	Eclosion hormone (*Amphibalanus amphitrite*)
hormone		*19560*	*320*	*6.82*	*28.68*	1.80 e-13
MIH/VIH1	82	46742_c0_seq1	423	1.39	1.14	Probable molt inhibiting hormone (*Jasus lalandii*)
		-	-	-	-	2.57 e-14
MIH/VIH2	48	-	-	-	-	Probable molt inhibiting hormone (*Jasus lalandii*)
		*17459*	*347*	*2.23*	*9.36*	1.93 e-8
Neuroparsin	103	18741_c0_seq1	382	5.96	4.88	Neuroparsin-A precursor (*Caligus rogercresseyi*)
1 precursor		*17227*	*351*	*1.16*	*4.86*	2.63 e-17
Neuroparsin	67	-	-	-	-	Putative neuroparsin (*Daphnia pulex*)
2 precursor		*16193*	*363*	*0.54*	*2.27*	7.97 e-7
NPF1	90	2304_c0_seq1	732	31.94	26.18	Preproneuropeptide F1 (*Litopenaeus vannamei*)
precursor		*31384*	227	*9.64*	*40.52*	1.00 e-28
NPF1L	128	2304_c0_seq2	734	10.68	8.75	Preproneuropeptide F2 (*Litopenaeus vannamei*)
precursor		-	-	-	-	2.27 e-47
NPF2	62	8750_c0_seq1	733	12.84	10.53	NPF-like precursor (*Nassonia vitripennis*)
precursor		*07726*	*643*	*2.24*	*9.41*	1.25 e-7
PDHLα	87	4981_c0_seq1	415	**23.89**	**19.58**	Pigment dispersing hormone (*Litopenaeus vannamei*)
precursor		*05401*	870	*2.65*	*11.15*	5.09 e-5
PDHLβ1	78	1192_c0_seq2	562	37.32	30.59	Pigment dispersing hormone (*Litopenaeus vannamei*)
precursor		*17681*	*344*	***30.05***	***58.16***	1.65 e-10
PDHLβ2	56	1192_c0_seq3	405	**53**	**43.44**	Pigment dispersing hormone (*Litopenaeus vannamei*)
precursor		*08747*	*576*	***15.73***	***66.14***	1.65 e-10
PDHβ	74	-	-	-	-	Pigment dispersing hormone type 2 (*Litopenaeus*
precursor		*33371*	*218*	***111.78***	***470.11***	*vannamei*) 1.54 e-15
RPCH	104	9506_c0_seq2	832	7.48	6.13	Red pigment concentrating hormone (*M. rosenbergii*)
precursor		*06590*	*737*	*1.33*	*5.61*	3.82 e-18
SIFamide	69	2588_c0_seq1	1015	18.97	15.55	SIFamide (*Procambarus clarkii*)
precursor		03910	*1128*	*3.92*	*16.5*	5.75 e-25
sNPF1	257	7638_c0_seq1	502	13.49	11.05	Short neuropeptide F precursor (*Aedes aegypti*)
precursor		*18326*	*335*	*2.35*	*9.89*	1.77 e-13
sNPF2	137	-	-	-	-	Short neuropeptide F precursor (*Aedes aegypti*)
precursor		*03109*	*791*	*2.19*	*9.21*	9.38 e-11
Tachykinin	205	4591_c0_seq3	1933	14.61	11.97	Preprotachykinin (*Panulirus interruptus*)
precursor		*03739*	*1170*	*4.47*	*18.8*	6.56 e-38

comp ID: sequences assembled with Trinity.*contig ID* (italic) sequences assembled with Newbler. Size (aa): deduced coding sequences. Size (pb): comp or *contig* sizes in pair bases. TPM  =  Transcripts Per Million. FPKM  =  Fragments Per Kilobase of exon per Million fragments mapped. Bold values are over the TPM or FPKM averages. Underlined peptides have been characterised by mass.

### Peptide families identified

Specific families of neuropeptide were analysed in this study, chosen on the basis of their putative physiological importance in insects and crustaceans. In this discussion, these peptide and peptidic hormone families have been grouped according to the existing entries in Pfam (http://pfam.sanger.ac.uk) or InterPro (http://www.ebi.ac.uk/interpro) and their arthropod specificity (Table 1).

### Generic peptide families

These peptides are present in a wide range of species and are not restricted to arthropods or crustaceans.

#### Corticotropin-Releasing Factor-Like Diuretic Hormone (CRFLDH) – PF00473/IPR000187

**Figure 1:**
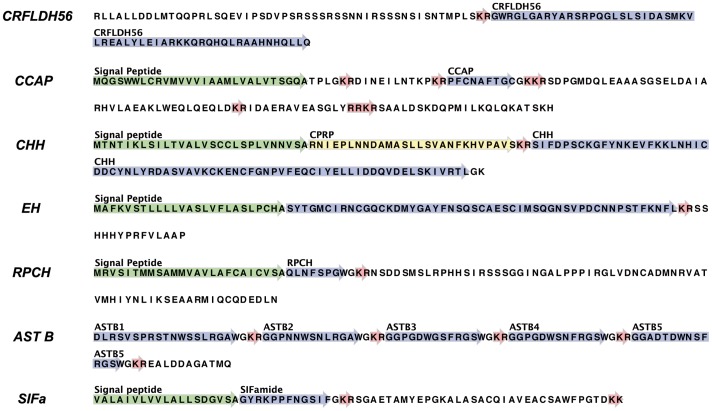
Complete and partial sequences from *E.* *crystallorophias* of the pre-pro-peptides containing the Corticotrophin Releasing Factor-like Diuretic hormone (CRFLDH), the Crustacean Cardio Active Peptide (CCAP), the Crustacean Hyperglycaemic Hormone (CHH), the Eclosion Hormone (EH), the Red Pigment Dispersing Hormone (RPCH), the Allatostatin Bs and the SIFamide. The mature peptides are highlighted in blue. The potential bibasic cleavage site is highlighted in red. The CPRP (CHH PRP) is highlighted in yellow.

Contig 44788 exhibited sequence similarity to the family of corticotropin releasing factors (CRFLDH) (Table 1; Figure 1). The precursor, although only a partial sequence, contained almost the complete CRFLDH. This peptide has been isolated from a number of insects [20]–[23], but has yet to be described in the Crustacea, except in the daphnia [7]. Sequence alignments with other gene family members from different species suggested that the final 2aa were absent in the *E. crystallorophias* orthologue (Figure S1 in File S1). However, this still resulted in a putative mature protein of 56aa (amino acids) (DH56) and therefore represented one of the longer sequences of this family along with that of *Ixodes* (DH61) [24]. The function of this gene in the Crustacea is unknown, but CRFLDH is associated with post-prandial diuresis in insects [25], [26].

#### Neuropeptide F (NPF/Y) -PF00159/IPR001955

**Figure 2:**
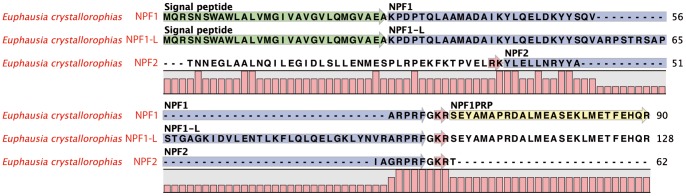
Complete protein sequences of the pre-pro-peptides of the Neuropeptides F1 and 2 from *E.* *crystallorophias*. The mature peptides are highlighted in blue only for *E. crystallorophias.* The signal peptides are highlighted in green and the potential bibasic cleavage sites, in red. The PRP highlighted in yellow was characterised by mass spectroscopy.

These peptides are between 28–45aa with a C-terminal motif of either -RPRFa or RVRFa. They are highly represented within the invertebrates, and in particular, the insects, but also a diverse array of arthropods including the crustaceans [6], [7], [17], [27]–[30]. In the *E. crystallorophias* assemblies, three precursors were identified (Table 1; Figure 2). The first of these encoded a putative 90aa protein, including a 29aa signal peptide. Euc-NPF1 was encoded immediately after the signal peptide and terminated at position 62 with a glycine, which permits amidation of the C-terminal with the production of a mature peptide of 32aa. The sequence corresponded to the consensus Xn-Y-L-X2-L-X2-Y-Y-X4-R-P-R-Fa proposed by Nässel and Wegener (2011) [31]. This sequence was identified in the eyestalk proteomic analysis. In addition, the sequence of the PRP, situated 3′ to Eu-NPF1 was also identified in the same analysis, thereby establishing that it is expressed. A sequence orthologous to this PRP also exists in the pro-peptide of the shrimp *Penaeus vannamei* (AEC12204, AEC12204) (Figure S2 in File S1) [30]. The second precursor represented the long form of the first transcript (NPF1-L) and apart from the extra insertion in NPF-1L, both transcripts were identical. NPF-1L appeared to be complete at a size of 70aa. It was interesting to note that the additional sequence is the same length in all species in which it has been identified to date (Figure S2 in File S1). Therefore it is difficult to determine whether it is indeed an alternative splice form or an incompletely processed intron [30]. However, this situation has previously been reported in the silk moth *Bombyx mori* [32]. The third transcript (NPF2), although only partial, was clearly different, especially in the N-terminus and contained a dibasic cleavage site upstream from the single tyrosine (Figure 2). This produced a short peptide of 17aa of YL/X5/YY/X2/AGRPRF. The sequence of this second form was validated by the discovery of one very similar in daphnia [7]. As with all other organisms, a single copy of NPF was present in the precursors. In contrast, the NPF characterised by a C-terminus of GX2RY and reported in the same cladoceran was not found in the transcriptome databases.

#### Tachykinin-Related Peptide (TKRP) – IPR013206

**Figure 3:**
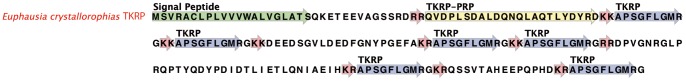
Complete protein sequence of the pre-pro-peptide containing the Tachykinins of *E. crystallorophias*. The 6 identical examples of Tachykinin are highlighted in blue. The signal peptide is highlighted in green and the potential bibasic cleavage sites, in red. The latter delimit the 4 potential PRPs. The PRP highlighted in yellow was characterised by mass spectrometry.

The tachykinins (or neurokinins) represent another group of highly conserved peptides. The transcript in *E. crystallorophias* contained a precursor of 618bp, putatively encoding a 206aa protein (Tables 1 and 2; Figure 3). 6 identical sequences of TKRP were present in the pro-peptide with the sequence APSGFLGMR-NH_2_. This sequence corresponds to that originally identified in the nervous system of the crab *Cancer borealis* [33] and has subsequently been identified in other arthropods [34]–[37] (Figure S3a in File S1). The precursor of TKRP in lobster is very similar with a virtually identical structure, but this species also has an extra isoform slightly modified at TPSGFLGMR-NH_2_ [37]. The pro-peptide possessed an extra 5 sequences potentially coding for PRPs. The mass spectrometry analysis, not only enabled the confirmation of Euc-TKRP (Figure S3b in File S1), but also one of the precursor-related peptides (Figure S3c in File S1).

### Peptide families specific to arthropods

These peptides are present in the Pfam and InterPro databases, but to date, have only been characterised in arthropods.

#### Allatostatin A (AST-A or -FGL amide) – PF05953/IPR010276

**Figure 4:**
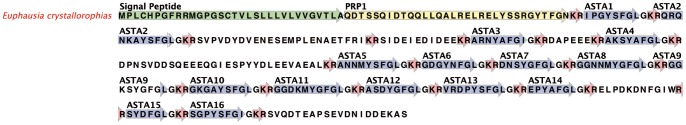
Complete protein sequence of the pre-pro-peptide containing the Allatostatin type As of d'*E.* *crystallorophias*. The 16 Allostatin As are highlighted in blue and numbered according to their position in the sequence. The signal peptide is highlighted in green and the potential bibasic cleavage sites, in red. The latter delimit the 8 potential PRPs. The PRP highlighted in yellow was characterised by mass spectrometry.

The members of this first family are characterised by a C-terminus with the structure: F/Y-X-F-G-L/I-amide. A single contig in the *E. crystallorophias* database potentially encoded the complete precursor molecule of AST-A, with a putative peptide sequence of 361aa and a signal peptide of 31 residues (Table 1; Figure 4). 16 sequences containing the signature of AST-A were present in the precursor, each with a size between 6–11 residues. This represents a greater number of copies than has been found previously in the insects (at 13–14 forms) [38], but fewer than in the majority of the crustaceans, where between 30 to more than 40 sequences have been characterised [39]–[41]. Each of the sequences appeared to be of a unique origin, which is in contrast to analyses in *Macrobrachium rosenbergi* [40] and *Procambarus clarkii* [39] where two AST-A sequences are present numerous times, indicating multiple duplication events. All of those identified in *E. crystallorophias* took the form: Y-X-F-G-L/I, with the final amino acid in the majority of cases (14 out of 16) being a leucine. The AST-A3 was the only one family member characterised in eyestalk extracts (Figure S4 in File S1) with a cryptic peptide (PRP1  =  Precursor-related peptide) localised immediately after the signal peptide and upstream of the pre-pro-peptide (Figure 4). These data, whilst hinting at the incomplete nature of the transcriptomes and mass spectrometry data, showed that allatostatin A is primarily produced in the eyestalks of *E. crystallorophias.*

#### Crustacean Cardioactive Peptide (CCAP)- PF11105/IPR024276

The predicted sequence of the pre-pro-peptide in *E. crystallorophias* was composed of 140aa with a signal peptide of 28aa (Figure 1). The CCAP sequence started at position 47 and finished at position 56 with a glycine, which is implicated in the amidation of the peptide. This sequence was identical to that found in all the decapods (Figure S5 in File S1). Four CCAP-PRPs were present in the propeptide, one upstream of CCAP and three downstream. Their function is unknown.

#### Crustacean Hyperglycemic Hormone (CHH) family- PF01147/IPR001166: Type I peptide: Crustacean Hyperglycemic Hormone *stricto sensu*

The nucleotide sequence produced from *E. crystallorophias* assembly covered 396bp putatively coding for the 131 aa of the pre-pro-peptide (Figure 1). This pre-pro-peptide could be partitioned into a signal peptide of 28aa, followed by a CPRP (CHH precursor-related peptide) of 27aa and finally a mature peptide of 72aa. The latter was obtained after the cleavage of the two terminal amino acids Gly(130)-Lys(131) during the C-terminal amidation of the peptide (Figure S6a in File S1).

The gene itself has alternative splice forms, producing either a short or a long form (CHH-L), of which increased length is the main characteristic [42]. Given the number of amino acids in the putative translation of the mature Euc-CHH sequence, it was clearly not the long form of the peptide. The potential for amidation at the C-terminal is an additional diagnostic since the long form never has this modification. This sequence therefore represented a CHH peptide as either the product of a gene of three exons, similar to that which exists in the peneid shrimps, or the spliced isoform of a CHH comprising 4 exons, the latter of which is composed of exons 1, 2 and 4.

The Euc-CPRP with a size of 27aa, was below the average size of these peptide inserts identified so far in Decapods, the longest being 50aa in the hermit crab *Pagurus bernhardus* [43] and was one of the smallest for a gene of 4 exons (Figure S6b in File S1). In effect, there appears to be a correlation between the structure of CPRP and the structure of the CHH gene of either 3 or 4 exons in the Malacostraca. A short CPRP (less than 20aa) is characteristic of a pro-peptide translated from a gene of 3 exons. Although significantly different from the sequences of the decapods, Euc-CPRP did contain the unique signatures for the propeptides found in genes of 4 exons (R_1_ – I_4_E_5_-L_7_ – M_11_-S_13_L_14_L_15_S_16_), in particular between amino acids 11 and 16. It should be noted that the RNA used in the sequencing was enriched using material from eyestalks to maximise the chances of identifying neurohormones from the X-organ-sinus gland complex. The RNAs coding for the long forms generally originate from different tissues (ganglions of the pericardial and thoracic ventral nervous system [42]), which were probably not sufficiently represented in the original extractions due to the low numbers of animals used and therefore were not identified in this study.

#### Crustacean Hyperglycemic Hormone (CHH) family- PF01147/IPR001166: Type II peptide: Vitellogenesis/Moult/Mandibular Organ Inhibiting Hormone

**Figure 5:**
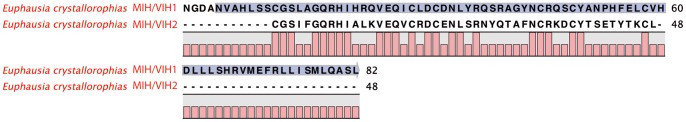
Alignment of the partial protein sequences of the pro-peptides of MIH/VIH from d'*E. crystallorophias*. The complete mature peptide of MIH/VIH1 is highlighted in blue. Only a partial sequence for MIH/VIH2 was identified.

In the *E. crystallorophias* transcriptome assembly, two partial sequences were identified with sequence similarity to type II peptides (Figure 5). A few publications have reported the presence of different isoforms of MIH/VIH, most notably in the peneid [44]–[48] and caridean shrimps [49], but not in any of the other decapods (Figure S7 in File S1). The sequence of the first form putatively encoded the complete mature peptide, comprising 78aa and the sequence signatures (Gly and Val) mentioned above. Although the signal peptide was incomplete, the sequence was similar to other type II family members, i.e. without an intermediate peptide, the mature peptide being produced directly from the signal peptide within the translated propeptide. The sequence of the second form was partial and comprised the amino acids between the first and last cysteine. There was a significant difference between the two forms, with only a sequence identity of 49% between the 47aa that both partial sequences had in common. This is in comparison with, for example, the 71% aa identity between isoforms in the peneid shrimp *Litopenaeus vannamei*. This large divergence at the sequence level implies a potential difference in function between the two forms, but this still has to be verified through functional studies.

#### Corazonin (CRZ)-IPR020190

**Figure 6:**
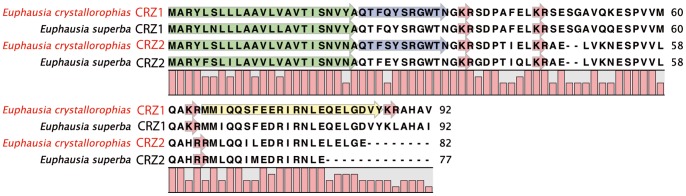
Alignment of the protein sequences of the pre-pro-peptides for Corazonin 1 and 2 from *E.* *crystallorophias* (completes) and *E. superba* (partial). The mature peptides are highlighted in blue only for *E. crystallorophias.* The signal peptides are highlighted in green and the potential bibasic cleavage sites, in red. The PRP highlighted in yellow was characterised by mass spectroscopy.

Corazonin is an 11aa peptide, blocked at both ends. The most common form of this peptide is: pQTFQYSRGWTNamide (Arg^7^-CRZ) [50],[51], In *E. crystallorophias* two different contigs coding for CRZ propeptides were identified (Table 1 and 2; Figure 6). The first pre-pro-Euc-CRZ1, comprised 92aa with a signal sequence estimated at 23 residues, cleavage of which could occur just upstream of the mature corazonin peptide (CRZ). This was identical to the form designated Arg^7^-CRZ, which has been identified in all other crustaceans (Figure S8a in File S1). This sequence was validated by the mass spectrometry data and confirmed the presence of a pyroGlu at the N-terminus and an amidation at the C-terminus (Figure S8b in File S1). Downstream of the CRZ1 pro-peptide were three further peptides (all essentially classified as Precursor Related Peptides (PRP's)) flanked with dibasic cleavage sites, the functions of which are unknown. The proteomic study enabled the characterisation of a third Euc-CRZ1-PRP in the eyestalks. However, whilst the CRZ peptides remain highly conserved between different taxa, the same is not true of the PRPs, which are extremely variable. To date, two PRPs have been described as being present in each pre-pro-protein of the insects [52]. An orthologue to the *E. crystallorophias* pre-pro*-*Euc-CRZ1 was identified in *E. superba*. However, the characterisation of the second form, pre-pro-Euc-CRZ2 produced a surprising result. It comprised 82aa with a signal peptide of a similar size to isoform 1, namely 23 residues. The downstream peptide was clearly a member of the corazonin family, but with a modification in position 4 with a serine replacing a glutamine (Ser^4^- Arg^7^-CRZ). Similarly to the pre-pro-EucCRZ1, the pre-pro-Euc-CRZ2 contained at least three other PRPs. The orthologue to the second isoform was also characterised in *E. superba*, but the Eus-CRZ2 was different to that in *E. crystallorophias*, with a glutamate in position 4 (Glu^4^- Arg^7^-CRZ). The number of CRZ-PRPs was identical. The existence of these two euphausiid orthologues of CRZ1 and 2 from different databases produced by different sequencing methods validated the existence of two isoforms in the same species. It is also worth noting that a second variant form exists in the daphnia (Gln^3^-Arg^4^-CRZ) (ACJ05606 and EFX86608).

It is thought that the primary functions of CRZ are involved in myotropism and pigmentotrophism. However a specific isoform: His^7^-CRZ has been identified and associated with the gregarious phase of locust [53]–[55]. This function is potentially very interesting with regard to krill, as these animals are found in large swarms at incredible densities.

#### Eclosion Hormone (EH) – IPR006825

This neuropeptide has been primarily characterised in insects where it is implicated with Ecdysis Triggering Hormone (ETH), CCAP and Bursicon in the hormonal cascade following cuticle hardening post-ecdysis [56], [57]. In contrast to the insects, this gene has been rarely described in the Eucrustacea. An EH-like transcript was present in the assembly of *E. crystallorophias*. The pro-peptide putatively comprised 92 residues with a signal peptide of 25aa (Table 1: Figure 1). The peptide of 67aa contained a dibasic cleavage site, which implied that the mature peptide was actually 51aa. This recognition site for cleavage enzymes seems to be present, not only in the euphausiids, but also in the peneids and the chelicerates (Figure S9 in File S1). In contrast, it is absent in most insects except *Tribolium castaneum*. The percentage of amino acid identity for the mature peptide varies from 25-39% and exceeds 49–76% when considering only the sequence 3′ to the first cysteine, at the dibasic cleavage site. There are 6 cysteines, which are position-invariant in all sequences.

#### Neuroparsin – PF07327/IPR010850

**Figure 7:**
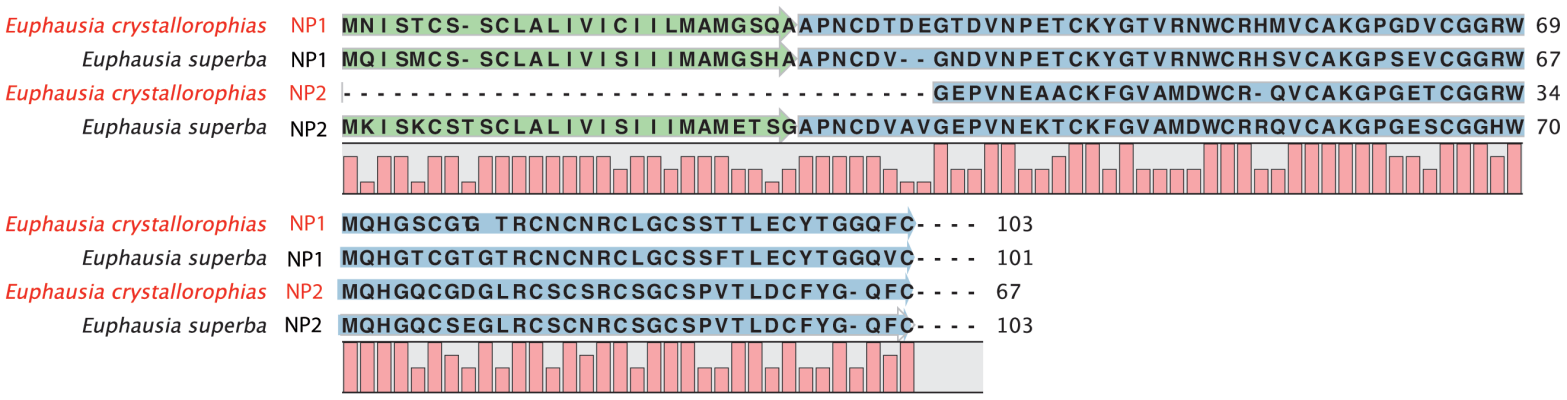
Complete protein sequences of the pre-pro-peptides of the Neuroparsins 1 and 2 of *E.* *crystallorophias* and of *E. superba*. The mature peptides are highlighted in blue. The signal peptides are highlighted in green.

In the *E. crystallorophias* assemblies, and also that of *E. superba*, two contigs with sequence similarity to neuroparsins were evaluated (Table 2; Figure 7). The precursors of the first isoform (NP1) comprised 103 aa and 101aa for each species respectively. The signal peptide was estimated to be 25aa for each of the two euphausiids. The propeptide of the second isoform (NP2) was incomplete in the N-terminal region for the *E. crystallorophias* transcript, but was full length in *E. superba* with 103aa and a signal peptide of 27 residues. Euc-NP1 possessed 15 cysteines as opposed to 14 for Eus-NP1, with 3 and 2 cysteines in the signal peptide respectively. Eus-NP2 comprised the same structure. It is worth noting that interrogating the assemblies using Blast sequence similarity searching revealed several different putative isoforms. Only the most highly represented isoforms, which were present in both euphausiid species were retained (Figure 7). Hence it is probable that there are more isoforms present than have been described here. The number of precursors is variable between taxons, although in the majority of cases, a single precursor has been characterised from sequence databases. Having said that the locust possesses four genes and the kissing bug *Rhodnius prolixus,* three (Figure S10 in File S1). In crustaceans, only a few ESTs have been characterised in decapods, a copepod and a cladoceran [7], [18], [19] (Figure S10 in File S1). A Blast search against the ESTs of *Penaeus monodon* revealed at least 3 isoforms of NP (GO071574, GW996622, GO080130).

#### Pigment Dispersing Hormones (PDH/PDF) – PF06324/IPR009396

**Figure 8:**
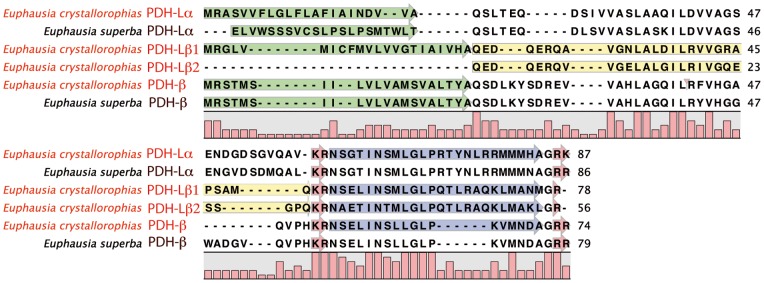
Complete protein sequences of the pre-pro-peptides of the Pigment Dispersing Hormones (PDH) α and β of *E.* *crystallorophias* and *E. superba*. The mature peptides are highlighted in blue only for *E. crystallorophias.* The signal peptides are highlighted in green and the potential bibasic cleavage sites, in red. The PRP highlighted in yellow was characterised by mass spectroscopy.

A multiple sequence alignment of this family of sequences in crustaceans shows that they possess two types (Figure S11a in File S1). These are designated α and β and share a conserved structure with a mature peptide of around 18aa [58]. A first partial sequence for *E. crystallorophias* and a full length sequence for *E. superba* [4] were retrieved from the databases (Table 1; Figure 8). This Euc/Eus-PDH was completely identical in amino acid sequence to other sequences in the decapod crustaceans and was the β form (Figure S11a in File S1). A second form was identified in *E. crystallorophias,* which was classified as a duplicated β form (PDH-Lβ1), because of the presence of a glutamate in position 3. However, whilst the first 12 amino acids of this peptide were conserved and confirmed membership of the PDHs, this sequence possessed a longer 3′ end, with a further 11aa in the mature peptide, not the usual 6 (Figure 8). The Euc-PDH-Lβ1 was also identified in the mass spectrometry studies validating it as an original form of PDH with a C-terminal amidation (Figure S11b in File S1). The same study showed that the PRP upstream of PDH-Lβ1 in the pro-peptide was also expressed, as suggested by its position between the signal peptide and a dibasic cleavage site. Its function is, of course, unknown. In addition, an extra form of PDH-L, which resembles a β form due to the presence of a glutamine at position 3, was identified in the assembly and was confirmed by proteomic analysis. This peptide (Euc-PDH-L β2) was distinguished by the presence of an alanine in place of the serine, classically located in position 2 of the mature peptide. The size of the putative peptide was 24aa, similar to the other long forms of PDH also identified in this study. The transcript was incomplete, but appeared to start at the PRP, with the traditional signal peptide absent. The PRP of this latter isoform was also identified in the proteomic analyses.

The assemblies revealed the presence of a further sequence with high sequence identity to PDH-L, but containing the sequence signature of type α; a glycine in position 3 (Figure 8). No “short” sequences of PDHα have been identified in euphausiid species. Extensive Blast sequence similarity searching revealed no further PDH-L gene family members outside euphausians and therefore the sequences described above would appear to be krill-specific. Clearly, with only sequence data available, the exact function of these transcripts cannot be determined, however a strong role in circadian rhythms might be predicted. Similar to RPCH (Red Pigment Concentrating Hormone) and AKH (Adiopokinetic Hormone), where the crustacean and insect orthologues have different names, PDH in crustacean is the orthologue of PDF (Pigment Dispensing Factor) in insects [59]–[61]. PDF has recently been implicated in the control of circadian rhythms of some crustaceans as well as in the insects [62], [63]. Krill undergo diurnal vertical migrations, living at depth during the day and returning to the surface waters at night to feed, which are influenced by seasonality. Hence there is an important requirement for krill, in particular *E. superba,* to have control over their daily rhythm, with an internal synchronicity and compartmentalisation in their physiology and localisation within the water column [64], [65]. This may be supplied by the putative PDH transcripts identified here, but clearly more work is required on these candidate genes. Multiple isoforms of this gene in krill suggests that there has been an expansion of functions in this species.

#### Red Pigment Concentrating Hormone (RPCH/AKH) – PF06377/IPR010475

In crustaceans, the variation in colour is controlled primarily by PDH, but also by RPCH. Similar to the situation with PDH, there are numerous orthologues to RPCH in the insects, which are called adipokinetic hormone (AKH) [66]. In the decapods, this sequence is highly conserved and consists of an octopeptide amidated at the C-terminal, p-QLNFSPGW-NH_2_. The putative *E. crystallorophias* RPCH pre-pro-peptide comprised 315bp with a signal peptide of 25aa, a mature peptide consisting of 8 characteristic amino acids and a C-terminal glycine which enabled amidation, upstream of which, there was a dibasic -KR- cleavage site (Figure 1). The sequence which was identified in the genomic database of *Daphnia pulex* appeared to be more related to AKH, rather than the RPCH of the decapods, due to a number of important modifications (3 out of the 8aa in positions 2, 6, and 7). This first sequence of RPCH from the (non-decapod) Malacostraca was identical to those described in the decapods (Figure S12a in File S1), but only one isoform was present in the krill assembly, similar to the situation in *Daphnia* where a single gene was identified [7]. This differed from the situation in insects where up to 3 different isoforms have been characterised in the locust, *Locusta migratoria* [67] (Figure S12b in File S1).

### Unreferenced peptide families

These were peptide families with no accession numbers or functional annotations in either Pfam or InterPro.

#### Allatostatin B (AST-B or-W(X6)Wamide)

Peptides belonging to the AST-B family have a tryptophan at the C-terminal and also at position 6. These peptides were primarily isolated in insects due to their myo-inhibitory effect and subsequently for their action on the prothoracic gland [68], [69]. The *E. crystallorophias* assembly contained several transcripts with sequence similarity to AST-B. The pre-pro-peptide sequence was not full-length, but 5 forms could be differentiated (Figure 1). Certain amino acids were conserved in all isoforms, in addition to the tryptophans previously mentioned. This permitted the development of a consensus sequence for the euphausiids: (G-G)-Xn-W-X3-RG-A/S-W-NH_2_. The two glycines in the C-terminus were present in 4 of the forms. There appeared to be fewer AST-B genes in *E. crystallorophias* than in other crustaceans, however this may simply be due to the partial nature of the transcriptome data. There are 13 of these sequences in *Carcinus maenas* [19], [70] and 9 in *Cancer borealis* [71]) where these peptides have been mainly characterised.

#### Allatostatin C (AST-C)

**Figure 9:**
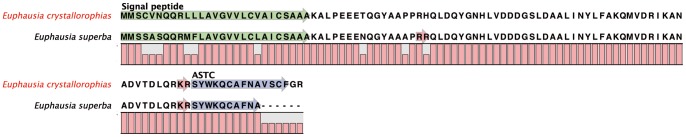
Protein sequence alignment of the pre-pro-peptides of the Allatostatin Cs of *E.* *crystallorophias* (complete) and *E. superba* (partial). The Allatostatin C is highlighted in blue. The signal peptide is highlighted in green and the potential bibasic cleavage sites, in red.

The type C allatostatins were originally described in *Manduca sexta* [72] and comprise a full-length peptide of 15 aa, with a non-amidated C-terminal with a common motif of -PISCF and a disulphide bridge between the cysteines at positions 7 and 14 [70]. These peptides have long been known in the Endopterygota (Holometabola) and have recently been described in the Crustacea [7], [18], [73]–[75]. A pre-pro-peptide of 106aa, with a signal sequence of 27 residues was present in the transcriptome assembly of *E. crystallorophias* (Figure 9). In common with previously characterised AST-C genes and in contrast to the A and B types, only a single copy of the mature peptide was present in the assembly. It was distinguished by the signature motif –AVSCF and possessed an identical sequence (SYWKQCAFNAVSCF) not only to those putatively identified in other crustaceans [73] but also to those in other species of insect (*Locusta migratoria*, *Apis melifera*, *Laupala kohalensis*): (Figure S13 in File S1). A partial sequence, which was highly similar to that identified in *E. crystallorophias,* was also found in the *E. superba* transcriptome. Despite reciprocal Blast searches, it was not possible to identify both isoforms in the same krill species. This is in line with the hypothesis of Dickinson et al, that each species only contains one form of this gene, which they proposed when they discovered a single peptide with the variant sequence –AVSCF in *H. americanus* [73]. However, more recently, an isoform with the –PISCF motif has since been discovered in the same species [76]. An identical situation (with 2 isoforms) has also been found in the crab *Cancer borealis* [77]. Therefore there is ample evidence to suggest that both forms are present, at least, within the decapods. To further underline the complexity found in this gene family, a study of the proteome of *Daphnia pulex,* not only demonstrated the presence of the two forms, but an additional one with the motif -AVSCF [7]. This last isoform was not present in either of the krill transcriptome assemblies.

#### Bursicon

**Figure 10:**
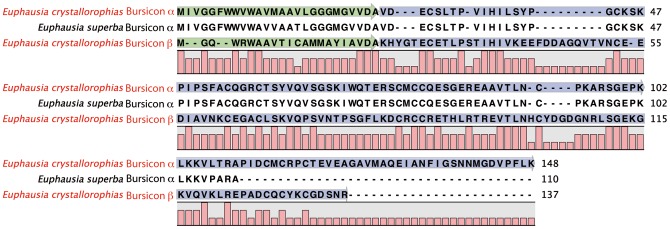
Protein sequence alignment of the pre-pro-peptides of Bursicon α and β of *E.* *crystallorophias* (complete) and Bursicon α of *E. superba* (partial). The mature peptides are highlighted in blue. The signal peptides are highlighted in green and the potential bibasic cleavage sites, in red. Only the sequences of *E. crystallorophias* are annotated.

Bursicon is a hormone well known in arthropods for its role in the hardening of the cuticle after moulting [78]–[80]. It is comprised of two sub-units, α and β which form a heterodimer [81]–[84]. Two distinct sequences were present in the assemblies of *E. crystallorophias* (Figure 10). The alignments with sequences from crustaceans and insects showed that the two sequences represented each one of the dimers, with 11 highly conserved and characteristic cysteines. The sequence of the bursicon α chain putatively comprised 146aa with a signal peptide of 26 residues. That of the β form was predicted at 137aa and included a signal peptide of 22 residues. Sequence conservation is important with each isoform, in particular for the sequences between the cysteines. The sequence of α in *E. crystallorophias* was practically identical to the orthologue in *E. superba* [18] and similar to the two daphnia species and also the other crustaceans, notably the decapods (Figure S14a in File S1). This conservation was also found in the β form (Figure S14b in File S1).

#### Calcitonin-Like Diuretic Hormones (CLDH)

**Figure 11:**
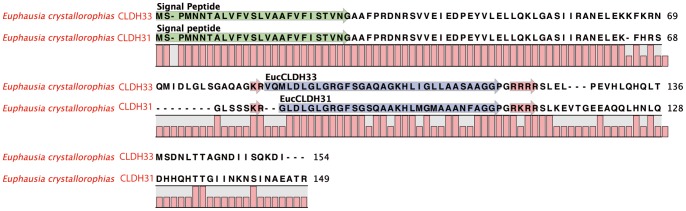
Alignment of the complete protein sequences of the pre-pro-peptides of the Calcitonin-like Diuretic hormones (CLDH 31 and 33) from *E.* *crystallorophias.* The mature peptides are highlighted in blue. The signal peptides are highlighted in green and the potential bibasic cleavage sites, in red.

Originally isolated from the nervous tissue of *Diploptera punctata* [85], CLDH or DH31 has only been characterised in three crustaceans *Homarus americanus* [86], *Daphnia pulex* [7], [74] and *Caligus clemensi* [18]. Similar to the situation in these three species, a contig with sequence similarity to DH31 was identified in *E. crystallorophias* (Table 1; Figure 11). It is possible to distinguish it from the DH31s of *Homarus* and *Tribolium* [22] by the exchange of L^22^ for M^22^, and *Acyrthosiphon* by the change of S^7^ to G^7^ [87]. The *E. crystallorophias* precursor was predicted to be 149aa long, encoding a signal peptide of 26aa, a PRP of 47aa separated from the mature peptide by a dibasic cleavage site and finally a second PRP of 38aa. Whilst DH31 is highly conserved within the different species of insect, crustaceans and chelicerates, the other parts of the precursor molecule are less well conserved (Figure S15 in File S1). The majority of CLDHs are 31aa, however, another form has been identified in the chelicerate *Ixodes scapularis*, which is extended by the addition of 3aa at the start of the sequence [24]. A second form with sequence similarity to CLDH with an extra 2aa was identified in *E. crystallorophias* (Figure S16 in File S1). If confirmed, this represents the first example of two CLDH isoforms within the same species. This second longer sequence was more variable than the first with 6aa substitutions in 31aa. The very high level of sequence conservation of this peptide within the Pancrustacea and to a similar extent in the insects suggests the action of a strong evolutionary selection pressure to maintain a major function, potentially that of a cardioactive peptide [86]. The number of important amino acid modifications found in the second contig could be explained by weaker selection pressure on this second copy, as a result of neofunctionalisation, enabling both gene copies to be retained in the genome.

#### SIFamide

The SIFamides represent a family of neuropeptides, which are highly conserved within the arthropods [89]. Since their first characterisation in the flesh fly *Neobelleria bullata* (as a myotropic agent) [90], an increasing number of these peptides have also been identified in the Hexapoda [91]–[94], in the Crustacea [7], [19], [34], [74], [88], [95]–[97] and in the Chelicerates [24]. They are highly abundant peptides and therefore relatively easy to isolate using techniques such as HPLC, MALDI and QTOF, but are also present in the molecular databases of genome sequences, ESTs and transcriptomes. The dodecapeptide contains the consensus sequence of the type XYRKPPFNGSIFamide where X^1^ can be R, G, V or T. Two exceptions exist for this 11aa structure (Figure S16 in File S1): *Daphnia pulex* where the Y^2^ is missing and the P^4^ is replaced with a leucine [7] and the aphid *Acyrthosiphon pisum* where the G^1^ seems to remain within the signal peptide and the mature peptide starting not with a tyrosine, but a phenylalanine [94]. It is interesting to note that the dibasic couplet within this sequence does not constitute a cleavage site [98] and to date the putative peptide PPFNGSIFamide has not been detected [91], [97]. Although there have been a limited number of precursor molecules identified, the structure is well defined. The SIFamide is positioned directly downstream of the signal peptide and upstream of a PRP. The function of the latter is unknown, but is characterised by the presence of 2 cysteines separated by 6 amino acids. The SIFamide has been implicated as a neuromodulator of reproduction [89], [91] and olfaction [39], [99].

The sequence of the putative precursor in the *E. crystallorophias* transcriptome (Figure 1) was truncated at the N-terminus and therefore the final pre-pro-peptide length of 77aa was missing 8aa from the signal peptide, the final sequence of which could therefore, not be confirmed. The Euc-SIFamide was present in full, as was the PRP. The mature peptide GYRKPPFNGSIFamide was identical to those in three other crustaceans *Cancer borealis, Carcinus maenas* and *Procambarus clarkii*, but also an insect, *Culex quinquefasciatus* (Figure S16 in File S1). This G^1^-SIFamide was the only such peptide identified in this analysis. Numerous isoforms have been identified in other crustaceans, such as *C. maenas* [19] and also *H. americanus* [97] in which a V^1^-SIFamide is present and was originally thought to be specific to lobsters. These two peptides have been identified in different tissues of the crab, with the isoform G^1^-SIFamide present within the brain, the sinus glands and the thoracic ganglions, whilst the second form V^1^-SIFamide is restricted to the thoracic ganglions. This regionalisation of expression could explain the varying success with which the different hormones have been isolated from krill. That the original RNA was enriched with extra eyestalks, which are a major source of the G^1^-SIFamide isoform, may have contributed to this finding.

#### Small Neuropeptide F

**Figure 12:**
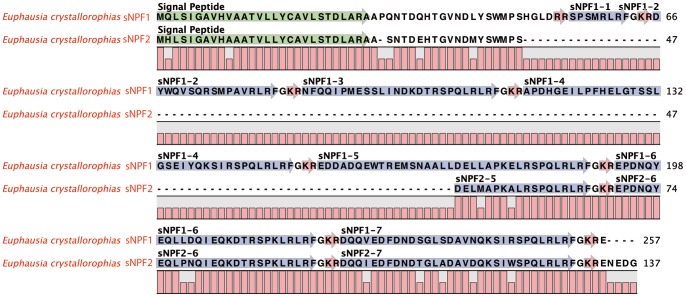
Complete protein sequences of the pre-pro-peptides of the small Neuropeptides F1 and 2 from *E.* *crystallorophias*. The mature peptides are highlighted in blue and numbered according to their position in the sequence. The signal peptides are highlighted in green and the potential bibasic cleavage sites, in red.

Contrary to what their name might imply the small neuropeptide F (sNPF) is not a short alternative spliced form of NPF as there are important differences in the precursors of the two types of peptides. There is considerable information on these peptides in crustaceans and insects. Although they are easy to characterise biochemically due to their small size, there is little information available on the structure of the pre-pro-peptides [31]. It seems that these peptides may be restricted to the arthropods, as they have yet to be identified in other taxa. Several isoforms are known in *Homarus americanus* (5) [88] and *Penaeus monodon* (4) [27]. In the *E. crystallorophias* transcriptome, two partial pre-pro-peptides were identified (Figure 12). The first, Euc-sPNF1putatively comprised 257aa, but there was no terminal stop codon. The signal peptide was 28aa. The characteristic C-terminus of Xn-P-X2-R-L-R-Fa was found in 7 peptides distinguished by dibasic cleavage sites. The size of each of these sNPFs was completely different, between 8–36aa. The second pre-pro-peptide Euc-sNPF2 was incomplete compared to the previous sequence, but aligned exactly with the N-terminal region (amino acids 1-48) and then less so up to position 172 of Euc-sNPF1. This precursor contained paralogues corresponding to sNPF1-5, 6 and 7, with peptide 5 truncated. In light of such results it was possible to hypothesize that two genes exist, coding for two different precursors, probably the result of a gene duplication, with, given the incomplete nature of the contigs, the potential to produce at least 14 different Euc-sNPFs. This number is larger than that reported for other species, but requires more verification from expression analyses. The sizes of some of these peptides were larger than those generally attributed to members of this peptide family. They reached the size range of those designated as NPFs and render the term “small” somewhat ineffective, especially since short forms of NFP were reported. To date, the existence of two genes has only been established in the mosquito *Aedes*, with a proposal for delineation into two families of peptides (head peptides versus sNPFs). However, it was not possible to make this distinction in the ice krill. The terminal structure of (L)RLRFa was remarkably conserved. Functional studies in *Drosophila* show that these peptides have functions in regulatory cascades controlling biological clock, feeding regulation, learning and memory [31].

### Levels of expression and characterisation by mass spectrometry

It was not possible to obtain an accurate overview of expression levels of each of the peptides identified in this study, due to the lack of replicates, but some clues could be provided by the relative expression levels in the assemblies and between the different techniques. The addition of RNA extracted from the eyestalks included in the RNA samples obtained from whole animals, prior to sequencing, meant that identification of peptides located to the X organ-sinus gland complex was reliant upon an over-representation of transcripts with regard to FPKM values. The proteomic analysis of the peptides obtained from eyestalk material confirmed both the presence of certain peptides predicted from the transcript assemblies and also their sequence. Neuropeptide transcripts with the highest FPKM values were also, for the most part, those which were characterised by mass spectrometry, indicating their importance in the initial mix of material. However, the reverse was not always true (Table 2).

The most highly represented peptides were those belonging to the PDH family, in particular PDHβ. This peptide possesses a particularly conserved structure within the decapod crustaceans and the importance of its expression and translation indicates one or more major biological functions. Although members of this family have been characterised in numerous species of arthropods, crustaceans and insects, the function, or functions, of these remain largely unknown and are potentially different between species: either neurohormone regulation of pigment movement and/or neurotransmission/modulation [100]. Given that there is clearly a structural relationship between PDH family members and those of PDF in insects, it is possible to infer that there might be homologous functions, in particular, in the modulation of circadian rhythms which are very important in krill. The most highly expressed members of PDH in other species are also PDHβ. Two isoforms exist, but these were not identified in krill [63], [101]–[105]. However, in *E. crystallorophias*, two long isoforms (PDHLβ I and II) co-exist with an α long form. These seem to be specific to the *Euphausiacea*, and are described for the first time in this study. Their expression levels were significantly lower than those for PDHβ [100], [105], [106].

If the peptides above, identified by the proteomic analyses, showed high expression levels, this was not necessarily the case in all peptides identified by the two techniques. Tachykinin and allatostatin A (represented by a single mature sequence (AST-A3) out of the 16 potential coding sequences) and their PRPs were characterised in both the eyestalk extracts and in the transcript assemblies. However, their FPKM values were much lower than those listed above (Table 2). This observation suggests that care has to be taken with the importance accorded to the relative expression levels of the PDHs. It is also interesting to note that the PRPs of the two precursor molecules were characterised by mass spectrometry, as was the case with those of PDHLβ, but not with PDHLα, however most abundant. Similarly, it was surprising that a single form of AST-A3 was characterised by mass spectrometry. It was clear that there was the potential for other forms to be expressed and translated due to the presence of dibasic cleavage sites in the precursor molecule, as evidenced by sequence data in the transcriptome. However, the lack of identification of these additional forms in the mass spectrometry data may have been for several reasons: the different extraction methods, the quality of the assemblies or the reality of very different translation levels for each of the AST-A transcripts. A potentially similar phenomenon is observed in *D. pulex* where out of 6 potential isoforms of AST-A, to date, only 4 have been confirmed by mass spectrometry [7].

CHH was the first peptide purified and sequenced from the eyestalks of the crab *Carcinus maenas*, largely as a result of its very high abundance [107]. It has not been characterised by mass spectrometry in this study due to the size filter that was applied (600–4,000Da), as its theorical mass is 8,389.5Da. The FPKM values of this peptide in this study, were lower than those of PDH and corazonin, underlining its lesser expression levels. It should be noted that CHH is functionally associated with stress, important for increasing the level of glucose in the circulation, by the glycolytic activation of glycogen stores in the midgut gland and the abdominal muscle [108]. The neuropeptide is primarily stored in the sinus gland and is released into the circulating haemolymph when stimulated. The quantity of RNA is therefore, not necessarily representative of the level of the available peptide and the expression is regulated via a feed-back mechanism [109].

In contrast, MIH/VIH1 was particularly weakly expressed, as evidenced by the low FPKM values. Most crustaceans stop or reduce the frequence of moulting and also their growth during their reproductive period, due to their respective energetic costs. In the krill, gravid females can moult and reproduce in parallel, which constitutes an adaptation to the very short productive period of the Antarctic summer [110]. The animals in this study were sampled in January and therefore were in their main period of maturation due to the very short window of opportunity for growth and reproduction in this region for phytoplankton feeders [111]. The weak expression levels of MIH/VIH1, which showed an inhibiting action in crustaceans tested to date, in either moulting or vitellogenesis, agree with the results here relating to the seasonality of the sampling period. The synergy between growth and moulting in the krill, does not allow for differentiation between the functions of VIH or MIH of the characterised peptide. It is also possible to consider the action of CHH on the secretion of moulting hormone and therefore its possible MIH function, as it has been demonstrated on the peneid shrimp *Penaeus vannamei* [112], which belongs to the decapods and is phylogenetically closest to the *Euphasiacea*. The possible functions of MIH/VIH2 are unknown.

Two other peptides were particularly represented in the assemblies: corazonin (CZN) and eclosion hormone (EH). The sequence of corazonin 1 was confirmed by mass spectrometry, and also had a high FPKM value in each assembly (Table 2). It was not possible to confirm the second isoform using this approach and the low FPKM value indicated a relatively low expression level in the eyestalks and potentially a different cellular origin. The two peptides are found in the insects and have been implicated in the regulation and development of the cuticle associated with moulting. They are upstream signalling molecules, activating the hormonal cascade responsible for initiating the moult. Corazonin is the most upstream molecule in this cascade, inducing the release of ecdysis triggering hormone (ETH), which was not identified in the assemblies. EH is expressed in response to ETH and is implicated in the pre-ecdysial phase. CCAP and bursicon are also implicated in the pre-ecdysis and post ecdysis phases respectively [57], [113]. The FPKM values for these last two peptides were lower than those for corazonin and EH. The mass spectrometry results for corazonin prove that corazonin is present in the eyestalks, as previously demonstrated in the lobster [88] and this localisation potentially explains its strong expression. The localisation of EH, is to date unknown in the crustaceans. This peptide was not identified in the mass spectrometry analyses, but the sequence of 118aa (12.9k Da) would exclude it from identification via this type of analysis on the basis of size (similarly to CHH and VIH/MIH) and this absence, therefore is not proof of absence in eyestalks. In contrast, its high expression level, without being definitive proof, supports the hypothesis of the site of production being in the eyestalks.

CCAP and bursicon, respectively, represent the subsequent molecules in the ecdysis cascade. They are not localised in the sinus gland of the eyestalks, but are known to be present in the same neuronal ganglions of the thorax and abdomen [114]–[116]. This simple difference explains their weak FPKM values. These two peptides are implicated in the final stages of moulting, as confirmed recently in the crab *Caninus maenas* [116], where, although they are the products of the same neurons in the pericardiac organ, they do not have the same expression kinetics, allied with important variations in the levels of CCAP during the moult cycle. This variant in expression was not observed for bursicon. The links in the chain controlling the moult cycle in krill were therefore clearly present, similar to most of the arthropods, in significant quantities, but their different localisation does not allow a full interpretation with regard to relating their variation in expression levels with the moult cycle.

## Conclusions

Although the peptides of arthropods have been the subject of numerous studies, including transcriptomic approaches [7], [17], [117], [118], these have been largely focussed on hexapods and decapod crustaceans. Besides the interest in krill, as polar sentinels for climate change, these eucarids are also at the base of the decapod lineage [119] and therefore represent an important taxa for evolutionary studies. This study presents, for the first time an overview of neuropeptides in euphasiids. These have been principally described in *E. crystallorophias,* but also to a lesser extent in *E. superba*, some of which, to date, have only been characterised in a handful of arthropods. This study characterised new mature peptide sequences in krill (61 and 8, from *E. crystallorophias* and *E. superba* respectively) including, in most cases, the encoding pre-pro-peptide sequence.

In this paper, we describe neuropeptides belonging to several gene families encoded by 36 different precursor molecules. Within the *E. crystallorophias* peptides, 6 were confirmed by mass spectrometry on eyestalk extracts along with 6 precursor-related-peptides (Table 1). Numerous other peptides obtained by mass spectrometry were also identified in the transcriptome assemblies. However, when these sequences were searched against the databases, the matches retrieved classified them as “unknown”, even if the orthologues were found in arthropods with a complete genome sequence. Therefore, the description of this group of neuropeptides is almost certainly not exhaustive and underestimates the information available within both the sequence and protein data.

The transcriptomes in this study were produced from two sister species of krill, the ice krill and the Antarctic krill using two types of Next Generation Sequencing (454 for *E. Superba* and Illumina for *E. crystallorophias*) and analysed via two different assembly packages (Newbler and Trinity). The use of these two assemblers aided sequence validation, as did the comparative approach of using two species, which aided delineation of transcript structure, as there were notable sequence differences to those previously identified in other crustaceans or hexapods. The prime example of this was the characterisation of PDH-L, which to date has not been identified in any other species. Additionally, the presence of the long forms of PDHα in the proteome provided final proof of their translation and underlined the diversity of PDH isoforms and their probable physiological diversity.

The proteomic analysis carried out on the extracts of eyestalks of *E. crystallorophias* provided a final confirmation of sequence. This analysis also proved, as in the example of PDH-Lα, the existence of biologically active peptides (PRPs: Pro-peptide-Related Peptides), which were previously only predicted, based on the sequence of the pro-peptides. These data attest to the high level of expression and translation of mRNAs coding for these pre-pro-peptides in eyestalks as seen in the pro-peptides of allatostatin A, Arg^7^-corazonin 1, neuropeptide F1, PDH-Lβ1and 2, and tachykinin.

It was not surprising that most of the neuropeptides identified in *E. crystallorophias* were identified by sequence database searching, as all the peptides in this group are remarkably conserved. This included the PRPs present in certain pro-peptides indicating a strong selection pressure, at least on one of the paralogues, even if multiple copies of the gene were encoded in the genome. The sequences described here, were often highly similar to those of peneid shrimps, confirming phylogenetic studies which show them to share a common ancestor with the decapods [119].

The functionality of the peptides characterised in this study, must remain speculative, as these data are reliant upon predictions from studies carried out in neighbouring taxa, such as the hexapods or decapods. However, the sequences reported here have been identified as key molecules in the life history traits of a wide range of species, including the Crustacea. Hence a detailed understanding of these hormones will provide valuable biomarkers for studies into the effects of climate change and the potential trade-offs that occur between life history traits in a changing world. They will also enable more in-depth studies into the molecular evolution and phylogeny of krill.

## Supporting Information

File S1 **File includes Figures S1–S12.** Figure S1. Alignment of the peptide sequences of the pre-pro-peptides of Corticotropin Releasing Factor Like Diuretic Hormone (CRFLDH) identified in the Pancrustacea. The mature peptides are highlighted in blue only for *E. crystallorophias.* The signal peptides are highlighted in green and the potential bibasic cleavage sites, in red. The histogram at the base of the figure indicates the level of conservation of each amino acid in the sequences. Figure S2. Alignment of the peptide sequences of the pre-pro-peptides of The Neuropeptide Fs (NPF) identified in the Pancrustacea. The mature peptides are highlighted in blue only for *E. crystallorophias.* The signal peptides are highlighted in green and the potential bibasic cleavage sites, in red. The PRP highlighted in yellow was characterised by mass spectrometry during the course of this study. The histogram at the base of the figure indicates the level of conservation of each amino acid in the sequences. Figure S3. Alignment and Maldi TOF MS/MS spectra of the Tachykinin Related Peptides. Figure S3a: Alignment of the peptide sequences of the pre-pro-peptides of the Tachykinin Related Peptides (TKRP) identified in the Pancrustacea. The mature peptides are highlighted in blue. The signal peptides are highlighted in green and the potential bibasic cleavage sites, in red. The PRP highlighted in yellow was characterised by mass spectrometry during the course of this study. The histogram at the base of the figure indicates the level of conservation of each amino acid in the sequences. Figure S3b: Maldi TOF MS/MS spectra of MH+ APSGFLGMRa (Euc-TKRP); Figure S3c: Maldi TOF MS/MS spectra of MH+ pQVDPLSDALDQNQLAQTLYDYRD (Euc-TKRP-PRP). Figure S4. Maldi TOF MS/MS spectra of MH+ ARNYAFGIa (Euc-AST A3). Figure S5. Alignment of the peptide sequences from the pre-pro-peptides of the crustacean cardioactive peptide (CCAP) identified in the Eucrustacea. The mature peptide highlighted in blue belongs to *E. crystallorophias.* The signal peptides are highlighted in green and the potential dibasic cleavage sites in red. The histogram at the base of the figure indicates the level of conservation of each amino acid in the sequences. Figure S6. Alignments of the CHH and CHH precursor-related peptides (CPRP) identified in Eucrustacea. Figure S6a: Alignment of the CHH peptide sequences identified in the Eucrustacea. The mature peptide highlighted in blue belongs to *E. crystallorophias.* The six characteristic cysteines of the peptide are highlighted in yellow in this study. The histogram at the base of the figure indicates the level of conservation of each amino acid in the sequences. Figure S6b: Alignment of the peptide sequences of the CHH precursor-related peptides (CPRP) identified in the Eucrustacea. The CPRP highlighted in yellow belongs to *E. crystallorophias.* The histogram at the base of the figure indicates the level of conservation of each amino acid in the sequences. Figure S7. Alignment of the peptide sequences of VIH/MIH identified in the Eucrustacea. The mature peptide highlighted in blue belongs to *E. crystallorophias.* The six characteristic cysteines are highlighted in yellow. The glycine highlighted in red is equally characteristic of members of the CHH type II peptides. The histogram at the base of the figure indicates the level of conservation of each amino acid in the sequences. Figure S8. Alignment and Maldi TOF MS/MS spectra of Euc-Corazonins. Figure S8a: Alignment of the peptide sequences of the pre-pro-peptides of Corazonin (CRZ) identified in the Arthropods. The mature peptides are highlighted in blue only for *E. crystallorophias.* The signal peptides are highlighted in green and the potential bibasic cleavage sites, in red. The PRP highlighted in yellow was characterised by mass spectroscopy in this study. The histogram at the base of the figure indicates the level of conservation of each amino acid in the sequences. Figure S8b: Maldi TOF MS/MS spectra of MH+ pQTFQYSRGWTNa (Euc-Arg^7^-CRZ1). Figure S9. Alignment of the peptide sequences of the pre-pro-peptides of the Eclosion Hormone (EH) identified in the Arthropods. The mature peptides are highlighted in blue only for *E. crystallorophias.* The signal peptides are highlighted in green and the potential bibasic cleavage sites, in red. The histogram at the base of the figure indicates the level of conservation of each amino acid in the sequences. Figure S10. Alignment of the peptide sequences of the pre-pro-peptides of the Neuroparsins (NP) identified in the Pancustacea. The mature peptides are highlighted in blue only for *E. crystallorophias.* The signal peptides are highlighted in green and the potential bibasic cleavage sites, in red. The histogram at the base of the figure indicates the level of conservation of each amino acid in the sequences. Figure S11. Alignment and Maldi TOF MS/MS spectra of PDH. Figure S11a: Alignment of the peptide sequences of the pre-pro-peptides of the Pigment Dispersing Hormones (PDH) and Pigment Dispersing Factors identified in the Pancrustacea. The mature peptides are highlighted in blue only for *E. crystallorophias.* The signal peptides are highlighted in green and the potential bibasic cleavage sites, in red. The PRP highlighted in yellow was characterised by mass spectrometry during the course of this study. The histogram at the base of the figure indicates the level of conservation of each amino acid in the sequences. Figure S11b: Maldi TOF MS/MS spectra of MH+NSELINSMLGLPQTLRAQKLMANMa (Euc-PDH-L β1). Figure S12. Alignments of the peptide sequences of Red Pigment Concentrating Hormones (RPCH) identified in the Eucrustacea and Pancrustacea. Figure S12a: Alignment of the peptide sequences of the pre-pro-peptides of Red Pigment Concentrating Hormones (RPCH) identified in the Eucrustacea. The mature peptides are highlighted in blue only for *E. crystallorophias.* The signal peptides are highlighted in green and the potential bibasic cleavage sites, in red. The histogram at the base of the figure indicates the level of conservation of each amino acid in the sequences. Figure S12b: Alignment of the peptide sequences of the Red Pigment Concentrating Hormones (RPCH) and AKH identified in the Pancrustacea. The mature peptides highlighted in blue belong to the Eucrustacea. The histogram at the base of the figure indicates the level of conservation of each amino acid in the sequences. Figure S13. Alignment of the mature peptide sequences of the Allatostatin Cs identified in arthropods. The mature peptide highlighted in blue belongs to *E. crystallorophias.* The histogram at the base of the figure indicates the level of conservation of each amino acid in the sequences. Figure S14. Alignment of the peptide sequences of the pre-pro-peptides of Bursicon α and β identified in the Eucrustacea. Figure S14a: Alignment of the peptide sequences of the pre-pro-peptides of Bursicon α identified in the Eucrustacea. The mature peptide highlighted in blue belongs to *E. crystallorophias.* The histogram at the base of the figure indicates the level of conservation of each amino acid in the sequences. Figure S14b: Alignment of the peptide sequences of the pre-pro-peptides of Bursicon β identified in the Eucrustacea. The mature peptide highlighted in blue belongs to *E. crystallorophias.* The histogram at the base of the figure indicates the level of conservation of each amino acid in the sequences. Figure S15. Alignment of the peptide sequences of the pre-pro-peptides of Calcitonin-like Diuretic Hormone (CLDH) identified in the Arthropods. The mature peptides are highlighted in blue only for *E. crystallorophias.* The signal peptides are highlighted in green and the potential bibasic cleavage sites, in red. The PRP highlighted in yellow was characterised by mass spectroscopy in this study. The histogram at the base of the figure indicates the level of conservation of each amino acid in the sequences. Figure S16. Alignment of the peptide sequences of the pre-pro-peptides of SIFamides identifed in the Arthropods. The mature peptides are highlighted in blue only for *E. crystallorophias.* The signal peptides are highlighted in green and the potential bibasic cleavage sites, in red. The histogram at the base of the figure indicates the level of conservation of each amino acid in the sequences.
(PDF)
