# Supplementary material for: Transcriptome and Peptidome Characterisation of the Main Neuropeptides and Peptidic Hormones of a Euphausiid: The Ice Krill, Euphausia crystallorophias
Source: PLoS One. 2013 Aug 21;8(8):e71609. doi: 10.1371/journal.pone.0071609 (PMC3749230; doi:10.1371/journal.pone.0071609)
Supplement: File S1 — File includes Figures S1–S12. Figure S1. Alignment of the peptide sequences of the pre-pro-peptides of Corticotropin Releasing Factor Like Diuretic Hormone (CRFLDH) identified in the Pancrustacea. The mature peptides are highlighted in blue only for E. crystallorophias. The signal peptides are highlighted in green and the potential bibasic cleavage sites, in red. The histogram at the base of the figure indicates the level of conservation of each amino acid in the sequences. Figure S2. Alignment of the peptide sequences of the pre-pro-peptides of The Neuropeptide Fs (NPF) identified in the Pancrustacea. The mature peptides are highlighted in blue only for E. crystallorophias. The signal peptides are highlighted in green and the potential bibasic cleavage sites, in red. The PRP highlighted in yellow was characterised by mass spectrometry during the course of this study. The histogram at the base of the figure indicates the level of conservation of each amino acid in the sequences. Figure S3. Alignment and Maldi TOF MS/MS spectra of the Tachykinin Related Peptides. Figure S3a: Alignment of the peptide sequences of the pre-pro-peptides of the Tachykinin Related Peptides (TKRP) identified in the Pancrustacea. The mature peptides are highlighted in blue. The signal peptides are highlighted in green and the potential bibasic cleavage sites, in red. The PRP highlighted in yellow was characterised by mass spectrometry during the course of this study. The histogram at the base of the figure indicates the level of conservation of each amino acid in the sequences. Figure S3b: Maldi TOF MS/MS spectra of MH+ APSGFLGMRa (Euc-TKRP); Figure S3c: Maldi TOF MS/MS spectra of MH+ pQVDPLSDALDQNQLAQTLYDYRD (Euc-TKRP-PRP). Figure S4. Maldi TOF MS/MS spectra of MH+ ARNYAFGIa (Euc-AST A3). Figure S5. Alignment of the peptide sequences from the pre-pro-peptides of the crustacean cardioactive peptide (CCAP) identified in the Eucrustacea. The mature peptide highlighted in blue belongs to E. cry [file pone.0071609.s001.pdf]

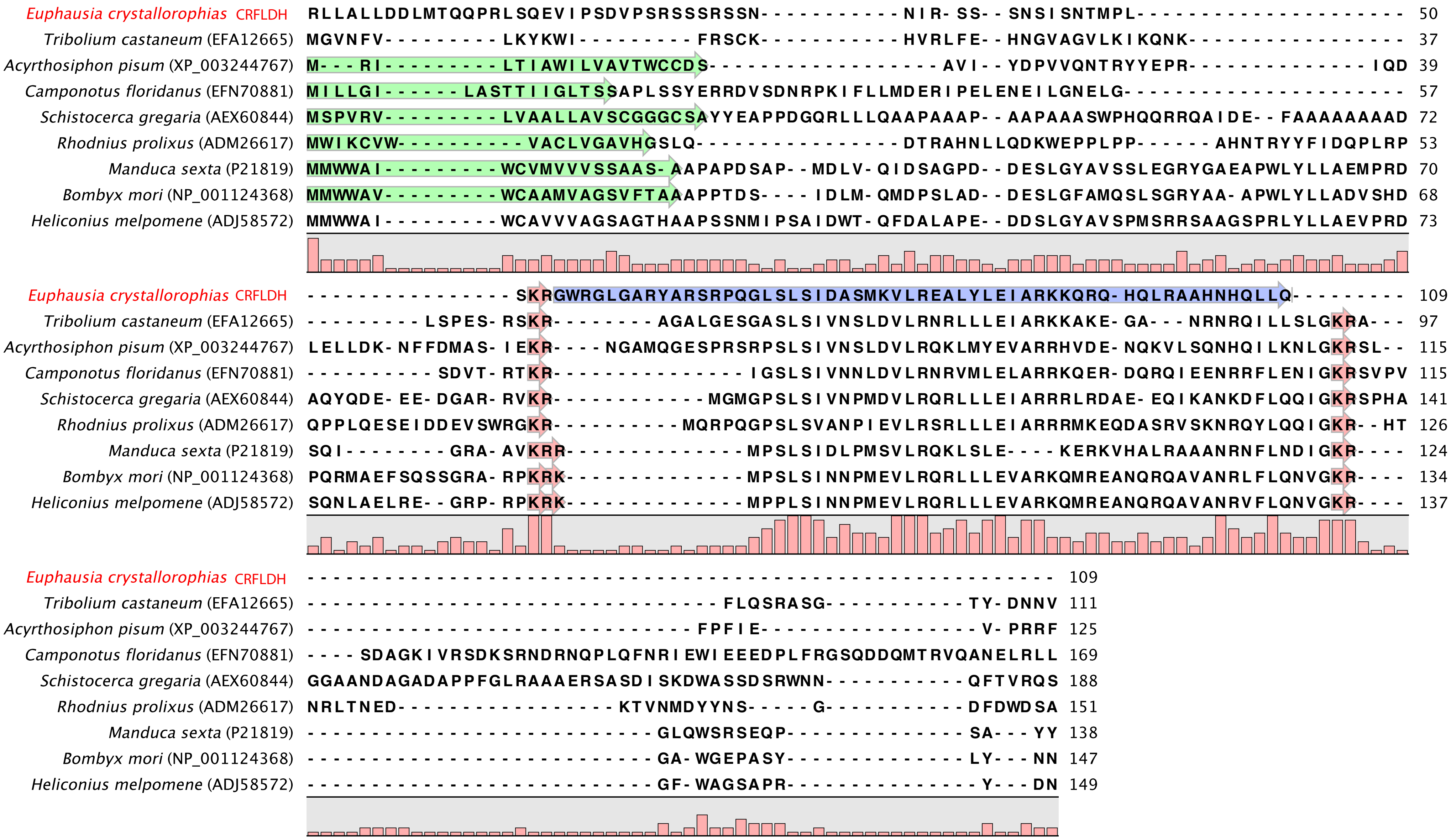
Euphausia crystallorophias CRFLDH RLLALLDDLMTQQPRLSQEVIPSDVPSRSSSRSSN-----------NIR-SS--SNSISNTMPL--------------------- 50
Tribolium castaneum (EFA12665) MGVNFV---------LKYKWI---------FRSCK-----------HVRLFE--HNGVAGVLKIKQNK----------------- 37
Acyrthosiphon pisum (XP_003244767) M---RI---------LTIAWILVAVTWCCDS------------------AVI--YDPVVQNTRYYEPR--------------IQD 39
Camponotus floridanus (EFN70881) MILLGI------LASTTIIGLTSSAPLSSYERRDVSDNRPKIFLLMDERIPELENEILGNELG---------------------- 57
Schistocerca gregaria (AEX60844) MSPVRV---------LVAALLAVSCGGGCSAYYEAPPDGQRLLLQAAPAAAP--AAPAAASWPHQQRRQAIDE--FAAAAAAAAD 72
Rhodnius prolixus (ADM26617) MWIKCVW----------VACLVGAVHGSLQ----------------DTRAHNLLQDKWEPPLPP------AHNTRYYFIDQPLRP 53
Manduca sexta (P21819) MMWWAI---------WCVMVVVSSAAS-AAPAPDSAP--MDLV-QIDSAGPD--DESLGYAVSSLEGRYGAEAPWLYLLAEMPRD 70
Bombyx mori (NP_001124368) MMWWAV---------WCAAMVAGSVFTAAAPPTDS----IDLM-QMDPSLAD--DESLGFAMQSLSGRYAA-APWLYLLADVSHD 68
Heliconius melpomene (ADJ58572) MMWWAI---------WCAVVVAGSAGTHAAPSSNMIPSAIDWT-QFDALAPE--DDSLGYAVSPMSRRSAAGSPRLYLLAEVPRD 73
Euphausia crystallorophias CRFLDH ----------------SKRGWRGLGARYARSRPQGLSLSIDASMKVLREALYLEIARKKQRQ-HQLRAAHNHQLLQ--------- 109
Tribolium castaneum (EFA12665) ---------LSPES-RSKR--------AGALGESGASLSIVNSLDVLRNRLLLEIARKKAKE-GA---NRNRQILLSLGKRA--- 97
Acyrthosiphon pisum (XP_003244767) LELLDK-NFFDMAS-IEKR----NGAMQGESPRSRPSLSIVNSLDVLRQKLMYEVARRHVDE-NQKVLSQNHQILKNLGKRSL-- 115
Camponotus floridanus (EFN70881) ----------SDVT-RTKR---------------IGSLSIVNNLDVLRNRVMLELARRKQER-DQRQIEENRRFLENIGKRSVPV 115
Schistocerca gregaria (AEX60844) AQYQDE-EE-DGAR-RVKR------------MGMGPSLSIVNPMDVLRQRLLLEIARRRLRDAE-EQIKANKDFLQQIGKRSPHA 141
Rhodnius prolixus (ADM26617) QPPLQESEIDDEVSWRGKR----------MQRPQGPSLSVANPIEVLRSRLLLEIARRRMKEQDASRVSKNRQYLQQIGKR--HT 126
Manduca sexta (P21819) SQI--------GRA-AVKRR--------------MPSLSIDLPMSVLRQKLSLE----KERKVHALRAAANRNFLNDIGKR---- 124
Bombyx mori (NP_001124368) PQRMAEFSQSSGRA-RPKRK--------------MPSLSINNPMEVLRQRLLLEVARKQMREANQRQAVANRLFLQNVGKR---- 134
Heliconius melpomene (ADJ58572) SQNLAELRE--GRP-RPKRK--------------MPPLSINNPMEVLRQRLLLEVARKQMREANQRQAVANRVFLQNVGKR---- 137
Euphausia crystallorophias CRFLDH -- 109
Tribolium castaneum (EFA12665) --------------------------------FLQSRASG-----------TY-DNNV 111
Acyrthosiphon pisum (XP_003244767) ------------------------------FPFIE-----------------V-PRRF 125
Camponotus floridanus (EFN70881) ----SDAGKIVRSDKSRNDRNQPLQFNRIEWIEEEDPLFRGSQDDQMTRVQANELRLL 169
Schistocerca gregaria (AEX60844) GGAANDAGADAPPFGLRAAAERSASDISKDWASSDSRWNN-----------QFTVRQS 188
Rhodnius prolixus (ADM26617) NRLTNED-----------------KTVNMDYYNS-----G-----------DFDWDSA 151
Manduca sexta (P21819) ---------------------------GLQWSRSEQP--------------SA---YY 138
Bombyx mori (NP_001124368) ---------------------------GA-WGEPASY--------------LY---NN 147
Heliconius melpomene (ADJ58572) ---------------------------GF-WAGSAPR---------------Y---DN 149

Figure S1

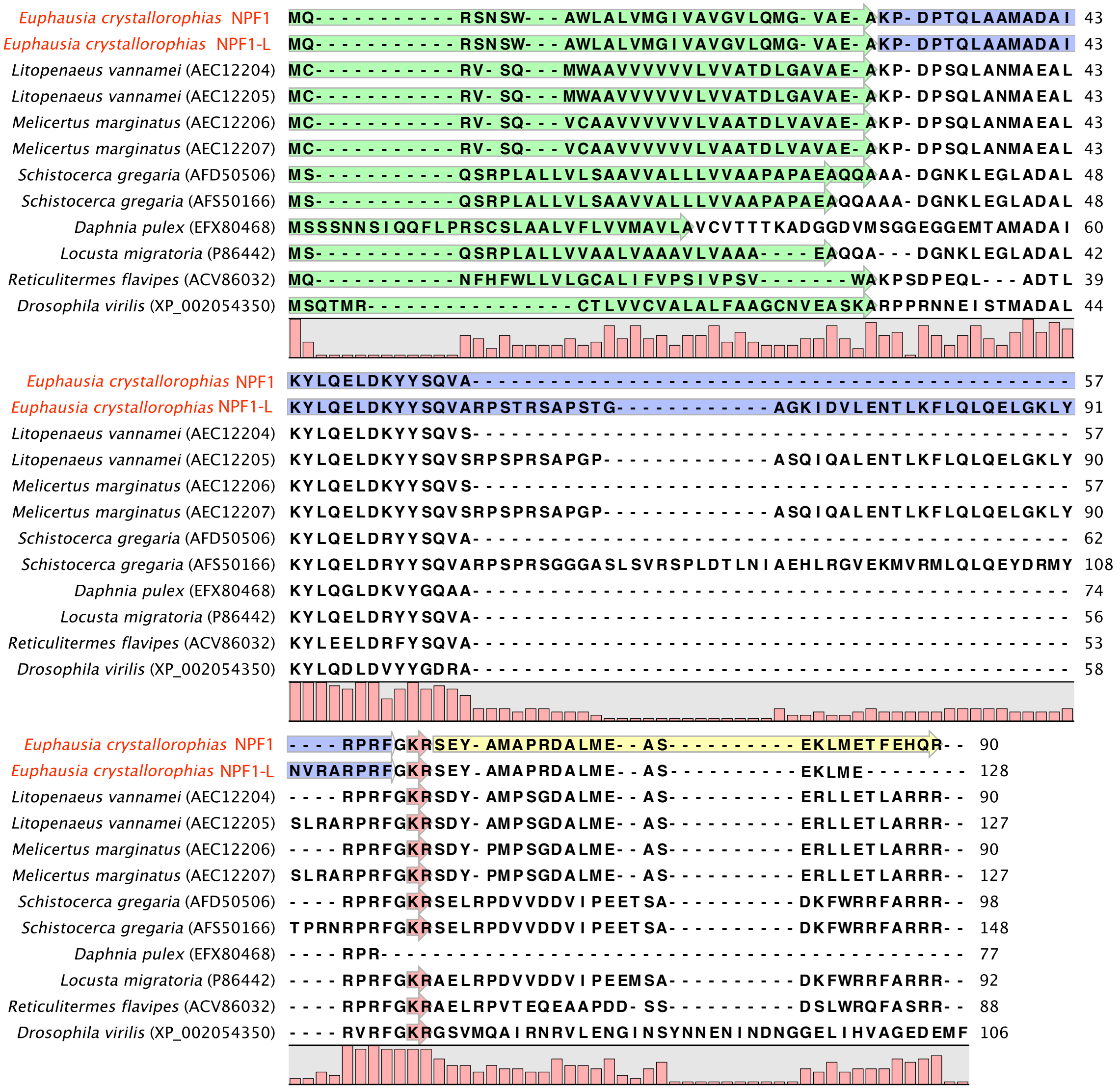
Euphausia crystallorophias NPF1 MQ-----------RSNSW---AWLALVMGIVAVGVLQMG-VAE-AKP-DPTQLAAMADAI 43
Euphausia crystallorophias NPF1-L MQ-----------RSNSW---AWLALVMGIVAVGVLQMG-VAE-AKP-DPTQLAAMADAI 43
Litopenaeus vannamei (AEC12204) MC-----------RV-SQ---MWAAVVVVVVLVVATDLGAVAE-AKP-DPSQLANMAEAL 43
Litopenaeus vannamei (AEC12205) MC-----------RV-SQ---MWAAVVVVVVLVVATDLGAVAE-AKP-DPSQLANMAEAL 43
Melicertus marginatus (AEC12206) MC-----------RV-SQ---VCAAVVVVVVLVAATDLVAVAE-AKP-DPSQLANMAEAL 43
Melicertus marginatus (AEC12207) MC-----------RV-SQ---VCAAVVVVVVLVAATDLVAVAE-AKP-DPSQLANMAEAL 43
Schistocerca gregaria (AFD50506) MS-----------QSRPLALLVLSAAVVALLLVVAAPAPAEAQQAAA-DGNKLEGLADAL 48
Schistocerca gregaria (AFS50166) MS-----------QSRPLALLVLSAAVVALLLVVAAPAPAEAQQAAA-DGNKLEGLADAL 48
Daphnia pulex (EFX80468) MSSSNNSIQQFLPRSCSLAALVFLVVMAVLAVCVTTTKADGGDVMSGGEGGEMTAMADAI 60
Locusta migratoria (P86442) MS-----------QSRPLALLVVAALVAAAVLVAAA----EAQQA---DGNKLEGLADAL 42
Reticulitermes flavipes (ACV86032) MQ-----------NFHFWLLVLGCALIFVPSIVPSV-------WAKPSDPEQL---ADTL 39
Drosophila virilis (XP_002054350) MSQTMR----------------CTLVVCVALALFAAGCNVEASKARPPRNNEISTMADAL 44

Euphausia crystallorophias NPF1 KYLQELDKYYSQVA--- 57
Euphausia crystallorophias NPF1-L KYLQELDKYYSQVARPSTRSAPSTG------------AGKIDVLENTLKFLQLQELGKLY 91
Litopenaeus vannamei (AEC12204) KYLQELDKYYSQVS--- 57
Litopenaeus vannamei (AEC12205) KYLQELDKYYSQVSRPSPRSAPGP-------------ASQIQALENTLKFLQLQELGKLY 90
Melicertus marginatus (AEC12206) KYLQELDKYYSQVS--- 57
Melicertus marginatus (AEC12207) KYLQELDKYYSQVSRPSPRSAPGP-------------ASQIQALENTLKFLQLQELGKLY 90
Schistocerca gregaria (AFD50506) KYLQELDRYYSQVA--- 62
Schistocerca gregaria (AFS50166) KYLQELDRYYSQVARPSPRSGGGASLSVRSPLDTLNIAEHLRGVEKMVRMLQLQEYDRMY 108
Daphnia pulex (EFX80468) KYLQGLDKVYGQAA--- 74
Locusta migratoria (P86442) KYLQELDRYYSQVA--- 56
Reticulitermes flavipes (ACV86032) KYLEELDRFYSQVA--- 53
Drosophila virilis (XP_002054350) KYLQDLDVYYGDRA--- 58

Euphausia crystallorophias NPF1 ----RPRFGKRSEY-AMAPRDALME--AS----------EKLMETFEHQR-- 90
Euphausia crystallorophias NPF1-L NVRARPRFGKRSEY-AMAPRDALME--AS----------EKLME-------- 128
Litopenaeus vannamei (AEC12204) ----RPRFGKRSDY-AMPSGDALME--AS----------ERLLETLARRR-- 90
Litopenaeus vannamei (AEC12205) SLRARPRFGKRSDY-AMPSGDALME--AS----------ERLLETLARRR-- 127
Melicertus marginatus (AEC12206) ----RPRFGKRSDY-PMPSGDALME--AS----------ERLLETLARRR-- 90
Melicertus marginatus (AEC12207) SLRARPRFGKRSDY-PMPSGDALME--AS----------ERLLETLARRR-- 127
Schistocerca gregaria (AFD50506) ----RPRFGKRSELRPDVVDDVIPEETSA----------DKFWRRFARRR-- 98
Schistocerca gregaria (AFS50166) TPRNRPRFGKRSELRPDVVDDVIPEETSA----------DKFWRRFARRR-- 148
Daphnia pulex (EFX80468) ----RPR--- 77
Locusta migratoria (P86442) ----RPRFGKRAELRPDVVDDVIPEEMSA----------DKFWRRFARRR-- 92
Reticulitermes flavipes (ACV86032) ----RPRFGKRAELRPVTEQEAAPDD-SS----------DSLWRQFASRR-- 88
Drosophila virilis (XP_002054350) ----RVRFGKRGSVMQAIRNRVLENGINSYNNENINDNGGELIHVAGEDEMF 106

Figure S2

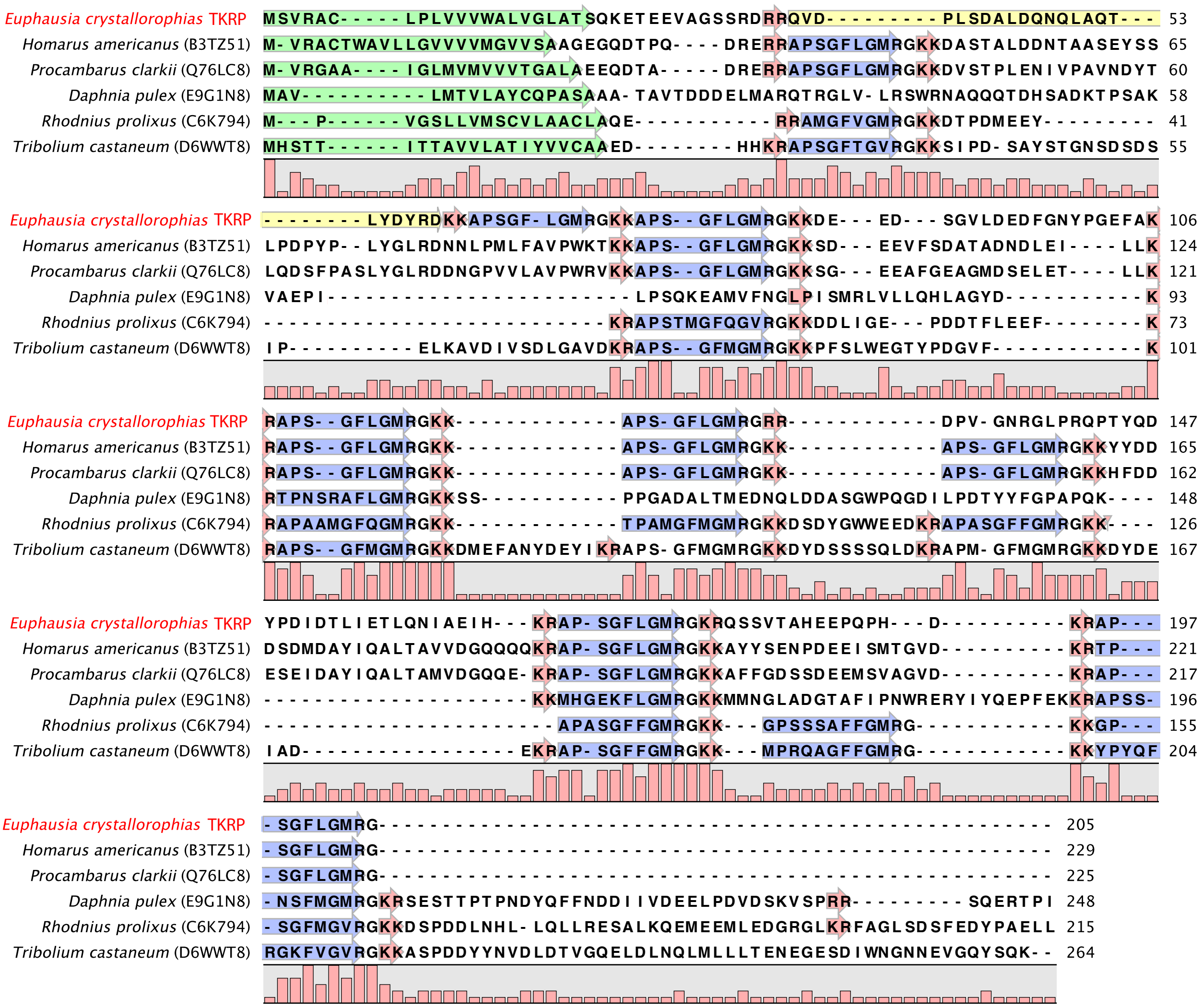

Euphausia crystallorophias TKRP MSVRAC-----LPLVVVWALVGLATSQKETEEVAGSSRDRRQVD---------PLSDALDQNQLAQT--- 53
Homarus americanus (B3TZ51) M-VRACTWAVLLGVVVVMGVVSAAGEGQDTPQ----DRERRAPSGFLGMRGKKDASTALDDNTAASEYSS 65
Procambarus clarkii (Q76LC8) M-VRGAA----IGLMVMVVVTGALAEEQDTA-----DRERRAPSGFLGMRGKKDVSTPLENIVPAVNDYT 60
Daphnia pulex (E9G1N8) MAV----------LMTVLAYCQPASAAA-TAVTDDDELMARQTRGLV-LRSWRNAQQQTDHSADKTPSAK 58
Rhodnius prolixus (C6K794) M---P------VGSLLVMSCVLAACLAQE-----------RRAMGFVGMRGKKDTPDMEEY--------- 41
Tribolium castaneum (D6WWT8) MHSTT------ITTAVVLATIYVVCAAED--------HHKRAPSGFTGVRGKKSIPD-SAYSTGNSDSDS 55
Euphausia crystallorophias TKRP --------LYDYRDKKAPSGF-LGMRGKKAPS--GFLGMRGKKDE---ED---SGVLDEDFGNYPGEFAK 106
Homarus americanus (B3TZ51) LPDPYP--LYGLRDNNLPMLFAVPWKTKKAPS--GFLGMRGKKSD---EEVFSDATADNDLEI----LLK 124
Procambarus clarkii (Q76LC8) LQDSFPASLYGLRDDNGPVVLAVPWRVKKAPS--GFLGMRGKKSG---EEAFGEAGMDSELET----LLK 121
Daphnia pulex (E9G1N8) VAEPI-----------------------LPSQKEAMVFNGLPISMRLVLLQHLAGYD-----------K 93
Rhodnius prolixus (C6K794) --------------------------KRAPSTMGFQGVRGKKDDLIGE---PDDTFLEEF--------K 73
Tribolium castaneum (D6WWT8) IP----------ELKAVDIVSDLGAVDKRAPS--GFMGMRGKKPFSLWEGTYPDGVF------------K 101
Euphausia crystallorophias TKRP RAPS--GFLGMRGKK-------------APS-GFLGMRGRR------------DPV-GNRGLPRQPTYQD 147
Homarus americanus (B3TZ51) RAPS--GFLGMRGKK-------------APS-GFLGMRGKK------------APS-GFLGMRGKKYYDD 165
Procambarus clarkii (Q76LC8) RAPS--GFLGMRGKK-------------APS-GFLGMRGKK------------APS-GFLGMRGKKHFDD 162
Daphnia pulex (E9G1N8) RTPNSRAFLGMRGKKSS-----------PPGADALTMEDNQLDDASGWPQGDILPDTYYFGPAPQK---- 148
Rhodnius prolixus (C6K794) RAPAAMGFQGMRGKK-------------TPAMGFMGMRGKKDSDYGWWEEDKRAPASGFFGMRGKK---- 126
Tribolium castaneum (D6WWT8) RAPS--GFMGMRGKKDMEFANYDEYIKRAPS-GFMGMRGKKDYDSSSSQLDKRAPM-GFMGMRGKKDYDE 167
Euphausia crystallorophias TKRP YPDIDTLIETLQNIAEIH---KRAP-SGFLGMRGKRQSSVTAHEEPQPH---D----------KRAP--- 197
Homarus americanus (B3TZ51) DSDMDAYIQALTAVVDGQQQQKRAP-SGFLGMRGKKAYYSENPDEEISMTGVD----------KRTP--- 221
Procambarus clarkii (Q76LC8) ESEIDAYIQALTAMVDGQQE-KRAP-SGFLGMRGKKAFFGDSSDEEMSVAGVD----------KRAP--- 217
Daphnia pulex (E9G1N8) ---------------------KKMHGEKFLGMRGKKMMNGLADGTAFIPNWRERYIYQEPFEKKRAPSS- 196
Rhodnius prolixus (C6K794) -----------------------APASGFFGMRGKK---GPSSSAFFGMRG-----------KKGP--- 155
Tribolium castaneum (D6WWT8) IAD-----------------EKRAP-SGFFGMRGKK---MPRQAGFFGMRG------------KKYPYQF 204
Euphausia crystallorophias TKRP -SGFLGMRG--- 205
Homarus americanus (B3TZ51) -SGFLGMRG--- 229
Procambarus clarkii (Q76LC8) -SGFLGMRG--- 225
Daphnia pulex (E9G1N8) -NSFMGMRGKRSESTTPTPNDYQFFNDDIIVDEELPDVDSKVSPRR---------SQERTPI 248
Rhodnius prolixus (C6K794) -SGFMGVRGKKDSPDDLNHL-LQLLRESALKQEMEEMLEDGRGLKRFAGLSDSFEDYPAELL 215
Tribolium castaneum (D6WWT8) RGKFVGVRGKKASPDDYYNVDLDTVGQELDLNQLMLLLTENEGESDIWNGNNEVGQYSQK-- 264

Figure S3a

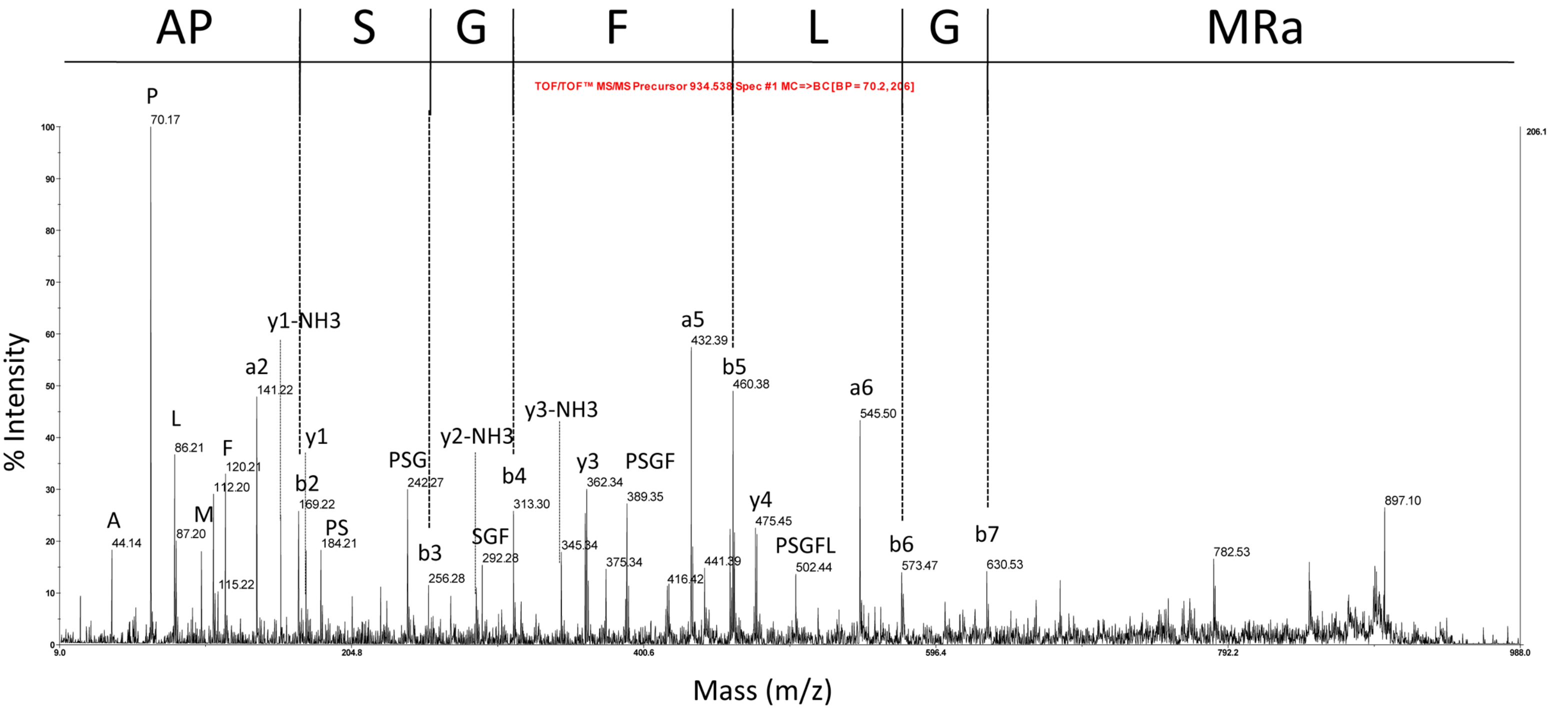

APSGFLGMRa

Figure S3b

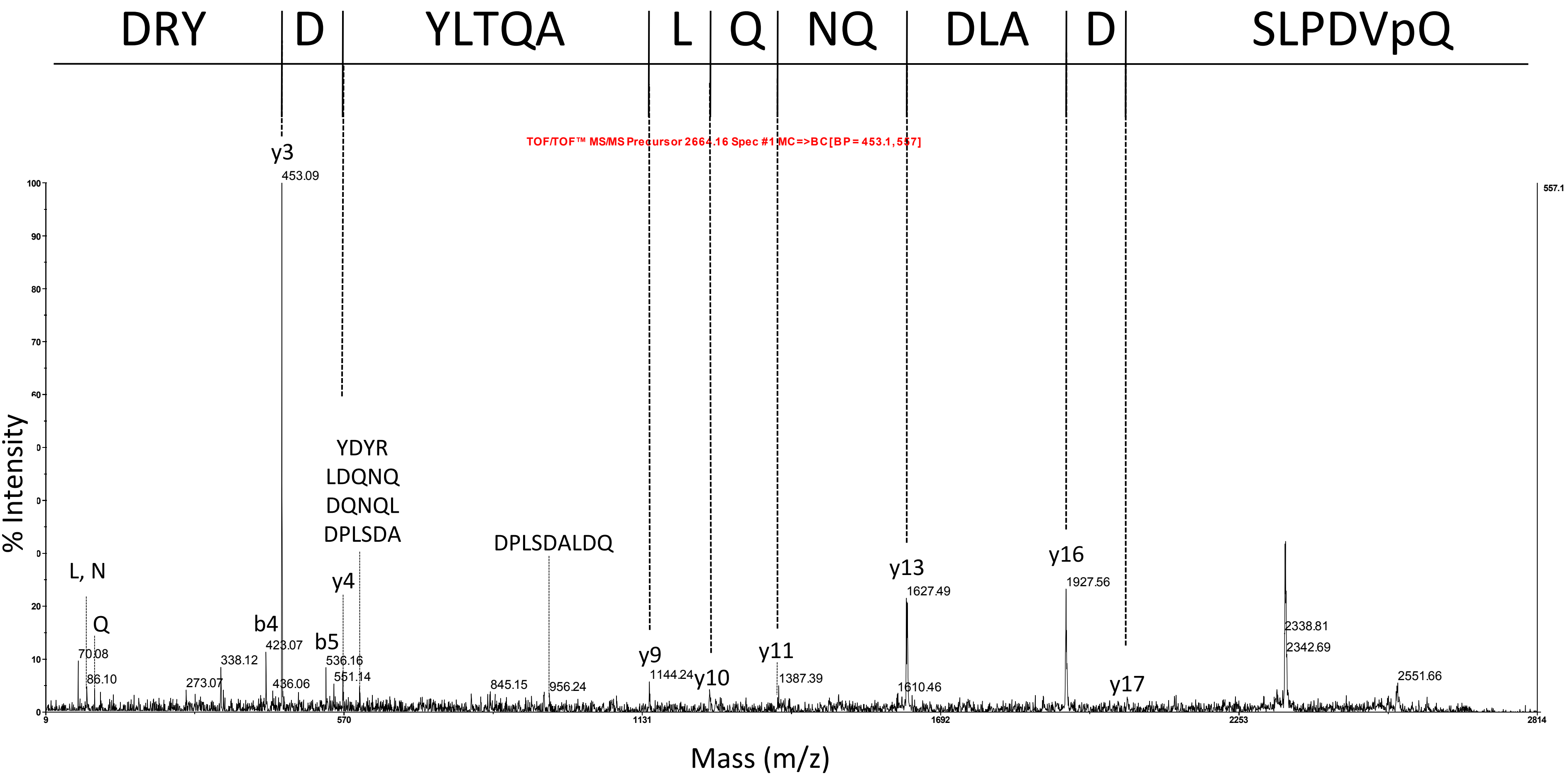

Figure S3c

pQVDPLSDALDQNQLAQTLYDYRD

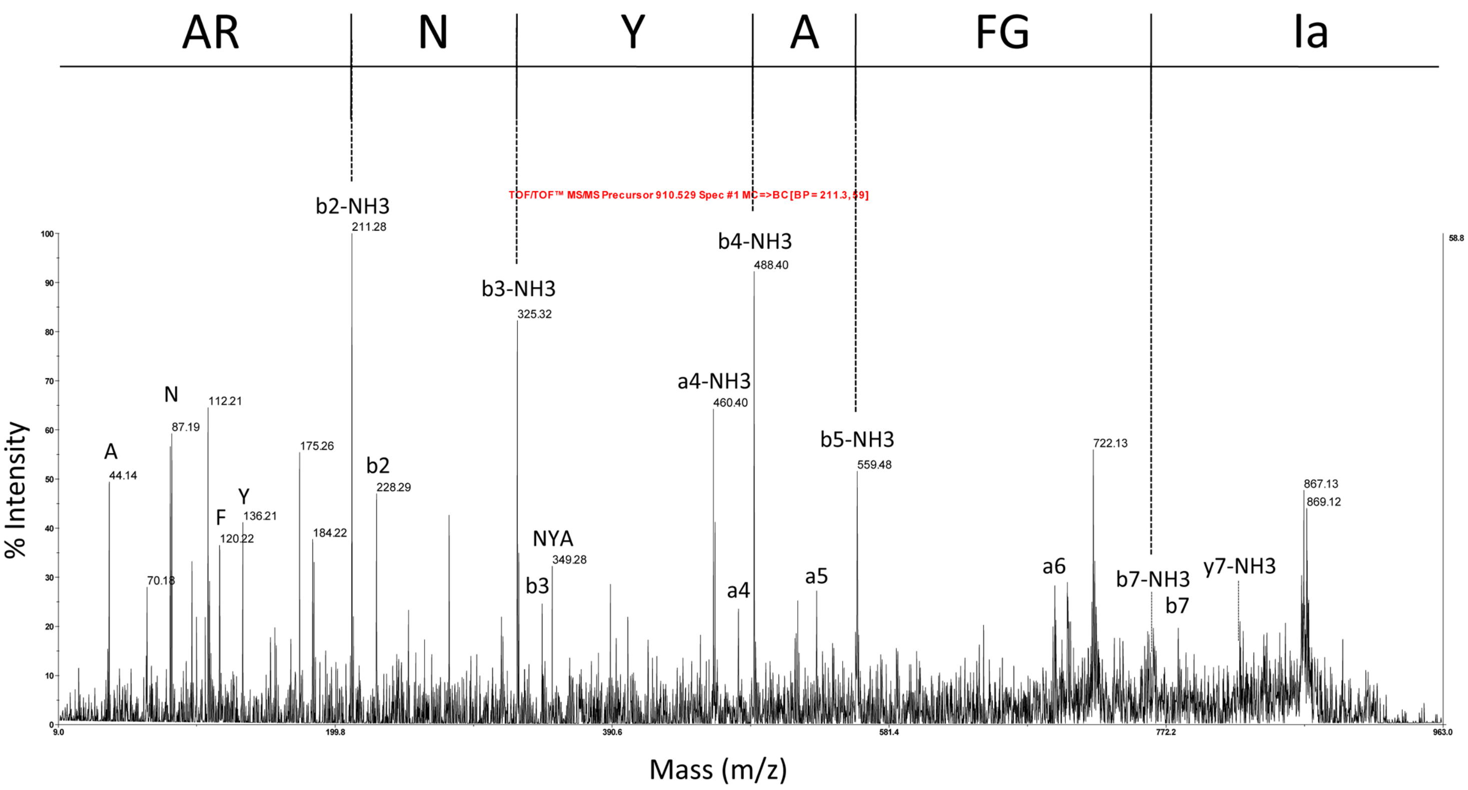

Figure S4

ARNYAFGIa

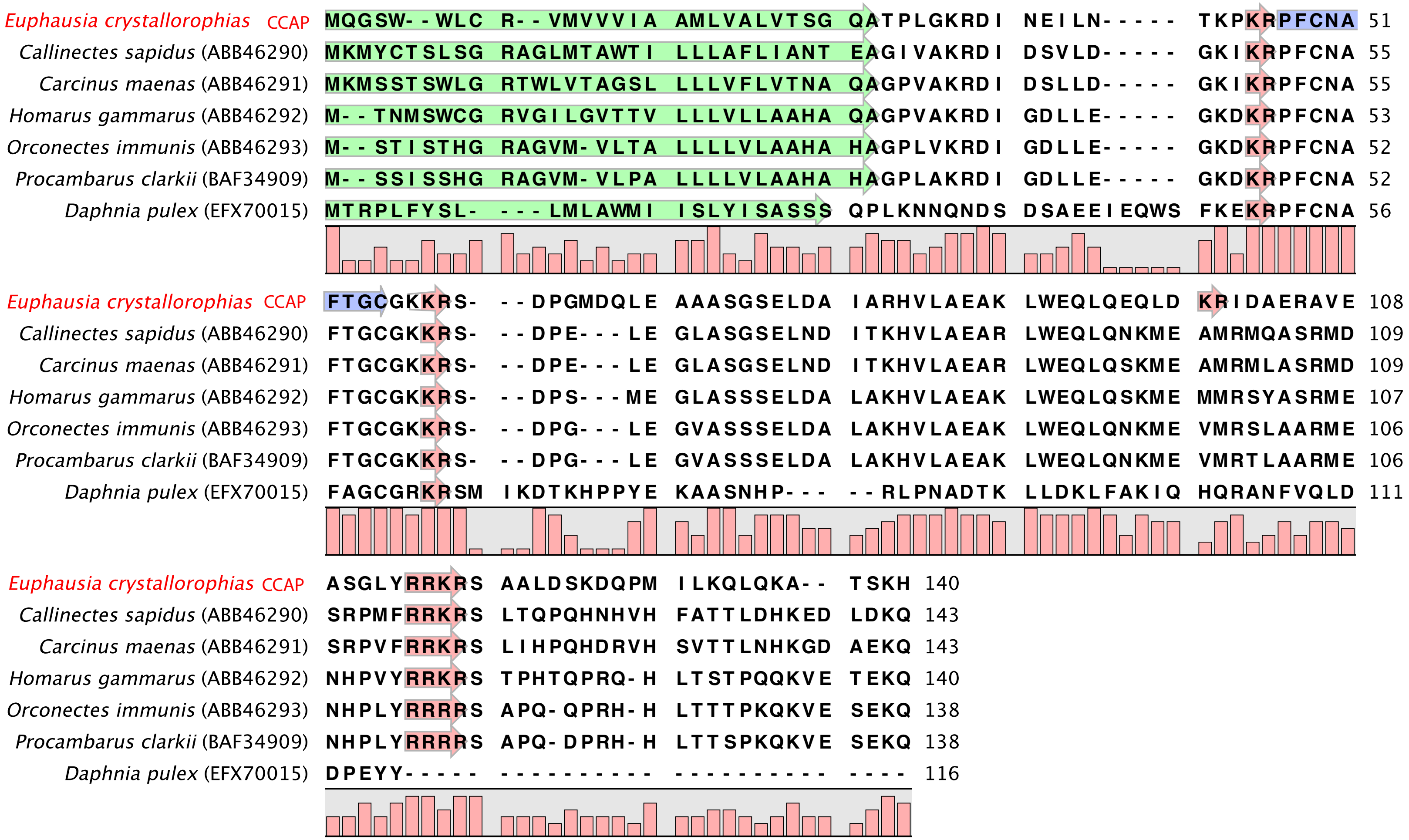
Euphausia crystallorophias CCAP MQGSW--WLC R--VMVVVIA AMLVALVTSG QATPLGKRDI NEILN----- TKPKRPFCNA 51
Callinectes sapidus (ABB46290) MKMYCTSLSG RAGLMTAWTI LLLAFLIANT EAGIVAKRDI DSVLD----- GKIKRPFCNA 55
Carcinus maenas (ABB46291) MKMSSTSWLG RTWLVTAGSL LLLVFLVTNA QAGPVAKRDI DSLLD----- GKIKRPFCNA 55
Homarus gammarus (ABB46292) M--TNMSWCG RVGILGVTTV LLLVLLAAHA QAGPVAKRDI GDLLE----- GKDKRPFCNA 53
Orconectes immunis (ABB46293) M--STISTHG RAGVM-VLTA LLLLVLAAHA HAGPLVKRDI GDLLE----- GKDKRPFCNA 52
Procambarus clarkii (BAF34909) M--SSISSHG RAGVM-VLPA LLLLVLAAHA HAGPLAKRDI GDLLE----- GKDKRPFCNA 52
Daphnia pulex (EFX70015) MTRPLFYSL- ---LMLAWMI ISLYISASSS QPLKNNQNDS DSAEEIEQWS FKEKRPFCNA 56
Euphausia crystallorophias CCAP FTGCGKKRS- --DPGMDQLE AAASGSELDA IARHVLAEAK LWEQLQEQLD KRIDAERAVE 108
Callinectes sapidus (ABB46290) FTGCGKKRS- --DPE---LE GLASGSELND ITKHVLAEAR LWEQLQNKME AMRMQASRMD 109
Carcinus maenas (ABB46291) FTGCGKKRS- --DPE---LE GLASGSELND ITKHVLAEAR LWEQLQSKME AMRMLASRMD 109
Homarus gammarus (ABB46292) FTGCGKKRS- --DPS---ME GLASSSELDA LAKHVLAEAK LWEQLQSKME MMRSYASRME 107
Orconectes immunis (ABB46293) FTGCGKKRS- --DPG---LE GVASSSELDA LAKHVLAEAK LWEQLQNKME VMRSLAARME 106
Procambarus clarkii (BAF34909) FTGCGKKRS- --DPG---LE GVASSSELDA LAKHVLAEAK LWEQLQNKME VMRTLAARME 106
Daphnia pulex (EFX70015) FAGCGRKRSM IKDTKHPPYE KAASNHP--- --RLPNADTK LLDKLFAKIQ HQRANFVQLD 111
Euphausia crystallorophias CCAP ASGLYRRKRS AALDSKDQPM ILKQLQKA-- TSKH 140
Callinectes sapidus (ABB46290) SRPMFRRKRS LTQPQHNHVH FATTLDHKED LDKQ 143
Carcinus maenas (ABB46291) SRPVFRRKRS LIHPQHDRVH SVTTLNHKGD AEKQ 143
Homarus gammarus (ABB46292) NHPVYRRKRS TPHTQPRQ-H LTSTPQQKVE TEKQ 140
Orconectes immunis (ABB46293) NHPLYRRRRS APQ-QPRH-H LTTTPKQKVE SEKQ 138
Procambarus clarkii (BAF34909) NHPLYRRRRS APQ-DPRH-H LTTSPKQKVE SEKQ 138
Daphnia pulex (EFX70015) DPEYY----- ---------- ---------- ---- 116

Figure S5

Euphausia crystallorophias CHH	SIFDPSCKGF	YNKEVFKKLN	HICDDCYNLY	RDASVAVKCK	ENCFGNPVFE	QCIYELLIDD	QVDELSKIVR	TL	72
Penaeus monodon (AAQ24525)	SLSFRSCTGA	YDRELLVRLD	RVCEDCYNVY	RDVGVAAECR	SNCFHNEVFL	YCVDYMFRPR	QRNQYRAALQ	RL	72
Marsupenaeus japonicus (Q94676)	SLFDPACTGI	YDRQLLRKLG	RLCDDCYNVF	REPKVATGCR	SNCYHNLIFL	DCLEYLIPSH	LQEEHMAAMQ	TV	72
Penaeus monodon (O97383)	SLFDPSCTGV	FDRQLLRRLS	RVCDDCFNVF	REPNVATECR	SNCYNNEVFR	QCMEYLLPAH	LHEEHRLAVQ	MV	72
Litopenaeus vannamei (Q26181)	SLFDPSCTGV	FDRQLLRRLR	RVCDDCFNVF	REPNVSTECR	SNCYNNEVFR	QCMEYLLPPH	LHEEHRLAVQ	MV	72
Penaeus monodon (O97384)	ANFDPSCAGV	YNRELLGRLS	RLCDDCYNVF	REPKVATECR	SNCFYNPVFV	QCLEYLIPAD	LHEEYQAHVQ	TV	72
Litopenaeus schmitti (P59685)	ANFDPSCTGV	YDRELLGRLS	RLCDDCYNVF	REPKVATECR	SNCFYNPVFV	QCLEYLIPAD	LHEEYQAHVQ	TV	72
Marsupenaeus japonicus (O15981)	LVFDPSCAGV	YDRVLLGKLN	RLCDDCYNVF	REPNVATECR	SNCFYNLAFV	QCLEYLMPPS	LHEEYQANVQ	MV	72
Metapenaeus ensis (AAD11813)	SLFDPSCSGV	FDRELLGRLN	RVCDDCYNVF	RDPKVAMECK	SNCFLNPAFI	QCLEYLLPED	LHEEYQSHVQ	VV	72
Armadillidium vulgare (P30814)	RIFDTSCKGF	YDRGLFAQLD	RVCEDCYNLY	RKPHVAAECR	RDCYTTEVFE	SCLKDLMMHD	FINEYKEMAL	MV	72
Pagurus bernhardus (ABE02191)	QAFDRSCKGV	YDRGLFKKLE	RVCDDCYNLY	RKPYVEVGCK	ANCYANSIFR	QCIGDLLLED	VVEEYAQAIQ	MV	72
Cherax destructor (P83485)	QVFDQACKGV	YDRAIFKKLD	RVCDDCYNLY	RKPYVAVSCR	GNCYNNLVFR	QCLEELFLGN	GFNEYISGVQ	TV	72
Cherax destructor (P83486)	QVFDQACKGV	YDRAIFKKLD	RVCDDCYNLY	RKPYVATSCR	QNCYSNLVFR	QCLDDLLLVD	VVDEYVSGVQ	IV	72
Astacus astacus (P83800)	QVFDQACKGI	YDRAIFKKLD	RVCDDCYNLY	RKPYVATTCR	QNCYANSVFR	QCLDDLLLID	VVDEYISGVQ	IV	72
Procambarus bouvieri (P55845)	QVFDQACKGI	YDRAIFKKLD	RVCEDCYNLY	RKPYVATTCR	QNCYANSVFR	QCLDDLLLID	VVDEYISGVQ	TV	72
Procambarus clarkii (Q25683)	QVFDQACKGI	YDRAIFKKLD	RVCEDCYNLY	RKPYVATTCR	QNCYANSVFR	QCLDDLLLID	VVDEYISGVQ	TV	72
Orconectes limosus (Q25589)	QVFDQACKGI	YDRAIFKKLD	RVCEDCYNLY	RKPYVATTCR	QNCYANSVFR	QCLDDLLLID	VLDEYISGVQ	TV	72
Nephrops norvegicus (AAQ22391)	QVFDQACKGV	YDRNLFKKLD	RVCEDCYNLY	RKPFVATTCR	ENCYSNRVFR	QCLDDLLLID	VIDEYVSSVQ	MV	72
Homarus americanus (P19806)	QVFDQACKGV	YDRNLFKKLD	RVCEDCYNLY	RKPFVATTCR	ENCYSNWVFR	QCLDDLLLSD	VIDEYVSNVQ	MV	72
Homarus gammarus (ABA42179)	QVFDQACKGV	YDRNLFKKLD	RVCEDCYNLY	RKPFVATTCR	ENCYSNWVFR	QCLDDLLLSD	VVDEYVSNVQ	MV	72
Macrobrachium rosenbergii (AAL40915)	AILDQSCKGI	FDRELFKKLD	RVCDDCYNLY	RKPYVAIDCR	EGCYQNLVFR	QCIQDLQLMD	QLDEYANAVQ	IV	72
Callinectes sapidus (AAS45136)	QIYDSSCKGV	YDRAIFNELE	HVCDDCYNLY	RNSRVASGCR	SNCYSNMVIR	QCMEDLLIMD	NFEEYARKIQ	VV	72
Scylla olivacea (AAQ75760)	QIFDSSCKGV	YDRAIFNELE	HVCNDCYNLY	RTSHVASGCR	SNCYSNVVIR	QCMEDLLLMD	NFEEIARKIQ	MV	72
Libinia emarginata (P56688)	QIFDPSCKGL	YDRGLFSDLE	HVCKDCYNLY	RNPQVTSACR	VNCYSNRVFR	QCMEDLLLME	DFDKYARAIQ	TV	72
Bythograea thermydron (AAK28329)	QIYDRSCKGL	YDRRLFSDLD	HVCDDCYNLY	RNSRVANACR	ENCYSNLVFR	QCMEDLLLMD	QFDKYARAVQ	TV	72
Cancer pagurus (P81032)	QIYDTSCKGV	YDRGLFSDLE	HVCDDCYNLY	RNSYVASACR	SNCYSNVVFR	QCMEELLLMD	EFDKYARAVQ	IV	72
Cancer productus (ABQ41269)	QIYDSSCKGV	YDRGLFSDLE	HVCDDCYNLY	RNSYVASACR	SNCYSNVVFR	QCMEELLLME	EFDKYARAVQ	IV	72
Carcinus maenas (P14944)	QIYDTSCKGV	YDRALFNDLE	HVCDDCYNLY	RTSYVASACR	SNCYSNLVFR	QCMDDLLMMD	EFDQYARKVQ	MV	72
Gecarcoidea natalis (ABL09570)	QIYDRSCKGV	YDRSLFNKLE	HVCDDCYNLY	RTSFVYSSCR	ENCYSNLVFR	QCMEDLLLMD	VFDEYAKAVQ	VV	72
Gecarcinus lateralis (ABF48652)	QIYDRSCKGV	YDRSLFNKLE	HVCDDCYNLY	RTSFVYSSCR	ENCYSNLVFR	QCMEDLLLMD	VFDEYAKAVQ	VV	72
Potamon ibericum (ABA70560)	QIYDRSCKGV	YDRSLFSKLE	HVCDDCYNLY	RTSYVASACR	ENCYSNLVFR	QCMDDLLLMD	VFDEYAKAVQ	MV	72
Pachygrapsus marmoratus (AAO27804)	QIYDRSCKGV	YDRSLFGKLE	HVCDDCCNLY	RTHHVASACR	ENCYSNLVFR	QCMDDLLLMD	VFDEYAKAVQ	MV	72

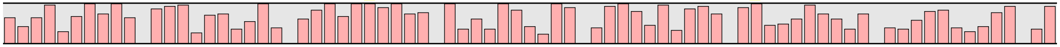

Figure S6a

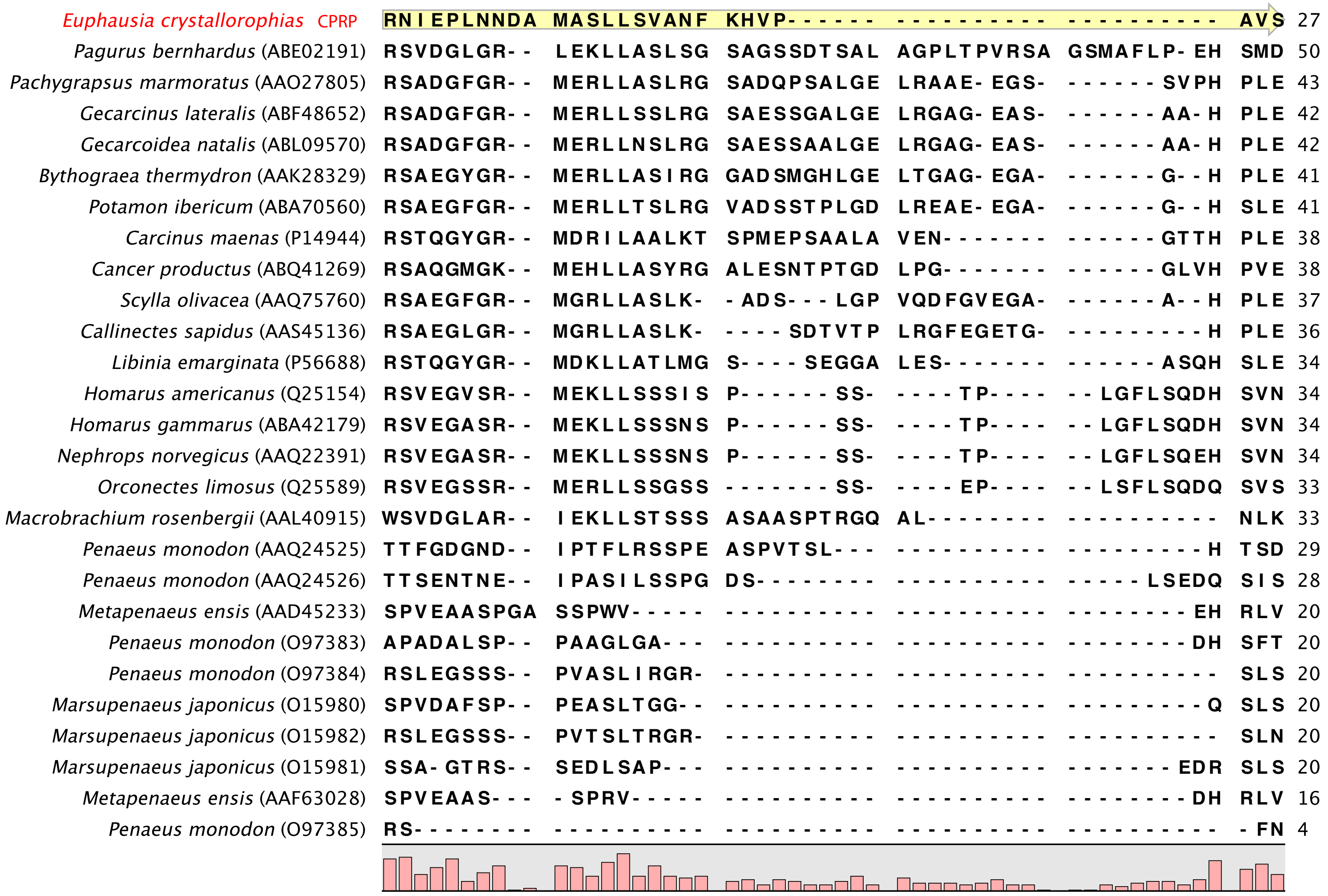
Euphausia crystallorophias CPRP RNIEPLNNDA MASLLSVANF KHVP------ ---------- ---------- AVS 27
Pagurus bernhardus (ABE02191) RSVDGLGR-- LEKLLASLSG SAGSSDTSAL AGPLTPVRSA GSMAFLP-EH SMD 50
Pachygrapsus marmoratus (AAO27805) RSADGFGR-- MERLLASLRG SADQPSALGE LRAAE-EGS- ------SVPH PLE 43
Gecarcinus lateralis (ABF48652) RSADGFGR-- MERLLSSLRG SAESSGALGE LRGAG-EAS- ------AA-H PLE 42
Gecarcoidea natalis (ABL09570) RSADGFGR-- MERLLNSLRG SAESSAALGE LRGAG-EAS- ------AA-H PLE 42
Bythograea thermydron (AAK28329) RSAEGYGR-- MERLLASIRG GADSMGHLGE LTGAG-EGA- ------G--H PLE 41
Potamon ibericum (ABA70560) RSAEGFGR-- MERLLTSLRG VADSSTPLGD LREAE-EGA- ------G--H SLE 41
Carcinus maenas (P14944) RSTQGYGR-- MDRILAALKT SPMEPSAALA VEN------- ------GTTH PLE 38
Cancer productus (ABQ41269) RSAQGMGK-- MEHLLASYRG ALESNTPTGD LPG------- ------GLVH PVE 38
Scylla olivacea (AAQ75760) RSAEGFGR-- MGRLLASLK- -ADS---LGP VQDFGVEGA- ------A--H PLE 37
Callinectes sapidus (AAS45136) RSAEGLGR-- MGRLLASLK- ----SDTVTP LRGFEGETG- ---------H PLE 36
Libinia emarginata (P56688) RSTQGYGR-- MDKLLATLMG S----SEGGA LES------- ------ASQH SLE 34
Homarus americanus (Q25154) RSVEGVSR-- MEKLLSSSIS P------SS- ----TP---- --LGFLSQDH SVN 34
Homarus gammarus (ABA42179) RSVEGASR-- MEKLLSSSNS P------SS- ----TP---- --LGFLSQDH SVN 34
Nephrops norvegicus (AAQ22391) RSVEGASR-- MEKLLSSSNS P------SS- ----TP---- --LGFLSQEH SVN 34
Orconectes limosus (Q25589) RSVEGSSR-- MERLLSSGSS -------SS- ----EP---- --LSFLSQDQ SVS 33
Macrobrachium rosenbergii (AAL40915) WSVDGLAR-- IEKLLSTSSS ASAASPTRGQ AL-------- ---------- NLK 33
Penaeus monodon (AAQ24525) TTFGDGND-- IPTFLRSSPE ASPVTSL--- ---------- ---------H TSD 29
Penaeus monodon (AAQ24526) TTSENTNE-- IPASILSSPG DS-------- ---------- -----LSEDQ SIS 28
Metapenaeus ensis (AAD45233) SPVEAASPGA SSPWV----- ---------- ---------- --------EH RLV 20
Penaeus monodon (O97383) APADALSP-- PAAGLGA--- ---------- ---------- --------DH SFT 20
Penaeus monodon (O97384) RSLEGSSS-- PVASLIRGR- ---------- ---------- ---------- SLS 20
Marsupenaeus japonicus (O15980) SPVDAFSP-- PEASLTGG-- ---------- ---------- ---------Q SLS 20
Marsupenaeus japonicus (O15982) RSLEGSSS-- PVTSLTRGR- ---------- ---------- ---------- SLN 20
Marsupenaeus japonicus (O15981) SSA-GTRS-- SEDLSAP--- ---------- ---------- -------EDR SLS 20
Metapenaeus ensis (AAF63028) SPVEAAS--- -SPRV----- ---------- ---------- --------DH RLV 16
Penaeus monodon (O97385) RS-------- ---------- ---------- ---------- ---------- -FN 4

Figure S6b

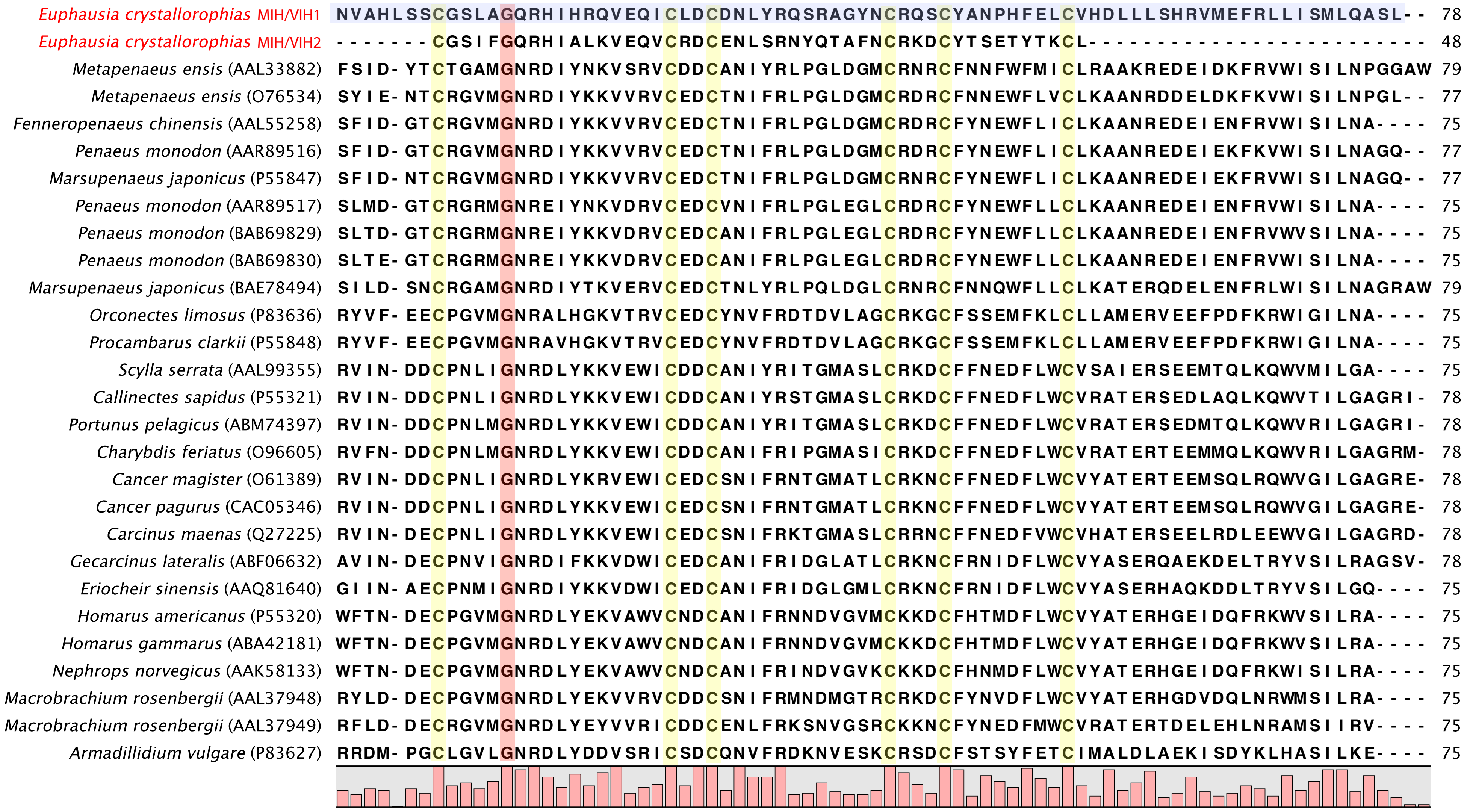
Euphausia crystallorophias MIH/VIH1 78
Euphausia crystallorophias MIH/VIH2 48
Metapenaeus ensis (AAL33882) 79
Metapenaeus ensis (O76534) 77
Fenneropenaeus chinensis (AAL55258) 75
Penaeus monodon (AAR89516) 77
Marsupenaeus japonicus (P55847) 77
Penaeus monodon (AAR89517) 75
Penaeus monodon (BAB69829) 75
Penaeus monodon (BAB69830) 75
Marsupenaeus japonicus (BAE78494) 79
Orconectes limosus (P83636) 75
Procambarus clarkii (P55848) 75
Scylla serrata (AAL99355) 75
Callinectes sapidus (P55321) 78
Portunus pelagicus (ABM74397) 78
Charybdis feriatus (O96605) 78
Cancer magister (O61389) 78
Cancer pagurus (CAC05346) 78
Carcinus maenas (Q27225) 78
Gecarcinus lateralis (ABF06632) 78
Eriocheir sinensis (AAQ81640) 75
Homarus americanus (P55320) 75
Homarus gammarus (ABA42181) 75
Nephrops norvegicus (AAK58133) 75
Macrobrachium rosenbergii (AAL37948) 75
Macrobrachium rosenbergii (AAL37949) 75
Armadillidium vulgare (P83627) 75

Figure S7

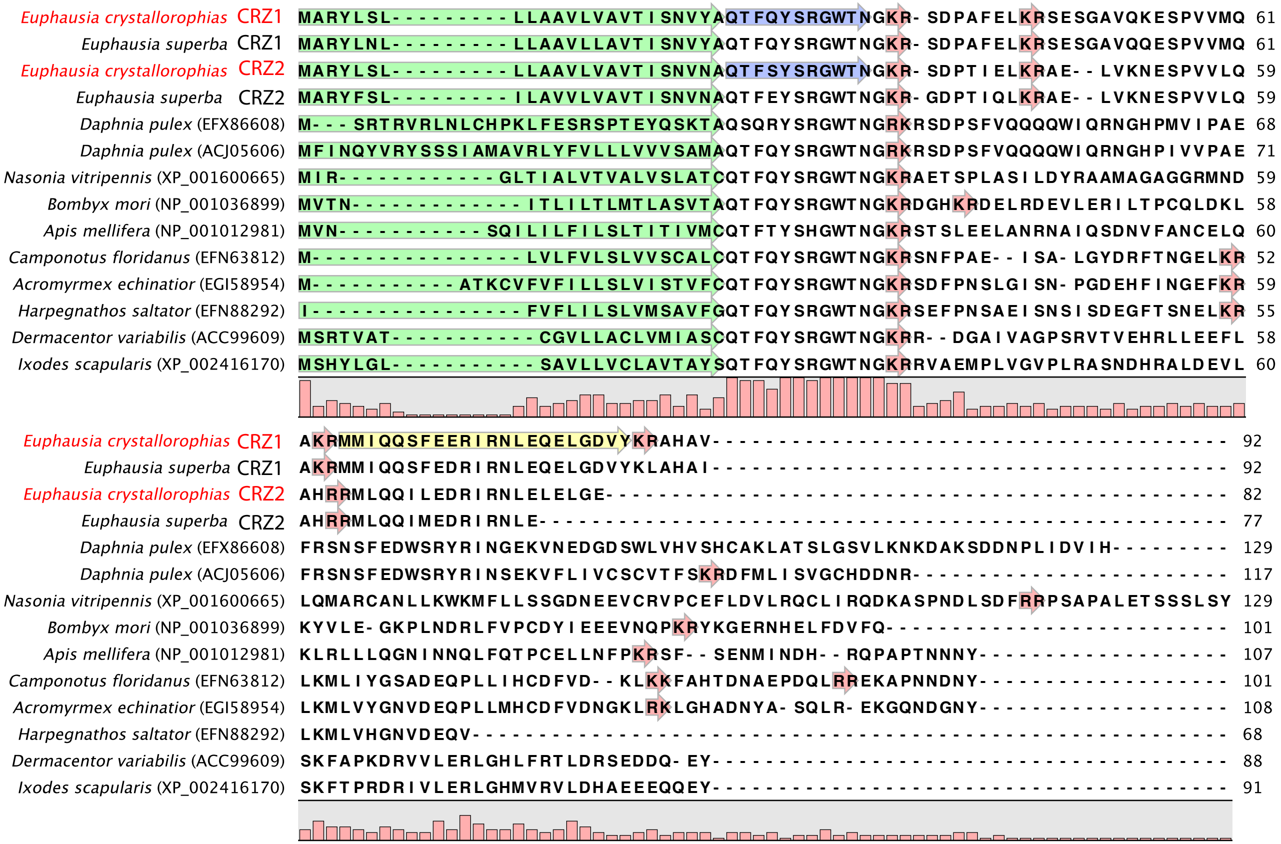
Euphausia crystallorophias CRZ1 MARYLSL---------LLAAVLVAVTISNVYAQTFQYSRGWTNGKR-SDPAFELKRSESGAVQKESPVVMQ 61
Euphausia superba CRZ1 MARYLNL---------LLAAVLLAVTISNVYAQTFQYSRGWTNGKR-SDPAFELKRSESGAVQQESPVVMQ 61
Euphausia crystallorophias CRZ2 MARYLSL---------LLAAVLVAVTISNVNAQTFSYSRGWTNGKR-SDPTIELKRAE--LVKNESPVVLQ 59
Euphausia superba CRZ2 MARYFSL---------ILAVVLVAVTISNVNAQTFEYSRGWTNGKR-GDPTIQLKRAE--LVKNESPVVLQ 59
Daphnia pulex (EFX86608) M---SRTRVRLNLCHPKLFESRSPTEYQSKTAQSQRYSRGWTNGRKRSDPSFVQQQQWIQRNGHPMVIPAE 68
Daphnia pulex (ACJ05606) MFINQYVRYSSSIAMAVRLYFVLLLVVVSAMAQTFQYSRGWTNGRKRSDPSFVQQQQWIQRNGHPIVVPAE 71
Nasonia vitripennis (XP_001600665) MIR-----------GLTIALVTVALVSLATCQTFQYSRGWTNGKRAETSPLASILDYRAAMAGAGGRMND 59
Bombyx mori (NP_001036899) MVTN------------ITLILTLMTLASVTAQTFQYSRGWTNGKRDGHKRDELRDEVLERILTPCQLDKL 58
Apis mellifera (NP_001012981) MVN-----------SQILILFILSLTITIVMCQTFTYSHGWTNGKRSTSLEELANRNAIQSDNVFANCELQ 60
Camponotus floridanus (EFN63812) M---------------LVLFVLSLVVSCALCQTFQYSRGWTNGKRSNFPAE--ISA-LGYDRFTNGELKR 52
Acromyrmex echinatior (EGI58954) M-----------ATKCVFVFILLSLVISTVFCQTFQYSRGWTNGKRSDFPNSLGISN-PGDEHFINGEFKR 59
Harpegnathos saltator (EFN88292) I---------------FVFLILSLVMSAVFGQTFQYSRGWTNGKRSEFPNSAEISNSISDEGFTSNELKR 55
Dermacentor variabilis (ACC99609) MSRTVAT-----------CGVLLACLVMIASCQTFQYSRGWTNGKRR--DGAIVAGPSRVTVEHRLLEEFL 58
Ixodes scapularis (XP_002416170) MSHYLGL-----------SAVLLVCLAVTAYSQTFQYSRGWTNGKRRVAEMPLVGVPLRASNDHRALDEVL 60
Euphausia crystallorophias CRZ1 AKRMMIQQSFEERIRNLEQELGDVYKRAHAV------------------------------------- 92
Euphausia superba CRZ1 AKRMMIQQSFEDRIRNLEQELGDVYKLAHAI------------------------------------- 92
Euphausia crystallorophias CRZ2 AHRRMLQQILEDRIRNLELELGE--- 82
Euphausia superba CRZ2 AHRRMLQQIMEDRIRNLE-- 77
Daphnia pulex (EFX86608) FRSNSFEDWSRYRINGEKVNEDGDSWLVHVSHCAKLATSLGSVLKNKDAKSDDNPLIDVIH--------- 129
Daphnia pulex (ACJ05606) FRSNSFEDWSRYRINSEKVFLIVCSCVTFSKRDFMLISVGCHDDNR---------------------- 117
Nasonia vitripennis (XP_001600665) LQMARCANLLKWKMFLLSSGDNEEVCRVPCEFLDVLRQCLIRQDKASPNDLSDFRRPSAPALETSSSLSY 129
Bombyx mori (NP_001036899) KYVLE-GKPLNDRLFVPCDYIEEEVNQPKRYKGERNHELFDVFQ------------------------ 101
Apis mellifera (NP_001012981) KLRLLLQGNINNQLFQTPCELLNFPKRSF--SENMINDH--RQPAPTNNNY----------------- 107
Camponotus floridanus (EFN63812) LKMLIYGSADEQPLLIHCDFVD--KLKKFAHTDNAEPDQLRREKAPNNDNY----------------- 101
Acromyrmex echinatior (EGI58954) LKMLVYGNVDEQPLLMHCDFVDNGKLRKLGHADNYA-SQLR-EKGQNDGNY----------------- 108
Harpegnathos saltator (EFN88292) LKMLVHGNVDEQV--- 68
Dermacentor variabilis (ACC99609) SKFAPKDRVVLERLGHLFRTLDRSEDDQ-EY------------------------------------- 88
Ixodes scapularis (XP_002416170) SKFTPRDRIVLERLGHMVRVLDHAEEEQQEY------------------------------------- 91

Figure S8a

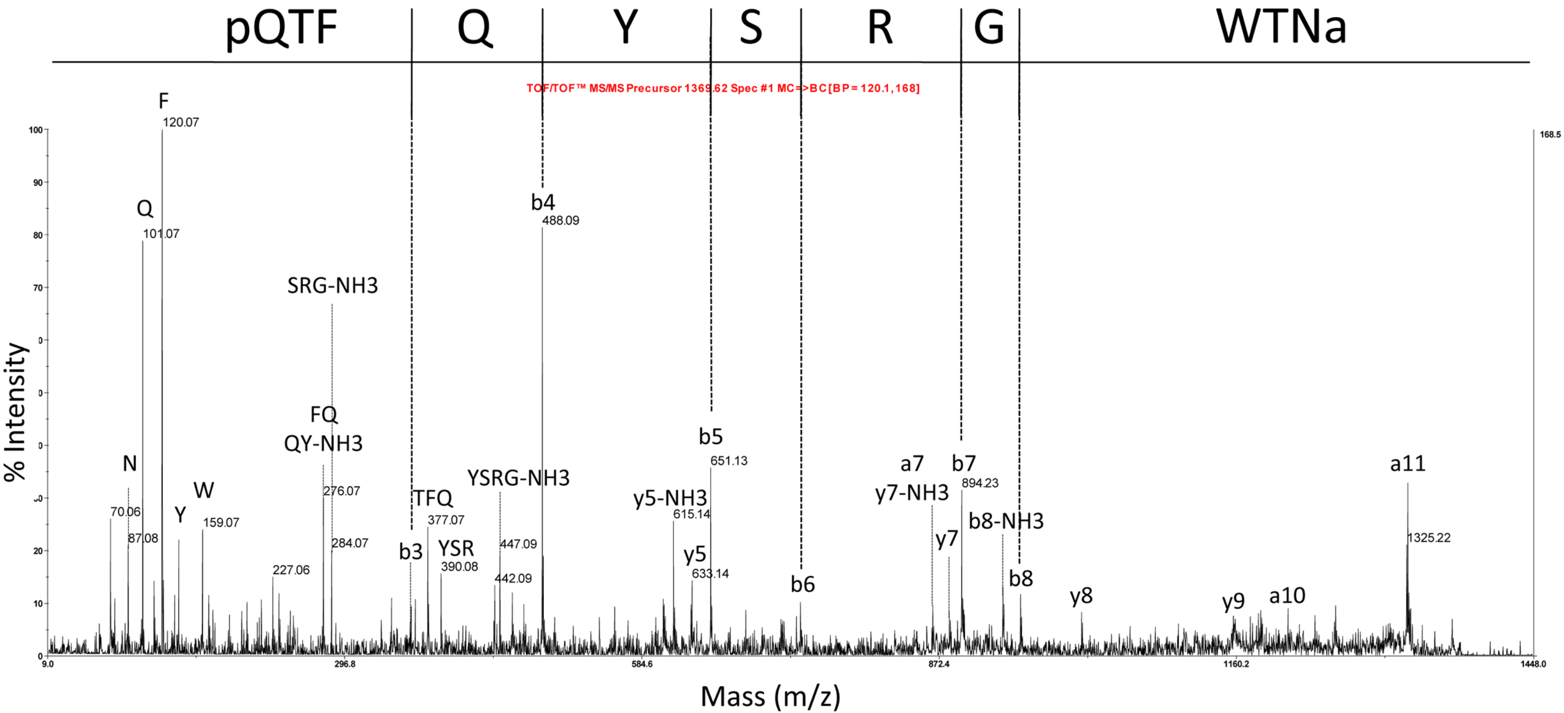

Figure S8b

pQTFQYSRGWTNa

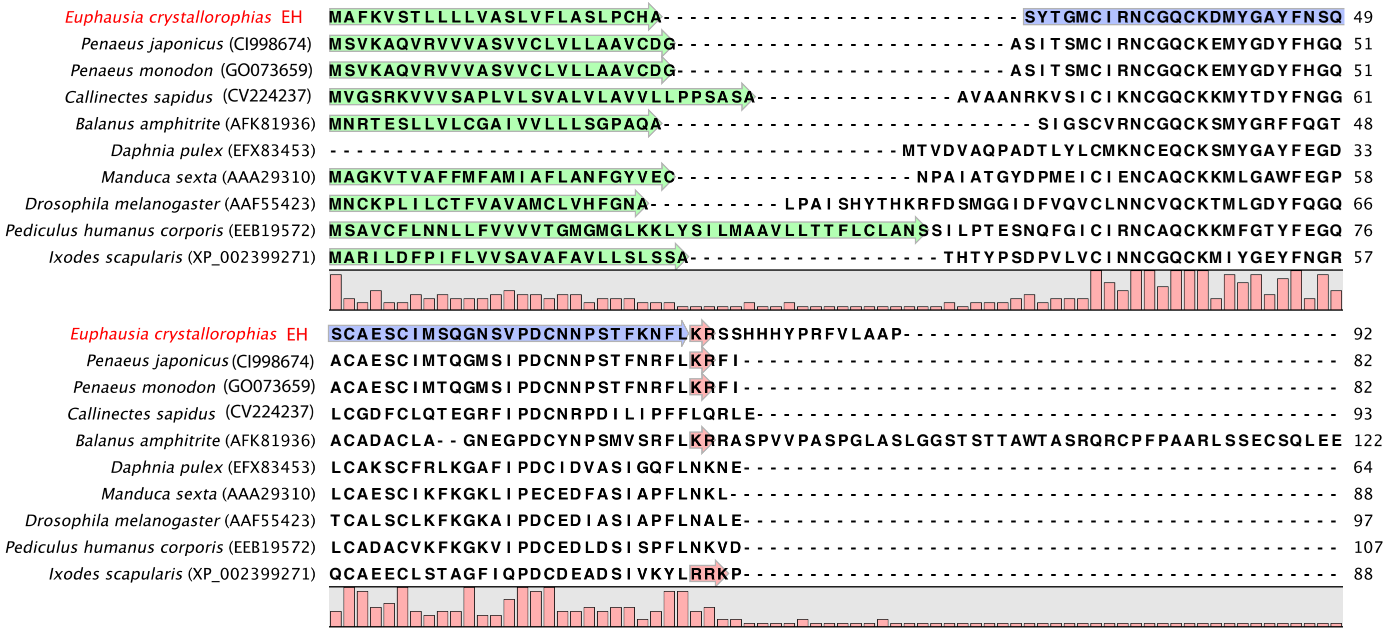

Euphausia crystallorophias EH MAFKVSTLLLLVASLVFLASLPCHA---------------------------- SYTGMCIRNCGQCKDMYGAYFNSQ 49
Penaeus japonicus (CI998674) MSVKAQVRVVVASVVCLVLLAAVCDG------------------------- ASITSMCIRNCGQCKEMYGDYFHGQ 51
Penaeus monodon (GO073659) MSVKAQVRVVVASVVCLVLLAAVCDG------------------------- ASITSMCIRNCGQCKEMYGDYFHGQ 51
Callinectes sapidus (CV224237) MVGSRKVVVSAPLVLSVALVLAVVLLPPSASA--------------- AVAANRKVSICIKNCGQCKKMYTDYFNGG 61
Balanus amphitrite (AFK81936) MNRTESLLVLCGAIVVLLLSGPAQA----------------------------- SIGSCVRNCGQCKSMYGRFFQGT 48
Daphnia pulex (EFX83453) -- MTVDVAQPADTLYLCMKNCEQCKSMYGAYFEGD 33
Manduca sexta (AAA29310) MAGKVTVAFFMFAMIAFLANFGYVEC------------------ NPAIATGYDPMEICIENCAQCKKMLGAWFEGP 58
Drosophila melanogaster (AAF55423) MNCKPLILCTFVAVAMCLVHFGNA---------- LPAISHYTHKRFDSMGGIDFVQVCLNNCVQCKTMLGDYFQGQ 66
Pediculus humanus corporis (EEB19572) MSAVCFLNNLLFVVVVTGMGMGLKKLYSILMAAVLLTTFLCLANSSILPTESNQFGICIRNCAQCKKMFGTYFEGQ 76
Ixodes scapularis (XP_002399271) MARILDFPIFLVVSAVAFAVLLSLSSA------------------- THTYPSDPVLVCINNCGQCKMIYGEYFNGR 57
Euphausia crystallorophias EH SCAESCIMSQGNSVPDCNNPSTFKNFLKRSSHHHYPRFVLAAP-------------------------- 92
Penaeus japonicus (CI998674) ACAESCIMTQGMSIPDCNNPSTFNRFLKRFI------------------------------------ 82
Penaeus monodon (GO073659) ACAESCIMTQGMSIPDCNNPSTFNRFLKRFI------------------------------------ 82
Callinectes sapidus (CV224237) LCGDFCLQTEGRFIPDCNRPDILIPFFLQRLE----------------------------------- 93
Balanus amphitrite (AFK81936) ACADACLA--GNEGPDCYNPSMVSRFLKRRASPVVPASPGLASLGGSTSTTAWTASRQRCPFPAARLSSECSQLEE 122
Daphnia pulex (EFX83453) LCAKSCFRLKGAFIPDCIDVASIGQFLNKNE------------------------------------ 64
Manduca sexta (AAA29310) LCAESCIKFKGKLIPECEDFASIAPFLNKL------------------------------------- 88
Drosophila melanogaster (AAF55423) TCALSCLKFKGKAIPDCEDIASIAPFLNALE------------------------------------ 97
Pediculus humanus corporis (EEB19572) LCADACVKFKGKVIPDCEDLDSISPFLNKVD------------------------------------ 107
Ixodes scapularis (XP_002399271) QCAEECLSTAGFIQPDCDEADSIVKYLRRKP------------------------------------ 88

Figure S9

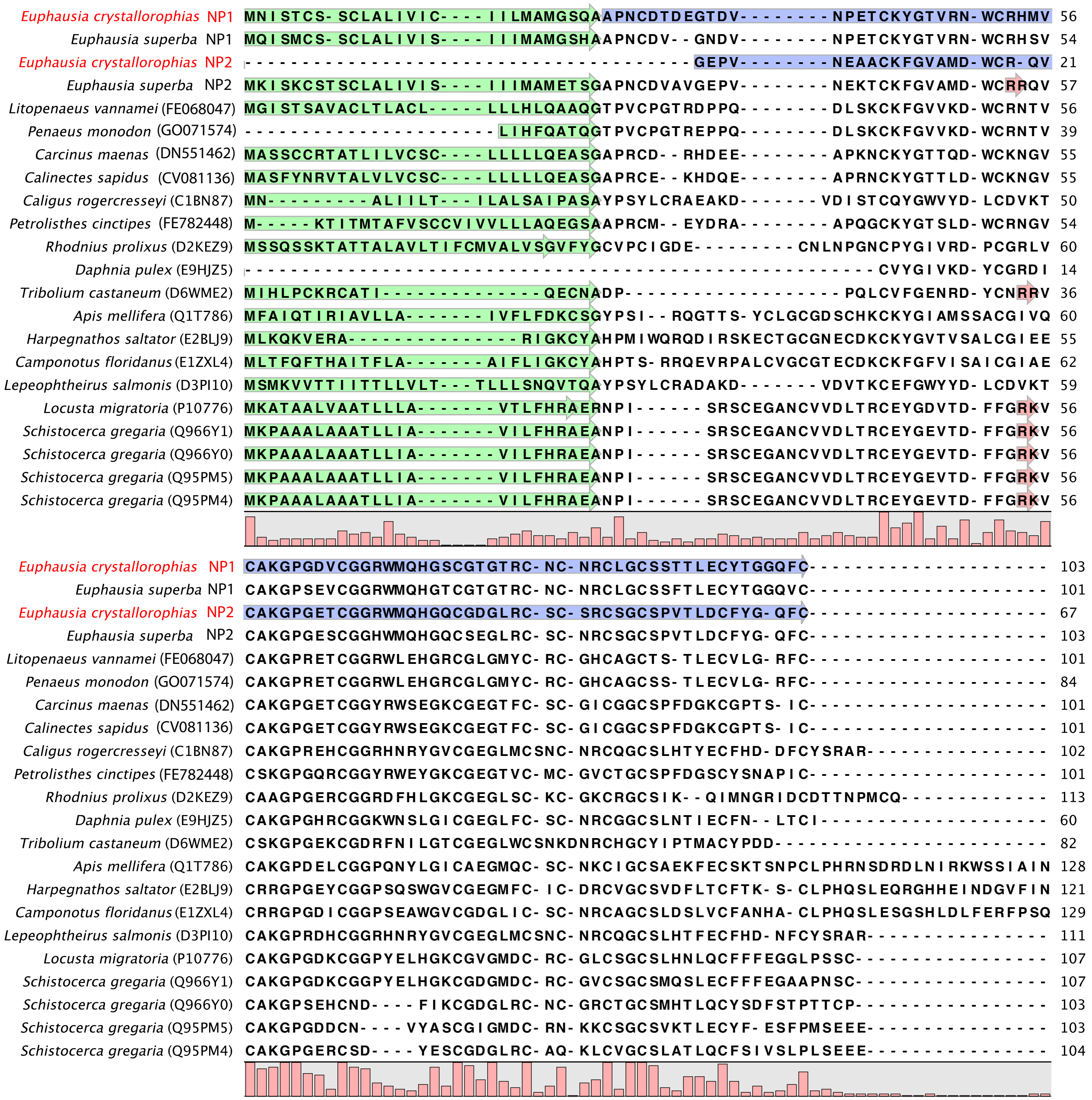

Euphausia crystallorophias NP1 MNISTCS-SCLALIVIC----IILMAMGSQAAPNCDTDEGTDV--------NPETCKYGTVRN-WCRHMV 56
Euphausia superba NP1 MQISMCS-SCLALIVIS----IIIMAMGSHAAPNCDV--GNDV--------NPETCKYGTVRN-WCRHSV 54
Euphausia crystallorophias NP2 ---------------------------------------GEPV--------NEAACKFGVAMD-WCR-QV 21
Euphausia superba NP2 MKISKCSTSCLALIVIS----IIIMAMETSGAPNCDVAVGEPV--------NEKTCKFGVAMD-WCRRQV 57
Litopenaeus vannamei (FE068047) MGISTSAVACLTLACL-----LLLHLQAAQGTPVCPGTRDPPQ--------DLSKCKFGVVKD-WCRNTV 56
Penaeus monodon (GO071574) ----------------------LIHFQATQGTPVCPGTREPPQ--------DLSKCKFGVVKD-WCRNTV 39
Carcinus maenas (DN551462) MASSCCRTATLILVCSC----LLLLLQEASGAPRCD--RHDEE--------APKNCKYGTTQD-WCKNGV 55
Calinectes sapidus (CV081136) MASFYNRVTALVLVCSC----LLLLLQEASGAPRCE--KHDQE--------APRNCKYGTTLD-WCKNGV 55
Caligus rogercresseyi (C1BN87) MN---------ALIILT---ILALSAIPASAYPSYLCRAEAKD-------VDISTCQYGWVYD-LCDVKT 50
Petrolisthes cinctipes (FE782448) M-----KTITMTAFVSCCVIVVLLLAQEGSAAPRCM--EYDRA--------APQGCKYGTSLD-WCRNGV 54
Rhodnius prolixus (D2KEZ9) MSSQSSKTATTALAVLTIFCMVALVSGVFYGCVPCIGDE---------CNLNPGNCPYGIVRD-PCGRLV 60
Daphnia pulex (E9HJZ5) ---CVYGIVKD-YCGRDI 14
Tribolium castaneum (D6WME2) MIHLPCKRCATI--------------QECNADP-------------------PQLCVFGENRD-YCNRRV 36
Apis mellifera (Q1T786) MFAIQTIRIAVLLA-------IVFLFDKCSGYPSI--RQGTTS-YCLGCGDSCHKCKYGIAMSSACGIVQ 60
Harpegnathos saltator (E2BLJ9) MLKQKVERA---------------RIGKCYAHPMIWQRQDIRSKECTGCGNECDKCKYGVTVSALCGIEE 55
Camponotus floridanus (E1ZXL4) MLTFQFTHAITFLA-------AIFLIGKCYAHPTS-RRQEVRPALCVGCGTECDKCKFGFVISAICGIAE 62
Lepeophtheirus salmonis (D3PI10) MSMKVVTTIITTLLVLT---TLLLSNQVTQAYPSYLCRADAKD-------VDVTKCEFGWYYD-LCDVKT 59
Locusta migratoria (P10776) MKATAALVAATLLLA-------VTLFHRAERNPI------SRSCEGANCVVDLTRCEYGDVTD-FFGRKV 56
Schistocerca gregaria (Q966Y1) MKPAAALAAATLLIA-------VILFHRAEANPI------SRSCEGANCVVDLTRCEYGEVTD-FFGRKV 56
Schistocerca gregaria (Q966Y0) MKPAAALAAATLLIA-------VILFHRAEANPI------SRSCEGANCVVDLTRCEYGEVTD-FFGRKV 56
Schistocerca gregaria (Q95PM5) MKPAAALAAATLLIA-------VILFHRAEANPI------SRSCEGANCVVDLTRCEYGEVTD-FFGRKV 56
Schistocerca gregaria (Q95PM4) MKPAAALAAATLLIA-------VILFHRAEANPI------SRSCEGANCVVDLTRCEYGEVTD-FFGRKV 56
Euphausia crystallorophias NP1 CAKGPGDVCGGRWMQHGSCGTGTRC-NC-NRCLGCSSTTLECYTGGQFC-------------------- 103
Euphausia superba NP1 CAKGPSEVCGGRWMQHGTCGTGTRC-NC-NRCLGCSSFTLECYTGGQVC-------------------- 101
Euphausia crystallorophias NP2 CAKGPGETCGGRWMQHGQCGDGLRC-SC-SRCSGCSPVTLDCFYG-QFC-------------------- 67
Euphausia superba NP2 CAKGPGESCGGHWMQHGQCSEGLRC-SC-NRCSGCSPVTLDCFYG-QFC-------------------- 103
Litopenaeus vannamei (FE068047) CAKGPRETCGGRWLEHGRCGLGMYC-RC-GHCAGCTS-TLECVLG-RFC-------------------- 101
Penaeus monodon (GO071574) CAKGPRETCGGRWLEHGRCGLGMYC-RC-GHCAGCSS-TLECVLG-RFC-------------------- 84
Carcinus maenas (DN551462) CAKGPGETCGGYRWSEGKCGEGTFC-SC-GICGGCSPFDGKCGPTS-IC-------------------- 101
Calinectes sapidus (CV081136) CAKGPGETCGGYRWSEGKCGEGTFC-SC-GICGGCSPFDGKCGPTS-IC-------------------- 101
Caligus rogercresseyi (C1BN87) CAKGPREHCGGRHNRYGVCGEGLMCSNC-NRCQGCSLHTYECFHD-DFCYSRAR--------------- 102
Petrolisthes cinctipes (FE782448) CSKGPGQRCGGYRWEYGKCGEGTVC-MC-GVCTGCSPFDGSCYSNAPIC-------------------- 101
Rhodnius prolixus (D2KEZ9) CAAGPGERCGGRDFHLGKCGEGLSC-KC-GKCRGCSIK--QIMNGRIDCDTTNPMCQ------------ 113
Daphnia pulex (E9HJZ5) CAKGPGHRCGGKWNSLGICGEGLFC-SC-NRCGGCSLNTIECFN--LTCI------------------- 60
Tribolium castaneum (D6WME2) CSKGPGEKCGDRFNILGTCGEGLWCSNKDNRCHGCYIPTMACYPDD----------------------- 82
Apis mellifera (Q1T786) CAKGPDELCGGPQNYLGICAEGMQC-SC-NKCIGCSAEKFECSKTSNPCLPHRNSDRDLNIRKWSSIAIN 128
Harpegnathos saltator (E2BLJ9) CRRGPGEYCGGPSQSWGVCGEGMFC-IC-DRCVGCSVDFLTCFTK-S-CLPHQSLEQRGHHEINDGVFIN 121
Camponotus floridanus (E1ZXL4) CRRGPGDICGGPSEAWGVCGDGLIC-SC-NRCAGCSLDSLVCFANHA-CLPHQSLESGSHLDLFERFPSQ 129
Lepeophtheirus salmonis (D3PI10) CAKGPRDHCGGRHNRYGVCGEGLMCSNC-NRCQGCSLHTFECFHD-NFCYSRAR--------------- 111
Locusta migratoria (P10776) CAKGPGDKCGGPYELHGKCGVGMDC-RC-GLCSGCSLHNLQCFFFEGGLPSSC---------------- 107
Schistocerca gregaria (Q966Y1) CAKGPGDKCGGPYELHGKCGDGMDC-RC-GVCSGCSMQSLECFFFEGAAPNSC---------------- 107
Schistocerca gregaria (Q966Y0) CAKGPSEHCND----FIKCGDGLRC-NC-GRCTGCSMHTLQCYSDFSTPTTCP---------------- 103
Schistocerca gregaria (Q95PM5) CAKGPGDDCN----VYASCGIGMDC-RN-KKCSGCSVKTLECYF-ESFPMSEEE--------------- 103
Schistocerca gregaria (Q95PM4) CAKGPGERCSD----YESCGDGLRC-AQ-KLCVGCSLATLQCFSIVSLPLSEEE--------------- 104

Figure S10

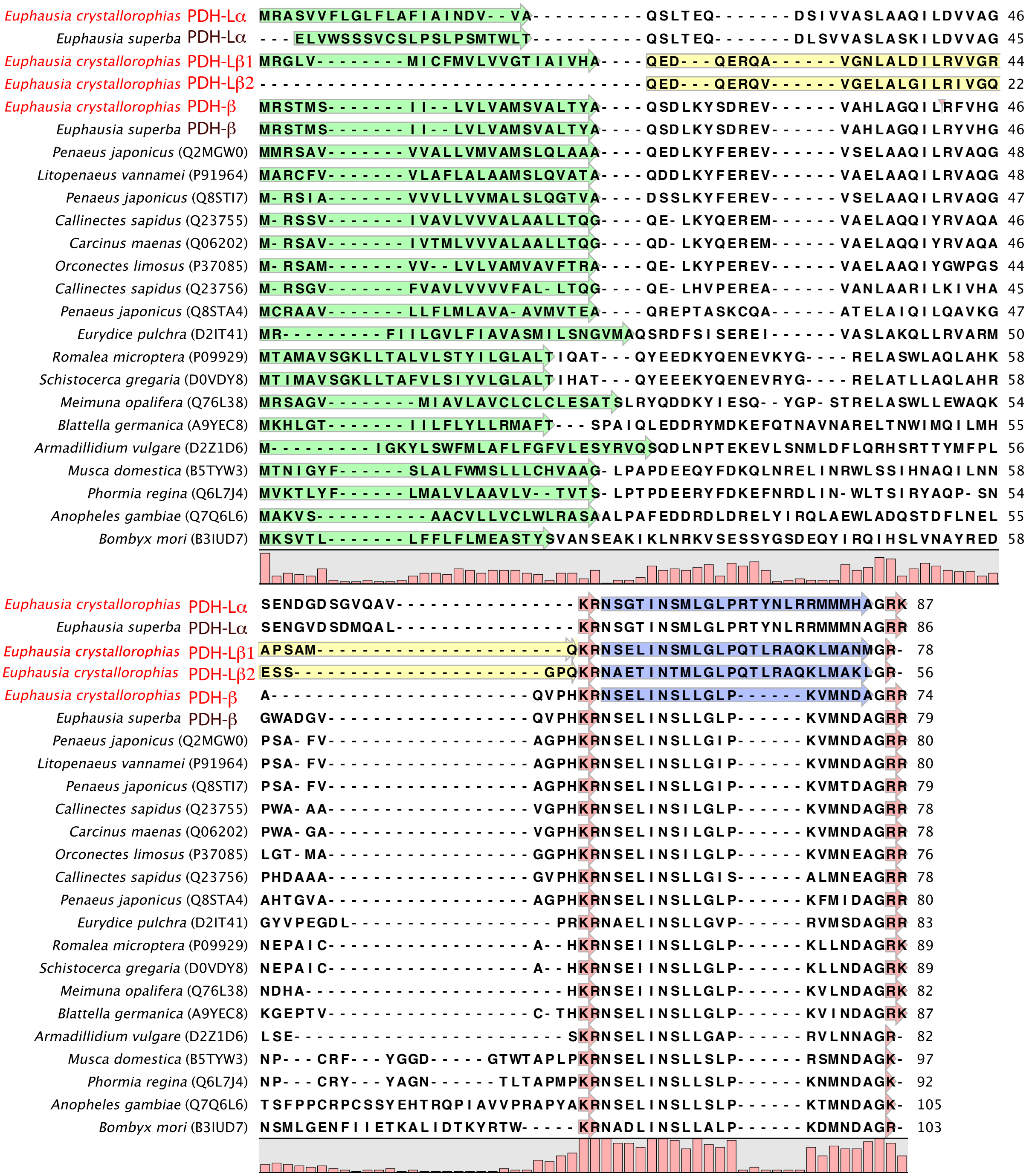

Euphausia crystallorophias PDH-Lα MRASVVFLGLFLAFIAINDV--VA----------QSLTEQ-------DSIVVASLAAQILDVVAG 46
Euphausia superba PDH-Lα ---ELVWSSSVCSLPSLPSMTWLT----------QSLTEQ-------DLSVVASLASKILDVVAG 45
Euphausia crystallorophias PDH-Lβ1 MRGLV--------MICFMVLVVGTIAIVHA----QED---QERQA------VGNLALDILRVVGR 44
Euphausia crystallorophias PDH-Lβ2 ----------------------------------QED---QERQV------VGELALGILRIVGQ 22
Euphausia crystallorophias PDH-β MRSTMS-------II--LVLVAMSVALTYA----QSDLKYSDREV------VAHLAGQILRFVHG 46
Euphausia superba PDH-β MRSTMS-------II--LVLVAMSVALTYA----QSDLKYSDREV------VAHLAGQILRYVHG 46
Penaeus japonicus (Q2MGW0) MMRSAV-------VVALLVMVAMSLQLAAA----QEDLKYFEREV------VSELAAQILRVAQG 48
Litopenaeus vannamei (P91964) MARCFV-------VLAFLALAAMSLQVATA----QDDLKYFEREV------VAELAAQILRVAQG 48
Penaeus japonicus (Q8STI7) M-RSIA-------VVVLLVVMALSLQGTVA----DSSLKYFEREV------VSELAAQILRVAQG 47
Callinectes sapidus (Q23755) M-RSSV-------IVAVLVVVALAALLTQG----QE-LKYQEREM------VAELAQQIYRVAQA 46
Carcinus maenas (Q06202) M-RSAV-------IVTMLVVVALAALLTQG----QD-LKYQEREM------VAELAQQIYRVAQA 46
Orconectes limosus (P37085) M-RSAM-------VV--LVLVAMVAVFTRA----QE-LKYPEREV------VAELAAQIYGWPGS 44
Callinectes sapidus (Q23756) M-RSGV-------FVAVLVVVVFAL-LTQG----QE-LHVPEREA------VANLAARILKIVHA 45
Penaeus japonicus (Q8STA4) MCRAAV-------LLFLMLAVA-AVMVTEA----QREPTASKCQA------ATELAIQILQAVKG 47
Eurydice pulchra (D2IT41) MR---------FIILGVLFIAVASMILSNGVMAQSRDFSISEREI------VASLAKQLLRVARM 50
Romalea microptera (P09929) MTAMAVSGKLLTALVLSTYILGLALTIQAT---QYEEDKYQENEVKYG----RELASWLAQLAHK 58
Schistocerca gregaria (D0VDY8) MTIMAVSGKLLTAFVLSIYVLGLALTIHAT---QYEEEKYQENEVRYG----RELATLLAQLAHR 58
Meimuna opalifera (Q76L38) MRSAGV--------MIAVLAVCLCLCLESATSLRYQDDKYIESQ--YGP-STRELASWLLEWAQK 54
Blattella germanica (A9YEC8) MKHLGT-------IILFLYLLRMAFT---SPAIQLEDDRYMDKEFQTNAVNARELTNWIMQILMH 55
Armadillidium vulgare (D2Z1D6) M---------IGKYLSWFMLAFLFGFVLESYRVQSQDLNPTEKEVLSNMLDFLQRHSRTTYMFPL 56
Musca domestica (B5TYW3) MTNIGYF------SLALFWMSLLLCHVAAG-LPAPDEEQYFDKQLNRELINRWLSSIHNAQILNN 58
Phormia regina (Q6L7J4) MVKTLYF------LMALVLAAVLV--TVTS-LPTPDEERYFDKEFNRDLIN-WLTSIRYAQP-SN 54
Anopheles gambiae (Q7Q6L6) MAKVS----------AACVLLVCLWLRASAALPAFEDDRDLDRELYIRQLAEWLADQSTDFLNEL 55
Bombyx mori (B3IUD7) MKSVTL-------LFFLFLMEASTYSVANSEAKIKLNRKVSESSYGSDEQYIRQIHSLVNAYRED 58

Euphausia crystallorophias PDH-Lα SENDGDSGVQAV----------------KRNSGTINSMLGLPRTYNLRRMMMHAGRK 87
Euphausia superba PDH-Lα SENGVDSDMQAL----------------KRNSGTINSMLGLPRTYNLRRMMMNAGRR 86
Euphausia crystallorophias PDH-Lβ1 APSAM----------------------QKRNSELINSMLGLPQTLRAQKLMANMGR- 78
Euphausia crystallorophias PDH-Lβ2 ESS----------------------GPQKRNAETINTMLGLPQTLRAQKLMAKLGR- 56
Euphausia crystallorophias PDH-β A-----------------------QVPHKRNSELINSLLGLP------KVMNDAGRR 74
Euphausia superba PDH-β GWADGV------------------QVPHKRNSELINSLLGLP------KVMNDAGRR 79
Penaeus japonicus (Q2MGW0) PSA-FV------------------AGPHKRNSELINSLLGIP------KVMNDAGRR 80
Litopenaeus vannamei (P91964) PSA-FV------------------AGPHKRNSELINSLLGIP------KVMNDAGRR 80
Penaeus japonicus (Q8STI7) PSA-FV------------------AGPHKRNSELINSLLGIP------KVMTDAGRR 79
Callinectes sapidus (Q23755) PWA-AA------------------VGPHKRNSELINSILGLP------KVMNDAGRR 78
Carcinus maenas (Q06202) PWA-GA------------------VGPHKRNSELINSILGLP------KVMNDAGRR 78
Orconectes limosus (P37085) LGT-MA------------------GGPHKRNSELINSILGLP------KVMNEAGRR 76
Callinectes sapidus (Q23756) PHDAAA------------------GVPHKRNSELINSLLGIS------ALMNEAGRR 78
Penaeus japonicus (Q8STA4) AHTGVA------------------AGPHKRNSELINSLLGLP------KFMIDAGRR 80
Eurydice pulchra (D2IT41) GYVPEGDL------------------PRKRNAELINSLLGVP------RVMSDAGRR 83
Romalea microptera (P09929) NEPAIC------------------A--HKRNSEIINSLLGLP------KLLNDAGRK 89
Schistocerca gregaria (D0VDY8) NEPAIC------------------A--HKRNSEIINSLLGLP------KLLNDAGRK 89
Meimuna opalifera (Q76L38) NDHA-----------------------HKRNSEIINSLLGLP------KVLNDAGRK 82
Blattella germanica (A9YEC8) KGEPTV------------------C-THKRNSELINSLLGLP------KVINDAGRK 87
Armadillidium vulgare (D2Z1D6) LSE------------------------SKRNSELINSLLGAP------RVLNNAGR- 82
Musca domestica (B5TYW3) NP---CRF---YGGD-----GTWTAPLPKRNSELINSLLSLP------RSMNDAGK- 97
Phormia regina (Q6L7J4) NP---CRY---YAGN------TLTAPMPKRNSELINSLLSLP------KNMNDAGK- 92
Anopheles gambiae (Q7Q6L6) TSFPPCRPCSSYEHTRQPIAVVPRAPYAKRNSELINSLLSLP------KTMNDAGK- 105
Bombyx mori (B3IUD7) NSMLGENFIIETKALIDTKYRTW-----KRNADLINSLLALP------KDMNDAGR- 103

Figure S11a

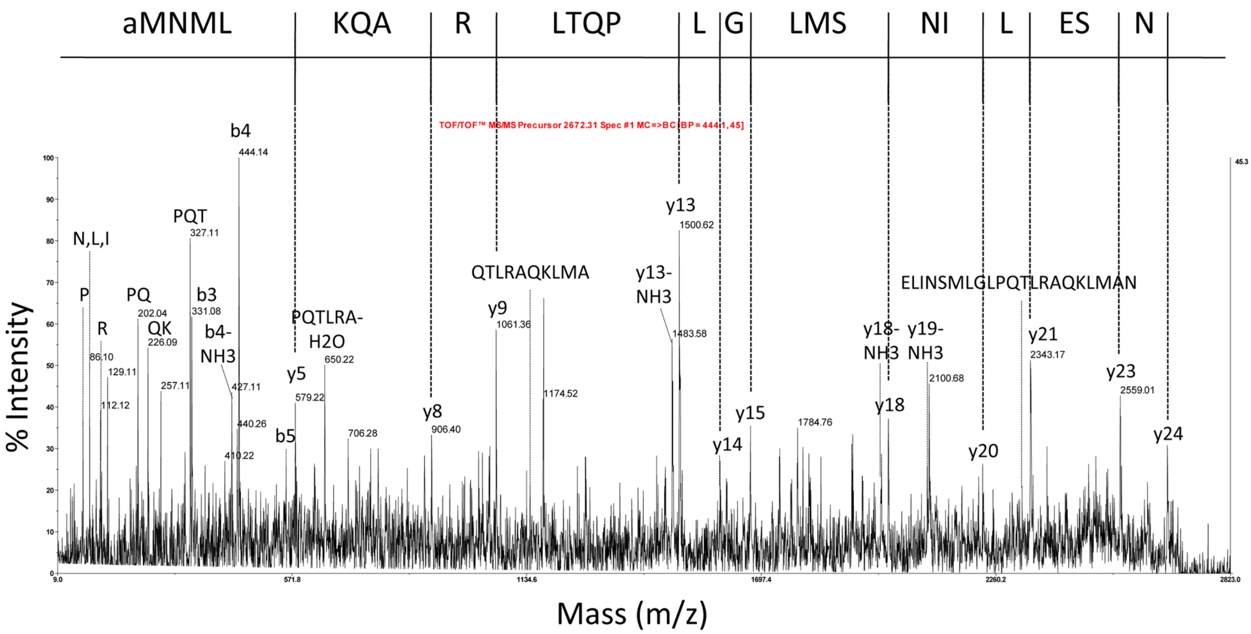

Figure S11b NSELINSMLGLPQTLRAQKLMANMa

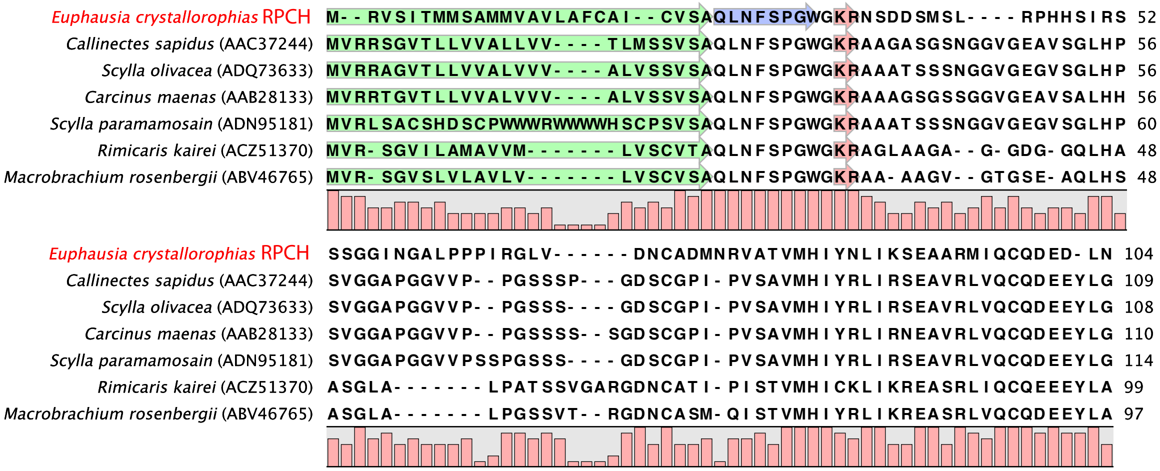

Euphausia crystallorophias RPCH M--RVSITMMSAMMVAVLAFCAI--CVSAQLNFSPGWGKRNSDDSMSL----RPHHSIRS 52
Callinectes sapidus (AAC37244) MVRRSGVTLLVVALLVV----TLMSSVSAQLNFSPGWGKRAAGASGSNGGVGEAVSGLHP 56
Scylla olivacea (ADQ73633) MVRRAGVTLLVVALVVV----ALVSSVSAQLNFSPGWGKRAAATSSSNGGVGEGVSGLHP 56
Carcinus maenas (AAB28133) MVRRTGVTLLVVALVVV----ALVSSVSAQLNFSPGWGKRAAAGSGSSGGVGEAVSALHH 56
Scylla paramamosain (ADN95181) MVRLSACSHDSCPWWWRWWWWHSCPSVSAQLNFSPGWGKRAAATSSSNGGVGEGVSGLHP 60
Rimicaris kairei (ACZ51370) MVR-SGVILAMAVVM-------LVSCVTAQLNFSPGWGKRAGLAAGA--G-GDG-GQLHA 48
Macrobrachium rosenbergii (ABV46765) MVR-SGVSLVLAVLV-------LVSCVSAQLNFSPGWGKRAA-AAGV--GTGSE-AQLHS 48
Euphausia crystallorophias RPCH SSGGINGALPPPIRGLV------DNCADMNRVATVMHIYNLIKSEAARMIQCQDED-LN 104
Callinectes sapidus (AAC37244) SVGGAPGGVVP--PGSSSP---GDSCGPI-PVSAVMHIYRLIRSEAVRLVQCQDEEYLG 109
Scylla olivacea (ADQ73633) SVGGAPGGVVP--PGSSS----GDSCGPI-PVSAVMHIYRLIRSEAVRLVQCQDEEYLG 108
Carcinus maenas (AAB28133) SVGGAPGGVVP--PGSSSS--SGDSCGPI-PVSAVMHIYRLIRNEAVRLVQCQDEEYLG 110
Scylla paramamosain (ADN95181) SVGGAPGGVVPSSPGSSS----GDSCGPI-PVSAVMHIYRLIRSEAVRLVQCQDEEYLG 114
Rimicaris kairei (ACZ51370) ASGLA-------LPATSSVGARGDNCATI-PISTVMHICKLIKREASRLIQCQEEEYLA 99
Macrobrachium rosenbergii (ABV46765) ASGLA-------LPGSSVT--RGDNCASM-QISTVMHIYRLIKREASRLVQCQDEEYLA 97

Figure S12a

Helicoverpa zea (P67787; P08901)	QLTFTSSWG-	9
Manduca sexta (P67788; P08901)	QLTFTSSWG-	9
Drosophila melanogaster (P61855; P17975; Q9VZH6)	QLTFSPDWG-	9
Protophormia terraenovae (P61856; P17975; Q9VZH6)	QLTFSPDW- -	8
Tabanus atratus (P14595)	QLTFTPGW- -	8
Libellula auripennis (P25418)	QVNFTPSW- -	8
Locusta migratoria (P19872)	QLNFTPWWG-	9
Locusta migratoria (P55319; P01271; P10616)	QLNFTPNWGT	10
Schistocerca gregaria (P18829)	QLNFTPNWGT	10
Anoplotrupes stercorosus (P84241; P25423)	QLNYSPDW- -	8
Pachnoda marginata (P84242; P25423)	QLNYSPDW- -	8
Melolontha melolontha (P84240; P25423)	QLNYSPDW- -	8
Metacarcinus magister (1812292A)	ELNFSPGW- -	8
Euphausia crystallorophias RPCH	QLNFSPGW- -	8
Carcinus maenas (Q26324)	QLNFSPGW- -	8
Callinectes sapidus (AAC37244)	QLNFSPGW- -	8
Scylla paramamosain (ADN95181)	QLNFSPGW- -	8
Scylla olivacea (ADQ73633)	QLNFSPGW- -	8
Macrobrachium rosenbergii (ABV46765)	QLNFSPGW- -	8
Rimicaris kairei (ACZ51370)	QLNFSPGW- -	8
Pandalus borealis (P08939)	QLNFSPGW- -	8
Blatta orientalis (P84261; P04548)	QVNFSPNW- -	8
Periplaneta americana (P84259; P04548)	QVNFSPNW- -	8
Deropeltis integerrima (P85591)	QVNFSPNW- -	8
Eurycotis floridana (P85634)	QVNFSPNW- -	8
Blatta lateralis (P85855)	QVNFSPNW- -	8
Deropeltis atra (P85597)	QVNFSPNW- -	8
Deropeltis erythrocephala (P85585)	QVNFSPNW- -	8
Cryptocercus darwini (P85570)	QLNFSPNW- -	8
Cryptocercus kyebangensis (P85575)	QLNFSPNW- -	8
Ergaula capucina (P85612)	QLNFSPNW- -	8
Locusta migratoria (P08379; P10617)	QLNFSAGWG-	9
Schistocerca gregaria (P35808; P08378)	QLNFSTGWG-	9
Schistocerca nitens (P35807; P08378)	QLNFSTGWG-	9
Daphnia pulex (EFX68649)	QVNFSTSWG-	9
Romalea microptera (P67786; P14086)	QVNFSTGW- -	8
Gryllus bimaculatus (P67785; P14086)	QVNFSTGW- -	8
Archimandrita tessellata (P85538)	QVNFSPGWGT	10
Aptera fusca (P85533)	QVNFSPGWGT	10
Bantua robusta (P85543)	QVNFSPGWGT	10
Blaptica dubia (P85549)	QVNFSPGWGT	10
Loboptera decipiens (P85846)	QVNFSPGWGT	10
Lucihormetica grossei (P85664)	QVNFSPGWGT	10
Blaberus craniifer (P85546)	QVNFSPGWGT	10
Blattella germanica (P84220; P10939)	QVNFSPGWGT	10
Blaberus giganteus (P85557)	QVNFSPGWGT	10
Blepharodera discoidalis (P85565)	QVNFSPGWGT	10
Diploptera punctata (P85602)	QVNFSPGWGT	10
Elliptorhina sp. (P85851)	QVNFSPGWGT	10
Eublaberus distanti (P85618)	QVNFSPGWGT	10
Eublaberus posticus (P85624)	QVNFSPGWGT	10
Gromphadorina portentosa (P84221; P10939)	QVNFSPGWGT	10
Gyna lurida (P85651)	QVNFSPGWGT	10
Gromphadorhina grandidieri (P85639)	QVNFSPGWGT	10

Conservation

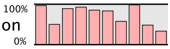

Figure S12b

Euphausia crystallorophias ASTC	-----SYWKQCAFNAVSCF	14
Euphausia superba ASTC	-----SYWKQCAFNA----	10
Homarus americanus (EY291152)	-----SYWKQCAFNAVSCF	14
Daphnia pulex (EFX85706)	-----SYWKQCAFNAVSCF	14
Laupala kohalensis	-----SYWKQCAFNAVSCF	14
Locusta migratoria (CO820847)	-----SYWKQCAFNAVSCF	14
Daphnia pulex	GQSSQRVFWRCYFNAVSCF	19
Litopenaeus vannamei (FE182974)	----QIRYHQCYFNPISCF	15
Cancer borealis	----QIRYHQCYFNPISCF	15
Drosophila melanogaster (AAK40081)	----QVRYRQCYFNPISCF	15
Tribolium castaneum (EFA09152)	----QSRYRQCYFNPISCF	15
Bombyx mori (BAG68396)	----QVRFRQCYFNPISCF	15
Spodoptera frugiperda (CAD32496)	----QVRFRQCYFNPISCF	15
Samia cynthia ricini (AAY68369)	----QVRFRQCYFNPISCF	15
Manduca sexta (P42559)	----QVRFRQCYFNPISCF	15

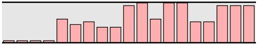

Figure S13

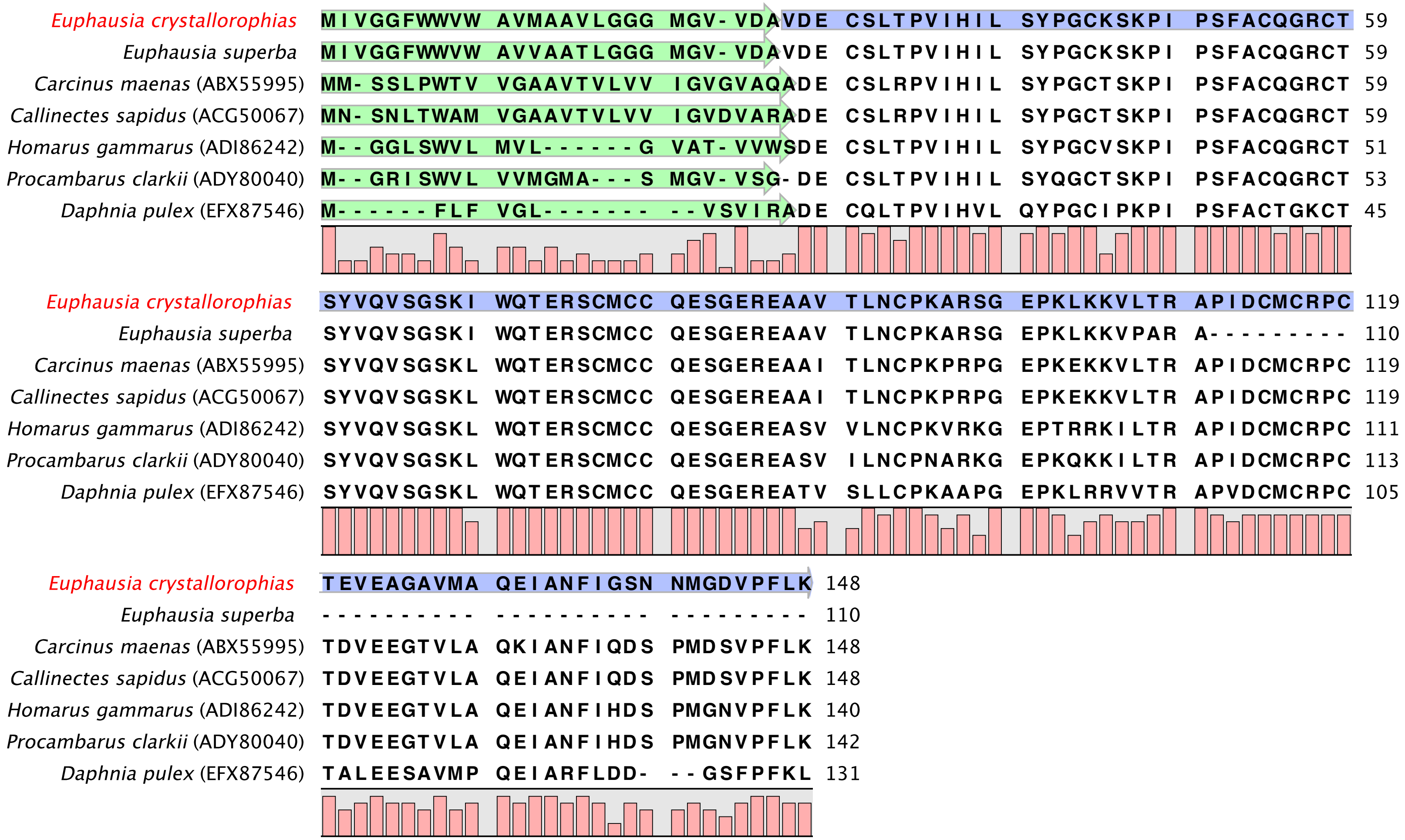
Euphausia crystallorophias MIVGGFWWVW AVMAAVLGGG MGV-VDAVDE CSLTPVIHIL SYPGCKSKPI PSFACQGRCT 59
Euphausia superba MIVGGFWWVW AVVAATLGGG MGV-VDAVDE CSLTPVIHIL SYPGCKSKPI PSFACQGRCT 59
Carcinus maenas (ABX55995) MM-SSLPWTV VGAAVTVLVV IGVGVAQADE CSLRPVIHIL SYPGCTSKPI PSFACQGRCT 59
Callinectes sapidus (ACG50067) MN-SNLTWAM VGAAVTVLVV IGVDVARADE CSLRPVIHIL SYPGCTSKPI PSFACQGRCT 59
Homarus gammarus (ADI86242) M--GGLSWVL MVL------G VAT-VVWSDE CSLTPVIHIL SYPGCVSKPI PSFACQGRCT 51
Procambarus clarkii (ADY80040) M--GRISWVL VVMGMA---S MGV-VSG-DE CSLTPVIHIL SYQGCTSKPI PSFACQGRCT 53
Daphnia pulex (EFX87546) M-----FLF VGL------- --VSVIRADE CQLTPVIHVL QYPGCIPKPI PSFACTGKCT 45
Euphausia crystallorophias SYVQVSGSKI WQTERSCMCC QESGEREAAV TLNCPKARSG EPKLKKVLTR APIDCMCRPC 119
Euphausia superba SYVQVSGSKI WQTERSCMCC QESGEREAAV TLNCPKARSG EPKLKKVPAR A--------- 110
Carcinus maenas (ABX55995) SYVQVSGSKL WQTERSCMCC QESGEREAAI TLNCPKPRPG EPKEKKVLTR APIDCMCRPC 119
Callinectes sapidus (ACG50067) SYVQVSGSKL WQTERSCMCC QESGEREAAI TLNCPKPRPG EPKEKKVLTR APIDCMCRPC 119
Homarus gammarus (ADI86242) SYVQVSGSKL WQTERSCMCC QESGEREASV VLNCPKVRKG EPTRRKILTR APIDCMCRPC 111
Procambarus clarkii (ADY80040) SYVQVSGSKL WQTERSCMCC QESGEREASV ILNCPNARKG EPKQKKILTR APIDCMCRPC 113
Daphnia pulex (EFX87546) SYVQVSGSKL WQTERSCMCC QESGEREATV SLLCPKAAPG EPKLRRVVTR APVDCMCRPC 105
Euphausia crystallorophias TEVEAGAVMA QEIANFIGSN NMGDVPFLK 148
Euphausia superba ---------- ---------- --------- 110
Carcinus maenas (ABX55995) TDVEEGTVLA QKIANFIQDS PMDSVPFLK 148
Callinectes sapidus (ACG50067) TDVEEGTVLA QEIANFIQDS PMDSVPFLK 148
Homarus gammarus (ADI86242) TDVEEGTVLA QEIANFIHDS PMGNVPFLK 140
Procambarus clarkii (ADY80040) TDVEEGTVLA QEIANFIHDS PMGNVPFLK 142
Daphnia pulex (EFX87546) TALEESAVMP QEIARFLDD- --GSFPFKL 131

Figure S14a

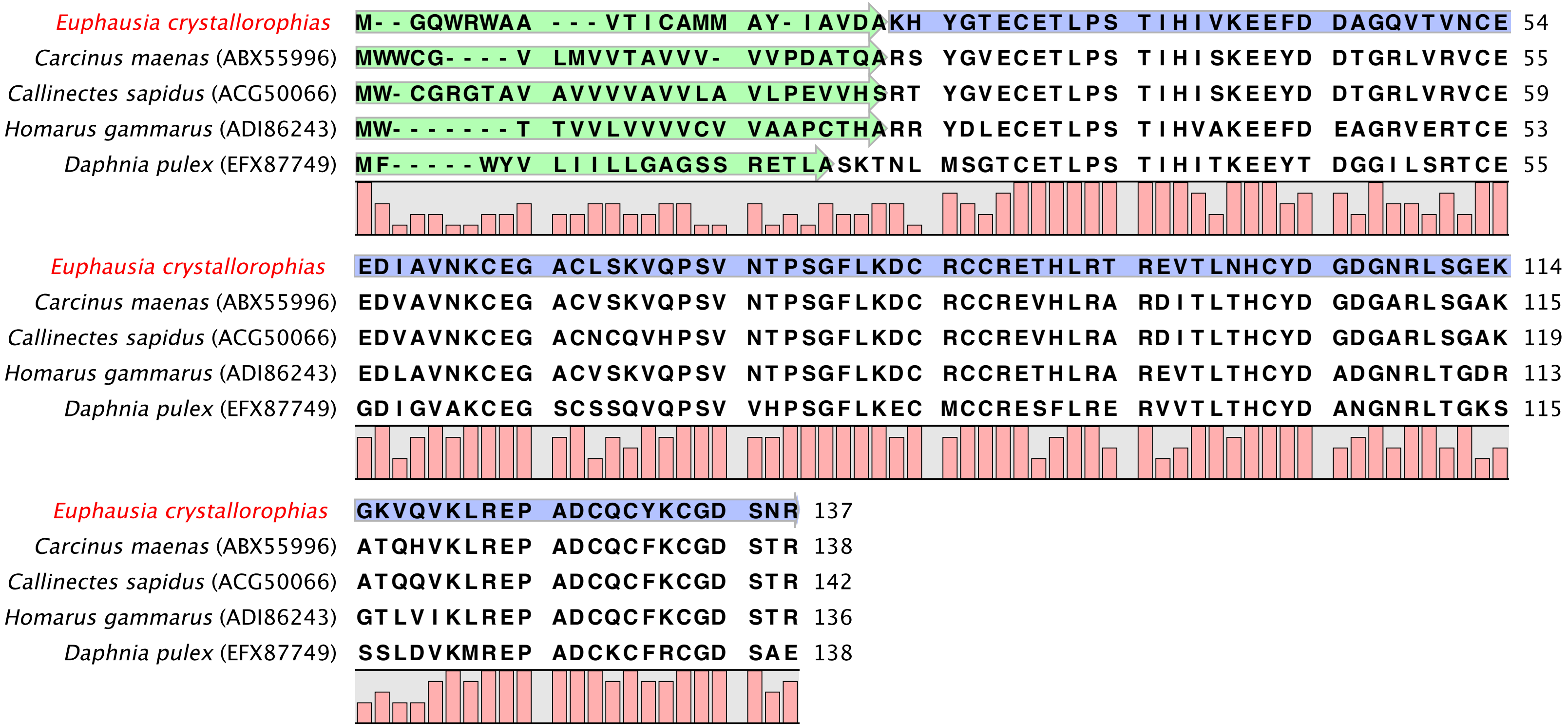
Euphausia crystallorophias M- - GQWRWAA - - - VT I CAMM AY- I AVDAKH YGT ECETLPS T I H I VKEEFD DAGQVTVNCE 54
Carcinus maenas (ABX55996) MWWCG- - - - V LMVVTAVVV- VVPDATQARS YGVECETLPS T I H I SKEEYD DTGRLVRVCE 55
Callinectes sapidus (ACG50066) MW- CGRGTAV AVVVVAVVLA VLPEVVHSRT YGVECETLPS T I H I SKEEYD DTGRLVRVCE 59
Homarus gammarus (ADI86243) MW- - - - - - - T TVVLVVVVCV VAAPCTHARR YDLECETLPS T I HVAKEEFD EAGRVERTCE 53
Daphnia pulex (EFX87749) MF- - - - - WYV L I I LLGAGSS RETLASKTNL MSGTCETLPS T I H I TKEEYT DGGI LSRTCE 55
Euphausia crystallorophias ED I AVNKCEG ACLSKVQPSV NTPSGFLKDC RCCRETHLRT REVTLNHCYD GDGNRLSGEK 114
Carcinus maenas (ABX55996) EDVAVNKCEG ACVSKVQPSV NTPSGFLKDC RCCREVHLRA RD I TLTHCYD GDGARLSGAK 115
Callinectes sapidus (ACG50066) EDVAVNKCEG ACNCQVHPSV NTPSGFLKDC RCCREVHLRA RD I TLTHCYD GDGARLSGAK 119
Homarus gammarus (ADI86243) EDLAVNKCEG ACVSKVQPSV NTPSGFLKDC RCCRETHLRA REVTLTHCYD ADGNRLTGDR 113
Daphnia pulex (EFX87749) GD I GVAKCEG SCSSQVQPSV VHPSGFLKEC MCCRESFLRE RVVTLTHCYD ANGNRLTGKS 115
Euphausia crystallorophias GKVQVKLREP ADCQCYKCGD SNR 137
Carcinus maenas (ABX55996) ATQHVKLREP ADCQCFKCGD STR 138
Callinectes sapidus (ACG50066) ATQQVKLREP ADCQCFKCGD STR 142
Homarus gammarus (ADI86243) GTLV I KLREP ADCQCFKCGD STR 136
Daphnia pulex (EFX87749) SSLDVKMREP ADCKCFRCGD SAE 138

Figure S14b

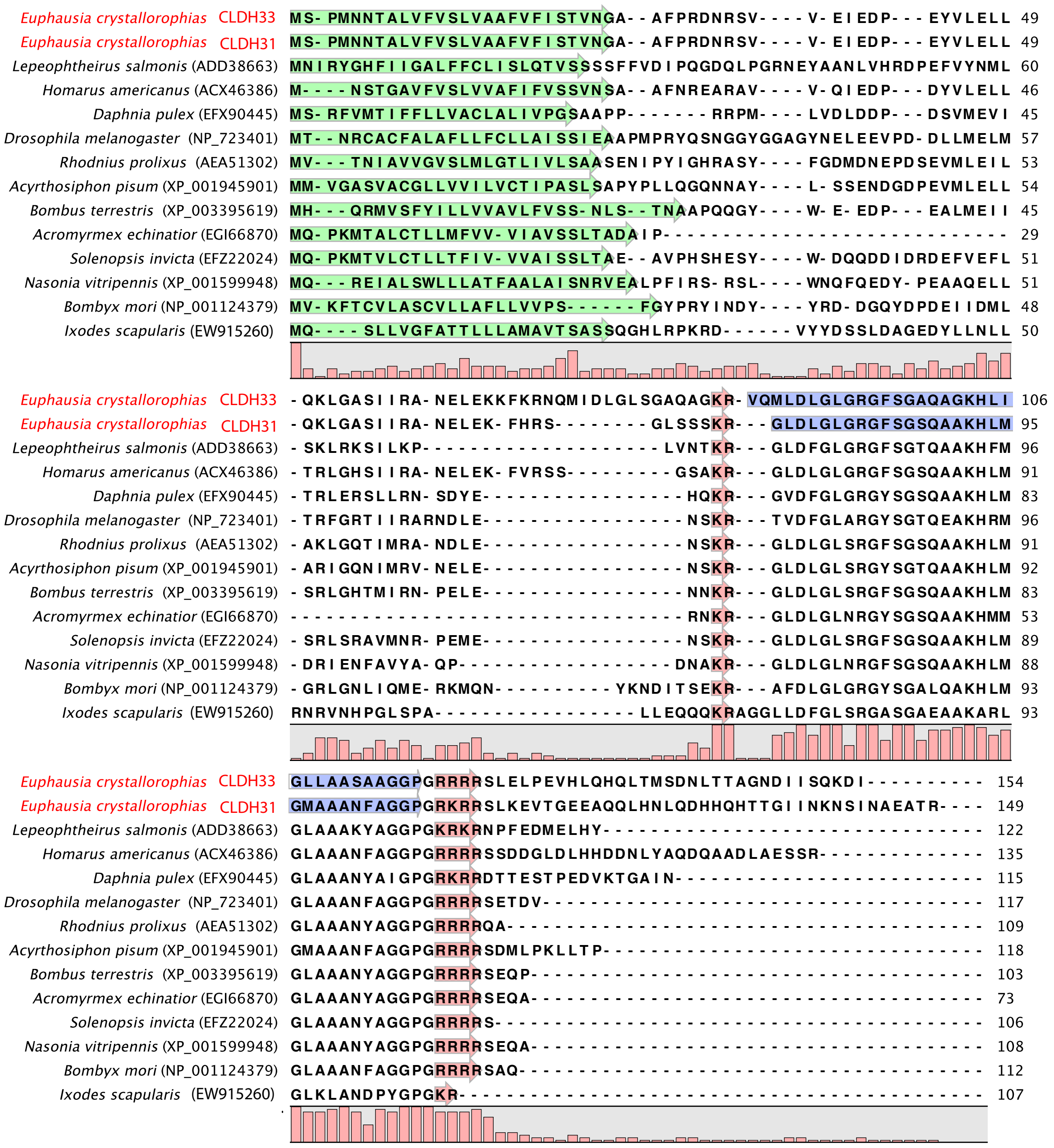

Figure S15

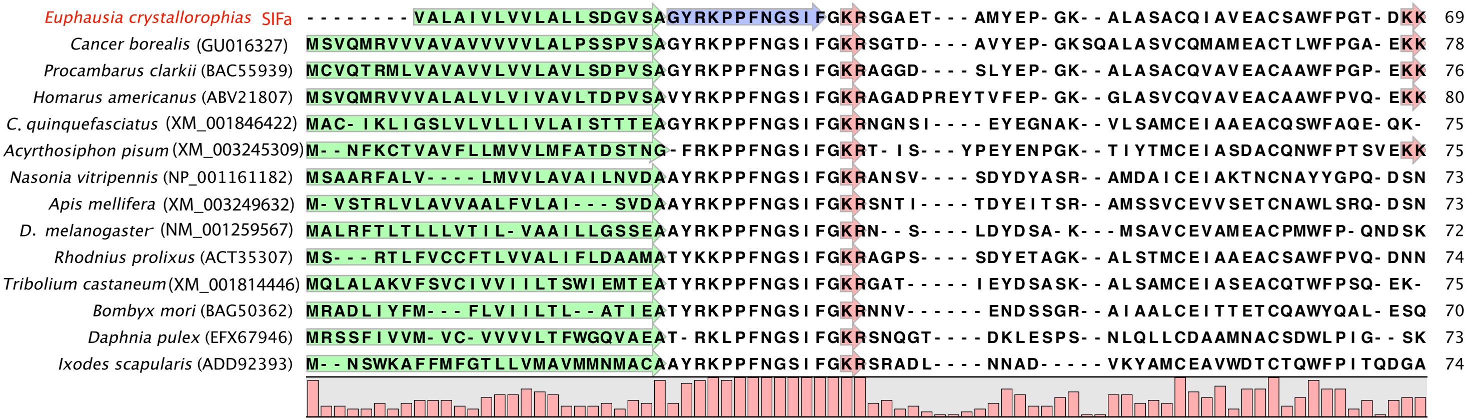

Euphausia crystallorophias SIFa --------VALAIVLVVLALLSDGVSAGYRKPPFNGSIFGKRSGAET---AMYEP-GK--ALASACQIAVEACSAWFPGT-DKK 69
Cancer borealis (GU016327) MSVQMRVVVAVAVVVVVLALPSSPVSAGYRKPPFNGSIFGKRSGTD----AVYEP-GKSQALASVCQMAMEACTLWFPGA-EKK 78
Procambarus clarkii (BAC55939) MCVQTRMLVAVAVVLVVLAVLSDPVSAGYRKPPFNGSIFGKRAGGD----SLYEP-GK--ALASACQVAVEACAAWFPGP-EKK 76
Homarus americanus (ABV21807) MSVQMRVVVALALVLVIVAVLTDPVSAVYRKPPFNGSIFGKRAGADPREYTVFEP-GK--GLASVCQVAVEACAAWFPVQ-EKK 80
C. quinquefasciatus (XM_001846422) MAC-IKLIGSLVLVLLIVLAISTTTEAGYRKPPFNGSIFGKRNGNSI----EYEGNAK--VLSAMCEIAAEACQSWFAQE-QK- 75
Acyrthosiphon pisum (XM_003245309) M--NFKCTVAVFLLMVVLMFATDSTNG-FRKPPFNGSIFGKRT-IS---YPEYENPGK--TIYTMCEIASDACQNWFPTSVEKK 75
Nasonia vitripennis (NP_001161182) MSAARFALV----LMVVLAVAILNVDAAYRKPPFNGSIFGKRANSV----SDYDYASR--AMDAICEIAKTNCNAYYGPQ-DSN 73
Apis mellifera (XM_003249632) M-VSTRLVLAVVAALFVLAI---SVDAAYRKPPFNGSIFGKRSNTI----TDYEITSR--AMSSVCEVVSETCNAWLSRQ-DSN 73
D. melanogaster (NM_001259567) MALRFTLTLLLVTIL-VAAILLGSSEAAYRKPPFNGSIFGKRN--S----LDYDSA-K---MSAVCEVAMEACPMWFP-QNDSK 72
Rhodnius prolixus (ACT35307) MS---RTLFVCCFTLVVALIFLDAAMATYKKPPFNGSIFGKRAGPS----SDYETAGK--ALSTMCEIAAEACSAWFPVQ-DNN 74
Tribolium castaneum (XM_001814446) MQLALAKVFSVCIVVIILTSWIEMTEATYRKPPFNGSIFGKRGAT-----IEYDSASK--ALSAMCEIASEACQTWFPSQ-EK- 75
Bombyx mori (BAG50362) MRADLIYFM---FLVIILTL--ATIEATYRKPPFNGSIFGKRNNV------ENDSSGR--AIAALCEITTETCQAWYQAL-ESQ 70
Daphnia pulex (EFX67946) MRSSFIVVM-VC-VVVVLTFWGQVAEAT-RKLPFNGSIFGKRSNQGT---DKLESPS--NLQLLCDAAMNACSDWLPIG--SK 73
Ixodes scapularis (ADD92393) M--NSWKAFFMFGTLLVMAVMMNMACAAYRKPPFNGSIFGKRSRADL---NNAD-----VKYAMCEAVWDTCTQWFPITQDGA 74

Figure S16
